# Enantioselective bifunctional iminophosphorane catalyzed sulfa-Michael addition of alkyl thiols to unactivated β-substituted-α,β-unsaturated esters†
†Electronic supplementary information (ESI) available: Experimental procedures, spectroscopic data, copies of ^1^H and ^13^C NMR spectra and HPLC and GC chromatograms. See DOI: 10.1039/c6sc02878k



**DOI:** 10.1039/c6sc02878k

**Published:** 2016-09-14

**Authors:** Jinchao Yang, Alistair J. M. Farley, Darren J. Dixon

**Affiliations:** a Department of Chemistry , Chemistry Research Laboratory , University of Oxford , 12 Mansfield Road , Oxford , UK . Email: Darren.dixon@chem.ox.ac.uk

## Abstract

The highly enantioselective sulfa-Michael addition of alkyl thiols to unactivated β-substituted-α,β-unsaturated esters catalyzed by a bifunctional iminophosphorane (BIMP) organocatalyst is described. The low acidity of the alkyl thiol pro-nucleophiles is overcome by the high Brønsted basicity of the catalyst and the chiral scaffold/thiourea hydrogen-bond donor moiety provides the required enantiofacial discrimination in the addition step. The reaction is broad in scope with respect to the alkyl thiol and β-substituent of the α,β-unsaturated ester, affords sulfa-Michael adducts in excellent yields (up to >99%) and enantioselectivity (up to 97 : 3 er) and can operate down to 1 mol% catalyst loading.

## Abstract 2

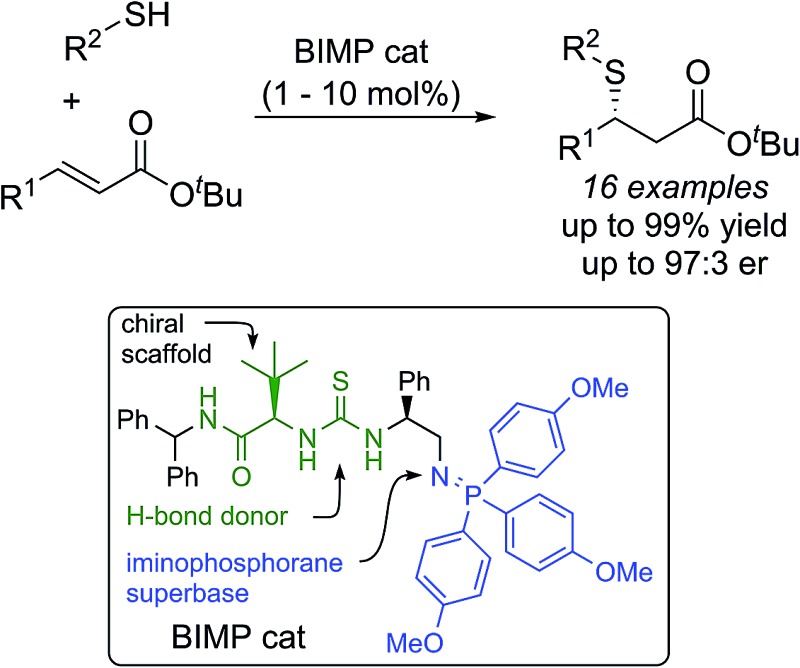
The highly enantioselective sulfa-Michael addition of alkyl thiols to unactivated β-substituted-α,β-unsaturated esters catalyzed by a bifunctional iminophosphorane (BIMP) organocatalyst is described.

Unactivated β-substituted-α,β-unsaturated esters, such as methyl crotonate, methyl cinnamate and their homologues, are a class of low reactivity electrophiles that offer a wealth of untapped potential in the field of enantioselective organocatalysis.^1^ To date, these esters have remained a persistent challenge as Michael acceptors in asymmetric catalysis using both metal-rich and metal-free catalyst systems, largely due to their low inherent electrophilicity^2^ and low propensity for catalyst activation and enantioface discrimination.^3,4^ They are commercial and cheap, or are readily prepared by a variety of standard methods and are stable. In contrast to commonly used (reactive) Michael acceptors such as nitroolefins, they lie at the bottom of the Mayr electrophile reactivity (*E*) scale,^5,6^ and unlike enal and enone Michael acceptors they cannot be activated through iminium ion formation with chiral amine catalysts.^7^ Related literature examples employ activated carboxylic derivatives^8^ such as *N*-enoyl imides, *N*-enoyl oxazolidinones, perfluorinated alkyl esters, thioamides, *N*-enoyl pyrroles and, most recently, aryl esters.^9^ Alternatively, activating substituents at the α- or β-positions can also be used to gain reactivity and/or stereoselectivity. To illustrate the case in point, to date there has not been a single report of a highly enantioselective addition of a pro-nucleophilic reagent [a carbon-centered (C–H) or heteroatom-centered (X–H) acid] to unactivated alkyl cinnamate or crotonate esters under organocatalytic conditions.^10^ Effectively, these cheap chemical feedstocks are out of reach of existing chiral organocatalysts and accordingly are a very attractive ‘simple’ target class of electrophiles for new enantioselective organocatalytic reaction development (Fig. 1).

**Fig. 1:**
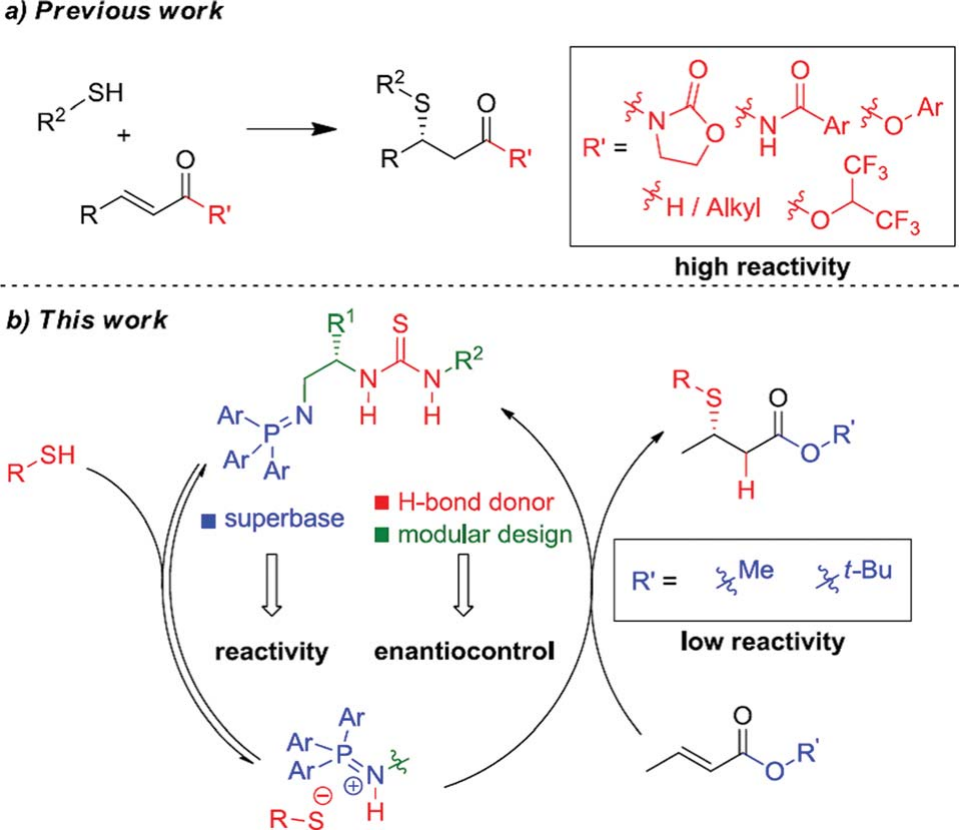
Bifunctional Brønsted base/H-bond donor organocatalytic SMA to α,β-unsaturated ester derivatives.

A proven strategy to overcome low substrate electrophilicity in base-catalyzed polar addition reactions is to increase the concentration of the nucleophilic conjugate base in the pot – and therefore the rate of the nucleophilic addition reaction – by enhancing the Brønsted basicity of the catalyst relative to tertiary amine catalysts.^11–13^ To this end, we disclosed that bifunctional iminophosphorane (BIMP) catalysts, containing a novel organosuperbase were highly efficacious in the first general enantioselective organocatalytic ketimine nitro-Mannich reaction.^12b,d^ Likewise, very recently, high catalyst performance (in terms of reactivity and enantioselectivity) with a second generation BIMP catalyst was also witnessed in the first organocatalytic conjugate addition of alkyl thiols to unactivated α-substituted acrylate esters (such as methyl methacrylate).^12*e*^ In both of these transformations an organosuperbase was demonstrated to be essential for reactivity.

We speculated that the reluctance of unactivated β-substituted-α,β-unsaturated esters to undergo organocatalytic Michael addition reactions could be overcome using our BIMP catalyst family. To exemplify this we chose the sulfa-Michael addition (SMA) of alkyl thiols as this is a reaction of central importance for the asymmetric construction of chiral sulfides possessing a stereogenic centre at the β-carbon and no organocatalytic enantioselective version has previously been reported.^14,15^ We reasoned that the high Brønsted basicity of our BIMP catalysts could activate the high p*K*_a_ alkyl thiol pro-nucleophile (p*K*_a(DMSO)_ = 17 for *n*-BuSH)^16,17^ and the modular design of the catalyst family, through its variable backbone scaffold, hydrogen-bond donor group and iminophosphorane superbase would expedite optimal catalyst identification. Herein, and as part of our research program towards the development of novel asymmetric reactions with challenging electrophile/pro-nucleophile combinations, we wish to report our investigations leading to the highly enantioselective SMA reaction of alkyl thiols to unactivated β-substituted-α,β-unsaturated esters.

We chose commercially available methyl crotonate (**2a**) and 1-propanethiol (**3a**) as our model system and investigated reactivity using first generation BIMP catalyst **1a** (Table 1, entry 1). In toluene, at room temperature using 10 mol% catalyst we were delighted to observe an exceptional reactivity profile; β-mercaptoester product **4a** was afforded in near quantitative yield after only 2 hours with low but significant enantiocontrol (55 : 45 er).^18^ With good reactivity established we next investigated the performance of a small library of second generation BIMP catalysts featuring variations around the amide–thiourea motif that we recently reported^12*e*^ (Table 1, entries 2–6). The modular design of our BIMP catalysts allowed rapid library generation and our attention focussed on the amide–thiourea moiety as the H-bond donor group and the tris-(4-methoxyphenylphosphine) derived iminophosphorane as the Brønsted basic group (Fig. 2).

**Fig. 2:**
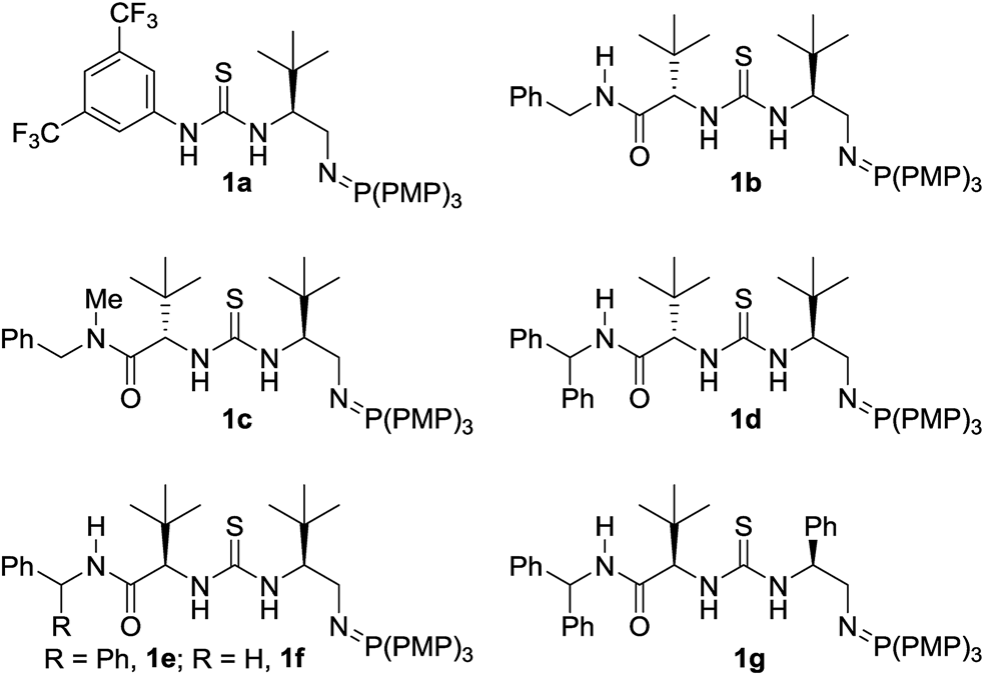
Bifunctional iminophosphorane (BIMP) organocatalysts used in the optimization of the SMA reaction. PMP = *p*-methoxyphenyl.

**Table 1:** Catalyst screening studies and reaction optimization^*a*^

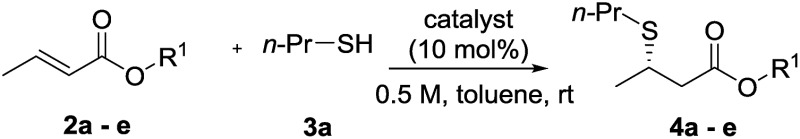						
Entry	Cat.	R^1^	Product	Time (h)	Yield^*b*^ (%)	er^*c*^
1	**1a**	Me	**4a**	2	94	55 : 45
2	**1b**	Me	**4a**	2	98	55 : 45
3	**1c**	Me	**4a**	2	94	52 : 48
4	**1d**	Me	**4a**	2	93	59 : 41
5	**1e**	Me	**4a**	2	>99	75 : 25
6	**1f**	Me	**4a**	2	97	62 : 38
7^*d*^	**1g**	Me	**4a**	3	>99	81 : 19
8	**1g**	Et	**4b**	3	95	84 : 16
9	**1g**	i-Pr	**4c**	3	>99	85 : 15
10	**1g**	Bn	**4d**	3	>99	81 : 19
11^*d*^	**1g**	*t*-Bu	**4e**	8	94	92 : 8
12^*d*^ ^,^ ^*e*^	**1g**	*t*-Bu	**4e**	8	95	94 : 6
13^*f*^	**1g**	*t*-Bu	**4e**	24	94	96 : 4
14^*g*^	**1g**	*t*-Bu	**4e**	72	94	97 : 3

^*a*^Reactions were carried out with 0.20 mmol of **2** and 0.60 mmol of **3a**.

^*b*^Isolated yield.

^*c*^Determined by HPLC analysis on a chiral stationary phase.

^*d*^Reaction performed on 0.10 mmol scale of **2a**.

^*e*^Reaction performed at 0 °C.

^*f*^Reaction performed at 0 °C in Et_2_O.

^*g*^Reaction performed at –15 °C in Et_2_O.

Catalysts **1b–d** possessing a thiourea constructed from two (*S*)-configured *tert*-leucine derived residues, the tris-(4-methoxyphenylphosphine)-derived iminophosphorane and a variable terminal amide group gave poor enantioselectivity in all cases (Table 1, entries 2, 3, and 4). When catalyst **1e** – the diastereomer of **1d** – was trialled however, a significant boost to the enantioselectivity was witnessed (Table 1, entry 5, 75 : 25 er).^19^

A comparison with an analogous catalyst possessing a phenylglycine and a *tert*-leucine residue (**1g**) resulted in a slight improvement to the enantioselectivity (Table 1, entry 7, 81 : 19 er). At this stage, the effect of varying the ester group of the crotonate on the enantioselectivity in the SMA was investigated. A range of simple, commercial or readily synthesized alkyl crotonate esters were trialled and a correlation between the size of the ester group and the enantioselectivity was observed – pleasingly *tert*-butyl crotonate (**2e**) afforded the product **4e** in 92 : 8 er albeit in a slightly increased reaction time of 8 h (Table 1, entry 11). A reoptimization of the reaction conditions to 0.5 M in Et_2_O at 0 °C (Table 1, entries 12 & 13 and ESI†) resulted in a significant boost to the enantioselectivity (96 : 4 er) and cooling the reaction temperature further to –15 °C afforded β-mercaptoester **4e** in 94% yield and 97 : 3 er (Table 1, entry 14).

With optimized reaction conditions established, the scope of the transformation with respect to the thiol pro-nucleophile and the α,β-unsaturated ester was investigated (Fig. 3). Minimal variation to the enantioselectivity was observed across a good range of linear (propyl to decyl) or branched (cyclic and acyclic) alkyl mercaptans. The reaction with 4-methoxybenzyl mercaptan was also well-tolerated and afforded the β-mercaptoester **4m** in 99% yield and 95 : 5 er at –15 °C.

**Fig. 3:**
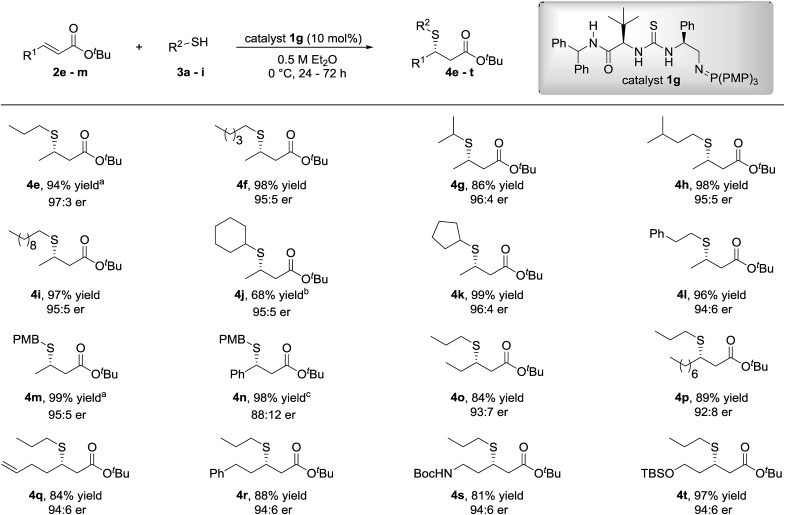
Scope of the SMA of alkyl thiols to β-substituted-α,β-unsaturated esters. Reactions were carried out with 0.20 mmol **2** and 0.60 mmol **3**. Yields are isolated yields and enantiomeric ratios were determined by HPLC analysis or GC analysis on a chiral stationary phase. ^*a*^The reaction was performed at –15 °C. ^*b*^The reaction was quenched after 96 h. ^*c*^Absolute configuration of **4n** determined by chemical correlation (see ESI†).

Following investigation into the scope of the reaction with respect to the alkyl thiol, variation to the β-substituent of the α,β-unsaturated ester was subsequently examined using 1-propanethiol or 4-methoxybenzyl mercaptan as the sulfur-centred pro-nucleophile. We were pleased to observe that the excellent reactivity and selectivities were maintained when *tert*-butyl cinnamate, bearing a phenyl group at the β-position, was used as the electrophile to afford the desired β-mercaptoester **4n** in 98% yield and 88 : 12 er.

Similarly, excellent yields of the β-mercaptoesters **4o–r** were obtained from the corresponding primary alkyl β-substituted-α,β-unsaturated esters with very good levels of enantiocontrol. β-Mercaptoesters **4s** and **4t** containing a terminal *N*-Boc protected amine and TBS protected hydroxyl group respectively were also synthesized in good to excellent yields and excellent enantiomeric ratios.

Although the scope of the reaction was performed with 10 mol% catalyst loading, we were keen to demonstrate lower loadings were viable. Accordingly, and after reoptimization of the reaction conditions, to 5 M in Et_2_O at 0 °C, we were pleased to find β-mercaptoester **4e** was afforded in near quantitative yield and 95 : 6 er on 7 mmol scale of *tert*-butyl crotonate (**2e**) using 1 mol% catalyst **1g** (Scheme 1).

**Scheme 1:**
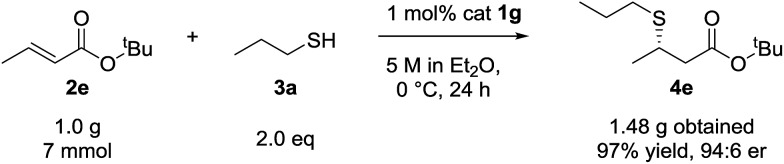
Preparative scale synthesis of **4e**.

To demonstrate synthetic utility of the β-mercaptoester products a selection of standard chemical transformations were carried out (Scheme 2). Thus β-mercaptoester **4e** (95 : 5 er) was transesterified to the methyl ester **4a** in a two step process; initial acidic cleavage of the *tert*-butyl ester and subsequent methyl ester formation under acidic conditions afforded **4a** in 78% yield without compromising stereochemical integrity. Oxidation of **4e** afforded sulfone **5a** without any observable racemization in near quantitative yield. Finally, β-mercaptoester **4m** was reduced to the alcohol in excellent yield, without appreciable loss of enantiopurity.^20^

**Scheme 2:**
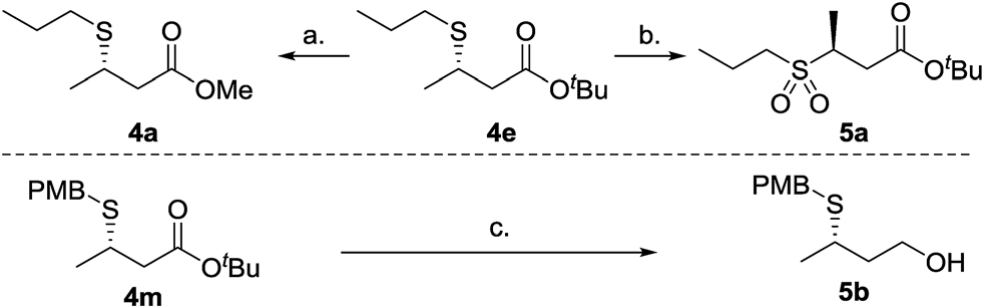
Derivatization. (a) TFA, Et_2_O, 0 °C to rt, then SOCl_2_, MeOH, 0 °C to rt, 78% yield over two steps, 94 : 6 er. (b) *m*-CPBA, CH_2_Cl_2_, 0 °C, 2 h, 96% yield, 94 : 6 er. (c) DIBAL-H, THF, –60 °C, 2 h, 93% yield, 93 : 7 er.

In summary, we have developed the first organocatalytic enantioselective SMA of alkyl thiols to unactivated β-substituted-α,β-unsaturated esters. Impressive reactivity and excellent levels of enantioselectivities were achieved across a range of linear, branched, cyclic alkyl and benzylic thiols, in SMA reactions to various β-substituted-α,β-unsaturated esters using a novel bifunctional iminophosphorane catalyst. This work demonstrates that the high reactivity of the BIMP catalysts enables low reactivity electrophiles such as β-substituted-α,β-unsaturated esters to undergo highly enantioselective conjugate addition reactions for the first time and thus represents a significant advance in the field. Work to uncover further capabilities of the BIMP catalyst family is ongoing in our laboratories and the results will be disclosed in due course.
