# Supplementary material for: Enantioselective bifunctional iminophosphorane catalyzed sulfa-Michael addition of alkyl thiols to unactivated β-substituted-α,β-unsaturated esters
Source: Chem Sci. 2016 Sep 14;8(1):606–10. doi: 10.1039/c6sc02878k (PMC5358540; doi:10.1039/c6sc02878k)
Supplement: Supplementary file 1 [file SC-008-C6SC02878K-s001.pdf]

Electronic Supplementary Material (ESI) for Chemical Science.
This journal is © The Royal Society of Chemistry 2016

Supplementary information for

Enantioselective Bifunctional Iminophosphorane Catalyzed Sulfa-Michael Addition of Alkyl Thiols to Unactivated β-Substituted-α,β-Unsaturated Esters

Jinchao Yang, Alistair J. M. Farley and Darren J. Dixon

Department of Chemistry, Chemistry Research Laboratory,

University of Oxford, 12 Mansfield Road,

Oxford, OX1 3TA, UK

1 Supplementary Methods 2

1.1 General Information 2

1.2 Synthesis and characterization of catalysts and precursors 3

1.3 Synthesis of β-Substituted α,β-Unsaturated Esters 2 7

1.4 Optimisation of conditions for the Sulfa-Michael addition 9

1.5 Synthesis and Characterisation of β-Substituted, β-Mercaptoesters 4 10

1.6 Preparative scale SMA reaction with 1 mol% catalyst 20

1.7 Derivatisation of β-Mercaptoester products 21

1.8 Determination of Absolute Stereochemistry 23

2 References 24

3 Supplementary Data 25

3.1 Copies of NMR spectra 25

3.2 Copies of HPLC and GC spectra 51

1 Supplementary Methods

1.1 General Information

Solvents and Reagents

Concentration under reduced pressure was performed by rotary evaporation at the appropriate pressure and temperature. Reagents used were obtained from commercial suppliers or purified according to standard procedures. Petroleum ether refers to distilled light petroleum of fraction 30 - 40 °C. Anhydrous toluene, tetrahydrofuran, dichloromethane and diethyl ether were dried by filtration through activated alumina (powder ~150 mesh, pore size 58 Å, basic, Sigma-Aldrich) columns. Dimethyl sulfoxide and dimethylformamide were used as supplied. Deuterated solvents were used as supplied.

Chromatography

Reactions were monitored by thin layer chromatography (TLC) using Merck silica gel 60 F_{254} plates and visualized by fluorescence quenching under UV light. In addition, TLC plates were stained with potassium permanganate solution. Flash column chromatography (FCC) was performed on VWR 60 silica gel 40 - 63 μm using technical grade solvents that were used as supplied.

Instrumentation

Melting points were obtained on a Leica Galen III Hot-stage melting point apparatus and microscope and on a Kofler hot block and are reported uncorrected. NMR spectra were recorded on a Bruker Spectrospin spectrometer operating at 200, 400 or 500 MHz (^{1}H acquisitions), 100 or 125 MHz (^{13}C acquisitions), 377 MHz (^{19}F acquisitions) and 162 MHz (^{31}P acquisitions). Chemical shifts (δ) are reported in ppm with the solvent resonance as the internal standard (e.g. Chloroform δ 7.27 ppm for ^{1}H and 77.0 ppm for ^{13}C). Coupling constants (*J*) are reported in hertz (Hz), and rounded to the nearest 0.5 Hz. Data are reported as follows: multiplicity [s = singlet, d = doublet, t = triplet, q = quartet, dd = doublet of doublets, ddd = doublet of doublets of doublets, td = triplet of doublets, m = multiplet, br = broad], coupling constants in Hz, integration. Two-dimensional spectroscopy (COSY, HSQC

and HMBC) was used to assist in the assignment and the data is not reported. High-resolution mass spectra (ESI) were recorded on Bruker µTOF mass spectrometer. Infrared spectra were recorded on a Bruker Tensor 27 FT-IR spectrometer as a thin film. Only selected maximum absorbances are reported. Optical rotations were recorded using a Perkin Elmer 341 polarimeter; $[\alpha]_D^T$ values are reported in 10^{-1} deg cm^2 g^{-1}; concentrations (c) are quoted in g/100 mL; D refers to the D-line of sodium (589 nm); temperatures (T) are given in degrees Celsius (°C). (+) and (–) compound number prefixes indicate the sign of the optical rotation. The enantiomeric excesses were determined by HPLC analysis on an Agilent 1200 Series instrument employing a chiral stationary phase column specified in the individual experiment and by comparing the samples with the appropriate racemic mixtures.

1.2 Synthesis and characterization of catalysts and precursors

Catalysts **1a**[1] and **1b-d**[2] were prepared according to literature procedures.

1.2.1 Synthesis of Isothiocyanate Precursors to Catalysts 1b – g

General Procedure A **for the synthesis of isothiocyanate precursors**

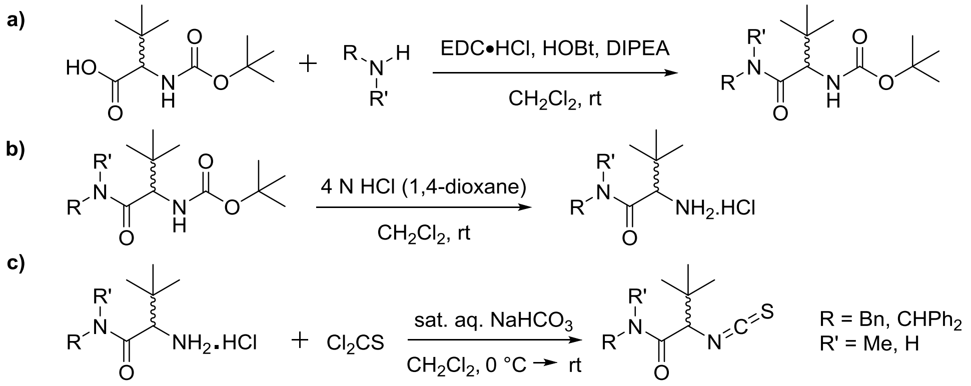

According to a literature procedure,[3]

a) To a stirred solution of EDC hydrochloride (295 mg, 1.54 mmol, 1.10 eq) and 1-hydroxybenzotriazole hydrate (208 mg, 1.54 mmol, 1.10 eq) in CH_2Cl_2 (10 mL) under a N_2 atmosphere at rt was added *N*,*N*-diisopropylethylamine (0.29 mL, 2.1 mmol, 1.5 eq) and the corresponding *amine* (1.54 mmol, 1.10 eq) sequentially. Boc-L-*tert* leucine (324 mg, 1.4 mmol, 1.00 eq) was added in one portion and the reaction mixture was stirred for 20 h. The reaction was diluted with Et_2O (10 mL), washed with 0.5 N HCl (2 x 10 mL) and the aqueous phase extracted with Et_2O (5 mL). The combined organic was washed with sat. aq. $NaHCO_3$

(10 mL) and brine (10 mL), dried (Na_2SO_4), filtered and concentrated *in vacuo* to afford the product, which was used crude without further purification.

b) To a vigorously stirred solution of the crude product in CH_2Cl_2 (5.0 mL) under a N_2 atmosphere at 0 °C was added 4 N HCl in 1,4-dioxane (3.4 mL, 13.4 mmol, 9.6 eq) over 15 min. The reaction mixture was stirred for 3.5 h and concentrated *in vacuo* to afford the product which was used crude without further purification.

c) To a vigorously stirred solution of the crude product in CH_2Cl_2 (20 mL) under a N_2 atmosphere at 0 °C was added sat. aq. $NaHCO_3$ (20 mL), and the biphasic mixture was stirred for 20 min. Stirring was stopped and thiophosgene (160 µL, 2.10 mmol, 1.5 eq) was added to the organic layer. Immediately, vigorous stirring was restored and the mixture allowed to warm to rt over 30 min. The organic phase was extracted with CH_2Cl_2 (2 x 10 mL), washed with brine (10 mL), dried (Na_2SO_4), filtered and concentrated *in vacuo* to afford the crude product.

(2*R*)-*N*-(Diphenylmethyl)-2-isothiocyanato-3,3-dimethylbutanamide 6

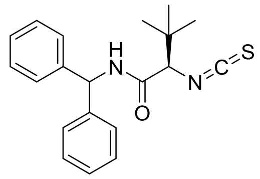

N-Boc-D-*tert*-leucine (323 mg, 1.40 mmol, 1.00 eq) was reacted with benzhydrylamine (282 µL, 1.54 mmol, 1.10 eq) according to General Procedure A to afford the title compound **6** as a pale yellow amorphous solid in 89% yield (421 mg).

$[\alpha]_D^{20}$ = -40.5 (*c* 1.00, $CHCl_3$), lit $[\alpha]_D^{20}$ = +47.5 (*c* 0.72, $CHCl_3$) for (*S*)-**6**;[2] all other characterisation data are in the accordance to that published in the literature.[2]

1.2.2 Synthesis of Azide Precursors to Catalysts 1b-g

N-Boc amino azides **7** and *ent*-**8** were synthesised according to literature procedures.[1]

General Procedure B **for the synthesis of azide precursors to catalysts 1b-1g**

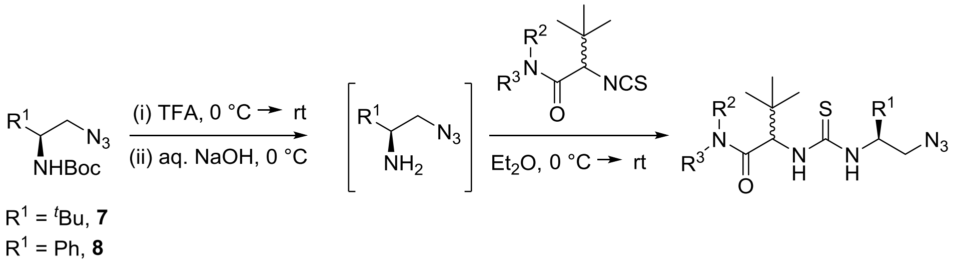

To the *N*-Boc protected amino azide **7** or **8** (0.77 mmol, 1.0 eq) at 0 °C was added trifluoroacetic acid (0.50 mL, 6.5 mmol) dropwise. The reaction mixture was warmed to rt and stirred for 2 h, and the volatiles removed by N_2 stream. The crude material was dissolved in Et_2O/H_2O (1:1 v/v, 8 mL) and adjusted to pH 14 by the addition of sodium hydroxide at 0 °C. The organic phase was extracted using Et_2O (2 x 5 mL), washed with brine (5 mL), dried ($MgSO_4$), filtered, and concentrated to 4 mL under a N_2 stream. The solution was cooled to 0 °C and the corresponding *isothiocyanate* (0.70 mmol, 1.0 eq) added. The reaction mixture was then warmed to rt and stirred for 24 h. Volatiles were removed by N_2 stream and the crude product was purified by FCC to afford the corresponding azide.

(2*R*)-2-({[(2*S*)-1-Azido-3,3-dimethylbutan-2-yl]carbamothioyl}amino)-*N*-(diphenylmethyl)-3,3-dimethylbutanamide 9

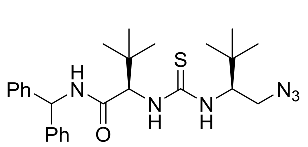

Azide **7** (185 mg, 0.77 mmol, 1.10 eq) was reacted with isothiocyanate **6** (237 mg, 0.70 mmol, 1.00 eq) according to General Procedure B. The reaction mixture was purified by FCC (petroleum ether/EtOAc = 4/1 to 3/2) to afford the title compound **9** as a colourless solid in 60% yield (202 mg).

M.P. = 227-230 °C; $[\alpha]_D^{23}$ = +4.1 (*c* 1.00, $CHCl_3$); **^{1}H NMR** (MeOD-d_4, 400 MHz) δ (ppm): 8.76 (d, *J* = 8.0 Hz, 1 H), 7.42 - 7.04 (m, 10 H), 6.19 (d, *J* = 8.0 Hz, 1 H), 5.01 (s, 1 H), 4.66 (m, 1 H), 3.55 (dd, *J* = 13.0, 4.0 Hz, 1 H), 3.32 (dd, *J* = 13.0, 8.0 Hz, 1 H), 1.01 (s, 18 H); **^{13}C NMR** (MeOD-d_4, 125 MHz) δ (ppm): 186.2, 172.7, 143.2, 142.9, 129.7, 129.5, 129.4, 128.8, 128.6, 128.3, 66.6, 63.1, 58.4, 53.1, 36.1, 35.8, 27.5, 27.4; **HRMS** (ESI+) exact mass

calculated for $[M+Na]^+$ ($C_{26}H_{36}N_6NaOS$) requires *m/z* 503.2564, found *m/z* 503.2556; **IR** (film) ν_{max}/cm^{-1}: 3304 (thiourea/amide NH), 2960, 2099, 1637 (C=O), 1533 (C=S), 1222, 1100, 756, 698.

(2*R*)-2-({[(2*S*)-1-azido-3,3-dimethylbutan-2-yl]carbamothioyl}amino)-*N*-benzyl- 3,3-dimethylbutanamide 10

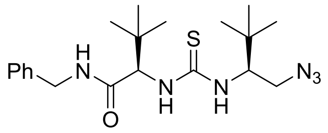

Azide **7** (185 mg, 0.77 mmol, 1.10 eq) was reacted with (2*R*)-*N*-benzyl-2-isothiocyanato-*N*,3,3-trimethylbutanamide[2] (183 mg, 0.70 mmol, 1.00 eq) according to General Procedure B. The reaction mixture was purified by FCC (petroleum ether/EtOAc = 4/1 to 3/2) to afford the title compound **10** as an off white solid in 50% yield (154 mg).

M.P. = 164 - 166 °C; $[\alpha]_D^{23}$ = -26.0 (*c 1.0,* $CHCl_3$); **^{1}H NMR** (MeOD-d_4, 400 MHz) δ (ppm): 8.39 (br. s, 1 H), 7.31 - 7.18 (m, 5 H), 4.95 (s, 1 H), 4.65 – 4.57 (m, 1 H), 4.40 - 4.30 (m, 2 H), 3.50 (dd, *J* = 12.5, 4.0 Hz, 1 H), 3.34 – 3.26 (m, 1 H), 1.00 (s, 9 H), 0.96 (s, 9 H); **^{13}C NMR** (100 MHz, MeOD-d_4) δ (ppm): 184.5, 171.7, 138.4, 128.0, 127.4, 126.7, 65.3, 61.5, 51.6, 42.6, 34.5, 34.3, 26.0, 25.8; **HRMS** (EI+) exact mass calculated for $[M+Na]^+$ ($C_{20}H_{32}N_6NaOS$) requires *m/z* 427.2258, found *m/z* 427.2234; **IR** (film) ν_{max}/cm^{-1}: 3272, 2963, 2095, 1644, 1532, 697.

(2*R*)-2-({[(1*S*)-2-Azido-1-phenylethyl]carbamothioyl}amino)-*N*-(diphenylmethyl)-3,3-dimethylbutanamide 11

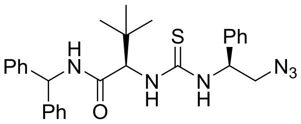

Azide **8** (403 mg, 1.54 mmol, 1.10 eq) was reacted with isothiocyanate **6** (237 mg, 1.40 mmol, 1.00 eq) according to General Procedure B. The reaction mixture was purified by FCC (petroleum ether/EtOAc = 4/1 to 3/2) to afford the title compound **11** as a colourless solid in 60% yield (420 mg).

M.P. = 188-190 °C; $[\alpha]_D^{23}$ = +23.4 (*c* = 1.02, $CHCl_3$); **^{1}H NMR** (MeOD-d_4, 400 MHz) δ (ppm): 8.87 (d, *J* = 8.5 Hz, 1H), 7.43 - 7.20 (m, 15 H), 6.22 (d, *J* = 8.5 Hz, 1 H), 5.73 (t, *J* = 6.0 Hz, 1 H), 4.95 (s, 1 H), 3.74 (dd, *J* = 12.5, 6.0 Hz, 1 H), 3.66 (dd, *J* = 12.5, 6.0 Hz, 1 H), 0.98 (s, 9 H); **^{13}C NMR** (MeOD-d_4, 100 MHz) δ (ppm): 181.6, 171.6, 139.9, 139.0, 138.0,

128.7, 128.5, 128.3, 128.3, 127.8, 127.7, 127.5, 127.1, 127.0, 65.4, 57.6, 57.3, 55.4, 34.7, 26.3; **HRMS** (ES+) exact mass calculated for $[M+Na]^+$ ($C_{26}H_{36}N_6NaOS$) requires *m/z* 523.2358, found *m/z* 523.2237; **IR** (film) ν_{max}/cm^{-1}: 3236 (thiourea/amide NH), 2970, 2107, 1666 (C=O), 1532 (C=S), 1508, 1339, 1279, 1100, 751, 699, 644, 609.

General Procedure C **for the *in situ* Generation of Iminophosphorane Catalysts**

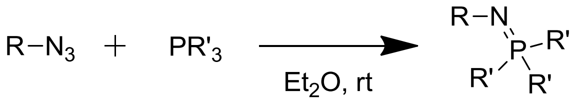

To the corresponding *organoazide* (0.020 mmol, 1.0 eq) and *phosphine* (0.020 mmol, 1.0 eq) under an Ar atmosphere was added Et_2O (0.1 mL), and the reaction mixture was stirred at rt for 24 h. The iminophosphorane product was confirmed by LRMS and TLC, and the volatiles were removed by a N_2 stream to yield the crude product, which was used as a catalyst without further purification.

1.3 Synthesis of β-Substituted α,β-Unsaturated Esters 2

Crotonic esters **2a** – **e** are commercially available and were used as supplied.

General Procedure D **for the synthesis of β-substituted α,β-unsaturated esters 2g - m**

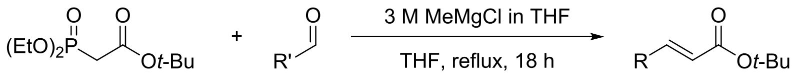

MeMgCl (3 M in THF, 1.67 mL, 5.0 mmol) was added dropwise to a stirred solution *tert*-butyl diethylphosphonoacetate (1.15 mL, 5.00 mmol) in THF (10 mL) at rt and stirred for 15 min. A solution of the *aldehyde* (5.0 mmol, 1.0 eq) in THF (5 mL) was then added *via* cannula and the reaction mixture heated at reflux for 18 h whereupon the reaction mixture was cooled to rt, quenched with sat aq NH_4Cl (10 mL) and extracted with Et_2O (3 × 10 mL). The combined organic layers were then washed with brine (15 mL), dried and concentrated *in vacuo*. The crude products were purified by flash column chromatography (Petroleum ether : Et_2O mixtures) to afford the desired β-substituted α-,β-unsaturated esters.

β-Substituted α-,β-unsaturated esters **2f**,[4] **2h**,[6] **2i**,[7] **2j**,[8] have been synthesised and characterised previously in the literature. Their physical and spectroscopic properties are in agreement to those reported.

***tert*-Butyl (*E*)-pent-2-enoate 2g**

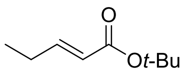

Synthesised on a 5.0 mmol scale according to General Procedure **D** using *tert*-butyl diethylphosphonoacetate (1.15 mL, 5.0 mmol) and propionaldehyde (0.39 mL, 5.5 mmol) to afford the title compound **2g** as a colourless oil (720 mg, 92% yield). All spectroscopic data are consistent to that published in the literature.[5]

***tert*-Butyl (*E*)-5-((*tert*-butoxycarbonyl)amino)pent-2-enoate 2k**

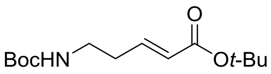

Synthesised on a 3.75 mmol scale according to General Procedure **D** using *tert*-butyl diethylphosphonoacetate (0.87 mL, 3.75 mmol) and *tert*-butyl (3-oxopropyl) carbamate[9] (645 mg, 3.75 mmol) to afford the title compound **2k** as a colourless oil (500 mg, 49% yield). All spectroscopic data are consistent to that reported in the literature.[10]

***tert*-Butyl (*E*)-5-((*tert*-butyldimethylsilyl)oxy)pent-2-enoate 2l**

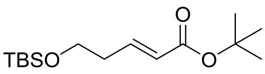

Synthesised on a 3.75 mmol scale according to General Procedure **D** using *tert*-butyl diethylphosphonoacetate (1.04 mL, 4.50 mmol) and 3-((*tert*-butyldimethylsilyl)oxy)propanal[11] (846 mg, 4.50 mmol) to afford the title compound **2l** as a colourless oil (660 mg, 51% yield). All spectroscopic data are consistent to that reported in the literature.[12]

1.4 Optimisation of Conditions for the Sulfa-Michael Addition

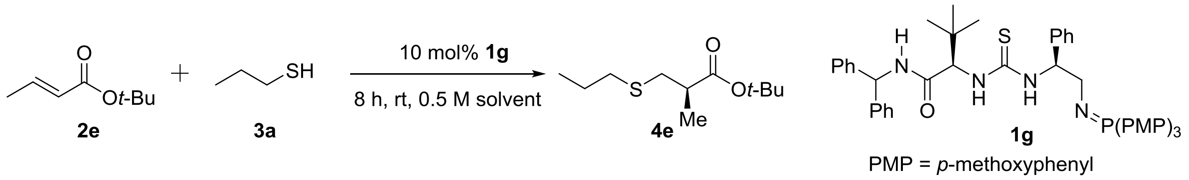

Entry	Solvent	Yield / %[a]	er[b]
1	Toluene	94	92:8
2	TBME	92	92:8
3	Xylene	91	92:8
4	THF	89	92:8
5	EtOAc	94	92:8
6	$CHCl_3$	23	84:16
7	CH_2Cl_2	Trace	-
8	Hexane	92	88:12
9	DMF	96	52:48
10	Et_2O	95	94:6

Table SI.1: Solvent optimization for the sulfa-Michael addition of 1-propanethiol **3a** (0.30 mmol) to *tert*-butyl crotonate **2e** (0.10 mmol), with 10 mol% catalyst **1g**. [a] Isolated yield. [b] Determined by HPLC analysis on a chiral stationary phase.

Entry	Temperature / °C	Time /h	Yield / %[a]	er[b]
1	RT	8	95	94:6
2[c]	0	24	94	96:4
3[c]	-15	72	94	97:3

Table SI.2: Temperature optimization for the sulfa-Michael addition of 1-propanethiol **3a** (0.30 mmol) to *tert*-butyl crotonate **2e** (0.10 mmol), with 10 mol% catalyst **1g** in 0.5 M Et_2O. [a] Isolated yield. [b] Determined by HPLC analysis on a chiral stationary phase. [c] Reaction performed with 0.20 mmol **2e**.

1.5 Synthesis and Characterisation of β-Substituted, β-Mercaptoesters 4

General Procedure E **for the Enantioselective Sulfa-Michael Addition of Alkyl Thiols to β-Substituted α-,β-Unsaturated Esters 4**

Azide **11** (10.0 mg, 0.020 mmol, 0.10 eq) and tris(4-methoxyphenyl)phosphine (7.0 mg, 0.020 mmol, 0.10 eq) were stirred in diethyl ether (0.2 mL) under an argon atmosphere in a sealed vial at room temperature for 24 h. Formation of the *in situ* generated catalyst was confirmed by LRMS and TLC analysis and the volatiles were removed under a N_2 stream. To the crude catalyst was added sequentially Et_2O (0.40 mL), the desired *β-substituted α-,β-unsaturated ester* **2** (0.20 mmol, 1.0 eq) under an Ar atmosphere. The reaction mixture was cooled to 0 °C, then the desired *thiol* **3** (0.60 mmol, 3.0 eq) was added and stirring was maintained for 24 h (or the time specified in the individual experiment as determined by TLC analysis). The reaction mixture was quenched by the addition of 1.0 M AcOH (in CH_2Cl_2, 0.1 mL), the volatiles were removed under a stream of N_2 and purification by flash column chromatography (petroleum ether / Et_2O mixtures) afforded the β-mercaptoesters **4**.[a]

Methyl (*S*)-3-(propylthio)butanoate 4a

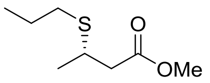

To a solution of methyl crotonate **2a** (21 μL, 0.20 mmol, 1.0 eq) and **1g** (0.020 mmol, 0.10 eq) in toluene (0.40 mL) at RT was added 1-propanethiol **3a** (54 μL, 0.60 mmol, 3.0 eq) according a modified General Procedure **E**. The product was purified by FCC (99:1 petrol: Et_2O) to afford **4a** as a colourless oil in >99% yield (35.2 mg) and 81:19 er [determined by HPLC, chiralpak IA, hexane/isopropanol 99.5:0.5, λ 220 nm, 1.0mL/min, t (major) = 12.67 min, t (minor) = 18.01 min].

$[\alpha]_D^{23}$ = +4.8 (*c 1.00,* $CHCl_3$); **^{1}H NMR** ($CDCl_3$, 400 MHz) δ (ppm): 3.70 (s, 3 H), 3.24-3.16 (m, 1 H), 2.64 (dd, *J* = 15.5, 6.5 Hz, 1 H), 2.53 (t, *J* = 7.5 Hz, 2 H), 2.46 (dd, *J* = 15.5, 8.5 Hz, 1 H), 1.62 (sxt, *J* = 7.5 Hz, 2 H), 1.33 (d, *J* = 6.5 Hz, 3 H), 1.26 (t, *J* = 7.5 Hz, 3 H); **^{13}C NMR** ($CDCl_3$, 100 MHz) δ (ppm): 172.0, 51.7, 42.2, 36.1, 32.7, 23.0, 21.5, 13.6; **HRMS** (ESI+) mass calculated for $[M+Na]^+$ ($C_8H_{16}NaO_2S$) requires *m/z* 199.0763, found *m/z* 199.0757; **IR** (film) ν_{max}/cm^{-1}: 2963, 1738, 1437, 1222, 1161, 913, 734.

[a] Racemic products for HPLC/GC analysis were formed using a modification of General Procedure **E**, with 10 mol% BEMP (2-*tert*-butylimino-2-diethylamino-1,3-dimethylperhydro-1,3,2-diazaphosphorine) in 0.5 M toluene, and left until completion.

Ethyl (*S*)-3-(propylthio)butanoate 4b

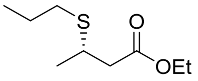

To a solution of ethyl crotonate **2b** (25 μL, 0.20 mmol, 1.0 eq) and **1g** (0.020 mmol, 0.10 eq) in toluene (0.40 mL) at RT was added 1-propanethiol **3a** (54 μL, 0.60 mmol, 3.0 eq) according to modified General Procedure **E**. The product was purified by FCC (99:1 petrol: Et_2O) to afford **4b** as a colourless oil in 95% yield (36.1 mg) and 84:16 er [determined by HPLC, chiralpak IA, hexane/isopropanol 100:0, λ 220 nm, 1.0 mL/min, t (minor) = 16.56 min, t (major) = 18.42 min].

$[\alpha]_D^{23}$ = +4.0 (*c 0.60*, $CHCl_3$); **^{1}H NMR** ($CDCl_3$, 400 MHz) δ (ppm): 4.15 (q, *J* = 7.0 Hz, 2 H), 3.20 - 3.13 (m, 1 H), 2.61 (dd, *J* = 15.5, 6.0 Hz, 1 H), 2.55 - 2.48 (m, 2 H), 2.42 (dd, *J* = 15.5, 8.5 Hz, 1 H), 1.60 (sxt, *J* = 7.5 Hz, 2 H), 1.31 (d, *J* = 7.0 Hz, 3 H), 1.26 (d, *J* = 14.0 Hz, 3 H), 0.96 (t, *J* = 7.0 Hz, 3 H); **^{13}C NMR** ($CDCl_3$, 400 MHz) δ (ppm): 171.6, 60.5, 42.4, 36.1, 32.7, 23.0, 21.4, 14.2, 13.5; **HRMS** (ESI+) mass calculated for $[M+Na]^+$ ($C_9H_{18}NaO_2S$) requires *m/z* 213.0920, found *m/z* 213.0922; **IR** (film) ν_{max}/cm^{-1}: 2968, 1729, 1216, 1169, 754, 667.

Isopropyl (*S*)-3-(propylthio)butanoate 4c

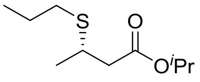

To a solution of isopropyl crotonate **2c** (25.6 mg, 0.20 mmol, 1.0 eq) and **1g** (0.020 mmol, 0.10 eq) in toluene (0.40 mL) at RT was added 1-propanethiol **3a** (54 μL, 0.60 mmol, 3.0 eq) according to modified General Procedure **E**. The product was purified by FCC (99:1 petrol: Et_2O) to afford **4c** as a colourless oil in >99% yield (41 mg) and 85:15 er [determined by HPLC, chiralpak AD-H, hexane/isopropanol 99:1, λ 220 nm, 1.0 mL/min, t (major) = 7.45 min, t (minor) = 7.81 min].

$[\alpha]_D^{23}$ = +1.6 (*c 0.45*, $CHCl_3$); **^{1}H NMR** ($CDCl_3$, 400 MHz) δ (ppm): 5.04 (spt, *J* = 6.5 Hz, 1 H), 3.19 (dquin, *J* = 8.0, 6.5 Hz, 1 H), 2.59 (dd, *J* = 15.0, 6.5 Hz, 1 H), 2.53 (t, *J* = 7.5 Hz, 2 H), 2.40 (dd, *J* = 15.0, 8.5 Hz, 1 H), 1.61 (sxt, *J* = 7.5 Hz, 2 H), 1.32 (d, *J* = 6.5 Hz, 3 H), 1.25 (d, *J* = 6.5 Hz, 6 H), 0.99 (t, *J* = 7.5 Hz, 3 H); **^{13}C NMR** ($CDCl_3$, 100 MHz) δ (ppm): 171.1, 67.9, 42.7, 36.2, 32.6, 23.0, 21.8, 21.4, 13.6; **HRMS** (ESI+) mass calculated for $[M+Na]^+$ ($C_{10}H_{20}NaO_2S$) requires *m/z* 227.1076, found *m/z* 227.1084; **IR** (film) ν_{max}/cm^{-1}: 2926, 1722, 1216, 1106, 755.

Benzyl (*S*)-3-(propylthio)butanoate 4d

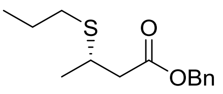

To a solution of benzyl crotonate **2d** (35.2 mg, 0.20 mmol, 1.0 eq) and **1g** (0.020 mmol, 0.10 eq) in toluene (0.40 mL) at RT was added 1-propanethiol **3a** (54 μL, 0.60 mmol, 3.0 eq) according to modified General Procedure **E**. The product was purified by FCC (99:1 petrol: Et_2O) to afford **4d** as a colourless oil in >99% yield (50.3 mg) and 81:19 er [determined by HPLC, chiralpak AD-H, hexane/isopropanol 99:1, λ 220 nm, 1.0 mL/min, t (major) = 13.94 min, t (minor) = 15.87 min].

$[\alpha]_D^{23}$ = 0 (c *0.62*, $CHCl_3$); **^{1}H NMR** ($CDCl_3$, 400 MHz) δ (ppm): 7.38-7.34 (m, 5 H), 5.05 (s, 2 H), 3.13 - 3.10 (m, 1 H), 2.62 (dd, *J* = 15.5, 6.5 Hz, 1 H), 2.44-2.40 (m, 3 H), 1.50 (sxt, *J* = 7.5 Hz, 2 H), 1.23 (d, *J* = 7.0 Hz, 3 H), 0.88 (t, *J* = 7.5 Hz, 3 H); **^{13}C NMR** ($CDCl_3$, 100 MHz) δ (ppm): 171.4, 135.8, 128.6, 128.3[b], 66.4, 42.4, 36.1, 32.7, 23.0, 21.5, 15.3; **HRMS** (ESI+) mass calculated for $[M+Na]^+$ ($C_{14}H_{20}NaO_2S$) requires *m/z* 275.1076, found *m/z* 275.1079; IR (film) ν_{max}/cm^{-1}: 2962, 1731, 1216, 1156, 753, 697, 668.

***tert*-Butyl (*S*)-3-(propylthio)butanoate 4e**

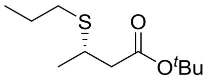

To a solution of *tert*-butyl crotonate **2e** (32 μL, 0.20 mmol, 1.0 eq) and **1g** (0.020 mmol, 0.10 eq) in Et_2O (0.40 mL) at -15 °C was added 1-propanethiol **3a** (54 μL, 0.60 mmol, 3.0 eq) and stirring was maintained for 72 h according a modified modified General Procedure **E**. The product was purified by FCC (99:1 petrol: Et_2O) to afford **4e** as a colourless oil in 94% yield (41 mg) and 97:3 er [determined by HPLC, chiralpak IA, hexane/isopropanol 100:0, λ 220 nm, 1.0 mL/min, t (major) = 9.45 min, t (minor) = 11.34 min].

$[\alpha]_D^{23}$ = +5.5 (c *1.85*, $CHCl_3$); **^{1}H NMR** ($CDCl_3$, 400 MHz) δ (ppm): 3.22 – 3.10 (m, 1 H), 2.57-2.51 (m, 3 H), 2.34 (dd, *J* = 15.0, 8.5 Hz, 1 H), 1.61 (sxt, *J* = 7.0 Hz, 2 H), 1.46 (s, 9 H), 1.32 (d, *J* = 6.5 Hz, 3 H), 1.26 (t, *J* = 7.0 Hz, 3 H); **^{13}C NMR** ($CDCl_3$, 400 MHz) δ (ppm): 170.9, 80.7, 43.6, 36.3, 32.6, 28.1, 23.0, 21.3, 13.6; **HRMS** (FI+) mass calculated for $[M]^+$ ($C_{11}H_{22}O_2S$) requires *m/z* 218.1341, found *m/z* 218.1373; **IR** (film) ν_{max}/cm^{-1}: 2966, 1725, 1368, 1216, 1148, 753, 667.

[b] One of the ArCH is missing, probably due to overlap of the aromatic carbon signals.

tert-Butyl (S)-3-(pentylthio)butanoate 4f

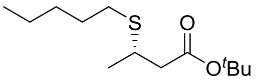

To a solution of *tert*-butyl crotonate **2e** (32 µL, 0.20 mmol, 1.0 eq) and **1g** (0.020 mmol, 0.10 eq) in Et_2O (0.40 mL) at 0 °C was added 1-pentanethiol **3b** (74 µL, 0.60 mmol, 3.00 eq) according General Procedure **E**. The reaction mixture was purified by FCC (petroleum ether: Et_2O 99:1) to afford **4f** as a colourless oil in 98% yield (48 mg) and 95:5 er [determined by HPLC, chiralpak IA hexane/isopropanol 100:0, λ 220 nm, 1.0 mL/min, t (major) = 11.09 min, t (minor) = 13.30 min].

$[\alpha]_D^{23}$ = +4.8 (*c* 1.57, $CHCl_3$); **^{1}H NMR** ($CDCl_3$, 400 MHz) δ (ppm): 3.19 - 3.10 (m, 1 H), 2.56 – 2.48 (m, 3 H), 2.32 (dd, *J* = 15.0, 8.5 Hz, 1 H), 1.63 – 1.53 (m, 2 H), 1.45 (s, 9 H), 1.39-1.29 (m, 4 H), 1.30 (d, *J* = 7.0 Hz, 3 H), 0.89 (t, *J* = 7.0 Hz, 3 H); **^{13}C NMR** ($CDCl_3$, 100 MHz) δ (ppm): 171.0, 80.8, 43.7, 36.5, 31.3, 30.7, 29.5, 28.2, 22.4, 21.5, 14.1; **HRMS** (ESI+) mass calculated for $[M+Na]^+$ ($C_{13}H_{26}O_2NaS$) requires *m/z* 269.1546, found *m/z* 269.1546; **IR** (film) ν_{max}/cm^{-1}: 2929, 1727, 1368, 1149, 907, 731, 648.

tert-Butyl (S)-3-(isopropylthio)butanoate 4g

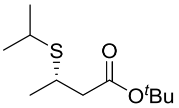

To a solution of *tert*-butyl crotonate **2e** (32 µL, 0.20 mmol, 1.0 eq) and **1g** (0.020 mmol, 0.10 eq) in Et_2O (0.40 mL) at 0 °C was added 2-propanethiol **3c** (56 µL, 0.60 mmol, 3.0 eq) according General Procedure **E**. The reaction mixture was purified by FCC (petroleum ether: Et_2O 99:1) to afford **4g** as a colourless oil in 86% yield (37.5 mg) and 96:4 er [determined by GC, Supelco β-dex™ 325, 30 m, 0.25 mm, 0.25 µm, carrier gas He (flow rate 30 cm/s); column temperature 80 ˚C ramp 1 ˚C/min to 90 ˚C then 90 ˚C, t (minor) = 60.86 min, t (major) = 61.78 min].

$[\alpha]_D^{23}$ = +1.6 (*c* 0.8, $CHCl_3$); **^{1}H NMR** ($CDCl_3$, 400 MHz) δ (ppm): 3.28 - 3.13 (m, 1 H), 2.99 (spt, *J* = 7.0 Hz, 1 H), 2.51 (dd, *J* = 15.0, 6.0 Hz, 1 H), 2.33 (dd, *J* = 15.0, 8.5 Hz, 1 H), 1.45 (s, 9 H), 1.30 (d, *J* = 7.0 Hz, 3 H), 1.26 (t, *J* = 7.0 Hz, 6 H); **^{13}C NMR** ($CDCl_3$, 100 MHz) δ (ppm): 171.1, 80.8, 43.9, 35.4, 34.2, 28.2, 23.9, 23.7, 21.9; **HRMS** (ESI+) mass calculated for $[M+Na]^+$ ($C_{11}H_{22}O_2NaS$) requires *m/z* 241.1233, found *m/z* 241.1229; **IR** (film) ν_{max}/cm^{-1}: 2972, 1727, 1368, 1149, 909, 731, 648.

***tert*-Butyl (*S*)-3-(isopentylthio)butanoate 4h**

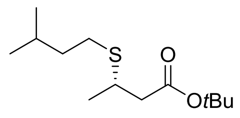

To a solution of *tert*-butyl crotonate **2e** (32 μL, 0.20 mmol, 1.0 eq) and **1g** (0.020 mmol, 0.10 eq) in Et_2O (0.40 mL) at 0 °C was added 3-methyl-1-butanethiol **3d** (74 μL, 0.60 mmol, 3.0 eq) according General Procedure **E**. The reaction mixture was purified by FCC (petroleum ether: Et_2O 99:1) to afford **4h** as a yellow oil in 98% yield (48 mg) and 95:5 er [determined by HPLC, chiralpak IA, hexane/isopropanol 100:0, λ 210 nm, 1.0 mL/min, t (major) = 10.55 min, t (minor) = 12.42 min].

$[\alpha]_D^{23}$ = -4.9 (*c* 1.5, $CHCl_3$); **^{1}H NMR** ($CDCl_3$, 400 MHz) δ (ppm): 3.19 - 3.10 (m, 1 H), 2.56 – 2.50 (m, 3 H), 2.33 (dd, *J* = 15.0, 8.5 Hz, 1 H), 1.72 – 1.59 (m, 1 H), 1.49 - 1.43 (m, 2 H), 1.45 (s, 9 H), 1.30 (d, *J* = 7.0 Hz, 3 H), 0.89 (d, *J* = 7.0 Hz, 6 H); **^{13}C NMR** ($CDCl_3$, 100 MHz) δ (ppm): 171.0, 80.8, 43.7, 38.8, 36.5, 28.7, 28.2, 27.6, 22.4, 22.4, 21.4; **HRMS** (ESI+) mass calculated for $[M+Na]^+$ ($C_{13}H_{26}O_2NaS$) requires *m/z* 269.1546, found *m/z* 269.1541; **IR** (film) ν_{max}/cm^{-1}: 2959, 1729, 1368, 1149, 909, 733, 648.

***tert*-Butyl (*S*)-3-(decylthio)butanoate 4i**

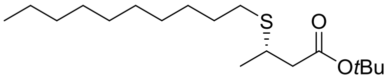

To a solution of *tert*-butyl crotonate **2e** (32 μL, 0.20 mmol, 1.0 eq) and **1g** (0.020 mmol, 0.10 eq) in Et_2O (0.40 mL) at 0 °C was added decane-1-thiol **3e** (124 μL, 0.60 mmol, 3.0 eq) according General Procedure **E**. The reaction mixture was purified by FCC (petroleum ether: Et_2O 99:1) to afford **4i** as a colourless oil in 97% yield (61 mg) and 95:5 er [determined by GC, Supelco β-dex^{TM} 325, 30 m, 0.25 mm, 0.25 μm, carrier gas He (flow rate 30 cm/s); column temperature 90 ˚C ramp 1 ˚C/min to 100 ˚C then 100 ˚C, t (minor) = 195.88 min, t (minor) = 197.92 min].

$[\alpha]_D^{23}$ = +3.5 (*c* 1.2, $CHCl_3$); **^{1}H NMR** ($CDCl_3$, 400 MHz) δ (ppm): 3.19 - 3.10 (m, 1 H), 2.56 – 2.50 (m, 3 H), 2.33 (dd, *J* = 15.0, 8.5 Hz, 1 H), 1.62 – 1.53 (m, 2 H), 1.45 (s, 9 H), 1.38 - 1.25 (m, 17 H), 0.87 (d, *J* = 7.0 Hz, 3 H); **^{13}C NMR** ($CDCl_3$, 100 MHz) δ (ppm): 171.0, 80.8, 43.7, 36.5, 32.0, 30.7, 29.9, 29.7, 29.5, 29.4, 29.2, 28.7, 28.2, 22.8, 21.5, 14.3; **HRMS** (ESI+) mass calculated for $[M+Na]^+$ ($C_{18}H_{36}O_2NaS$) requires *m/z* 339.2328, found *m/z* 339.2320; **IR** (film) ν_{max}/cm^{-1}: 2925, 2855, 1730, 1457, 1367, 1148, 908, 733, 648.

tert-Butyl (_S_)-3-(cyclohexylthio)butanoate 4j

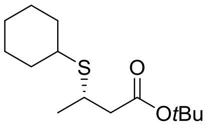

To a solution of *tert*-butyl crotonate **2e** (32 μL, 0.20 mmol, 1.0 eq) and **1g** (0.020 mmol, 0.10 eq) in Et_2O (0.40 mL) at 0 °C was added cyclohexanethiol (73 μL, 0.60 mmol, 3.0 eq) **3f** according to a modified General Procedure **E** stirring for 96 h. The reaction mixture was purified by FCC (petroleum ether: Et_2O 99:1) to afford **4j** as a colourless oil in 68% yield (34.8 mg) and 95:5 er [determined by HPLC, chiralpak IA, hexane/isopropanol 100:0, λ 220 nm, 0.5 mL/min, t (major) = 27.64 min, t (minor) = 31.64 min].

$[\alpha]_D^{23}$ = +1.1 (*c* 0.9, $CHCl_3$); **^{1}H NMR** ($CDCl_3$, 400 MHz) δ (ppm): 3.26 – 3.17 (m, 1 H), 2.75 – 2.65 (m, 1 H), 2.50 (dd, *J* = 15.0, 6.0 Hz, 1 H), 2.31 (dd, *J* = 15.0, 8.5 Hz, 1 H), 2.00 – 1.88 (m, 2 H), 1.80 – 1.70 (m, 2 H), 1.63 – 1.55 (m, 1 H), 1.44 (s, 9 H), 1.32 – 1.22 (m, 8 H); **^{13}C NMR** ($CDCl_3$, 100 MHz) δ (ppm): 171.0, 80.7, 44.1, 42.8, 35.0, 34.2, 34.1, 28.2, 26.2, 25.9, 22.1; **HRMS** (ESI+) mass calculated for $[M+Na]^+$ ($C_{14}H_{26}O_2NaS$) requires *m/z* 281.1546, found *m/z* 281.1533; **IR** (film) ν_{max}/cm^{-1}: 2928, 2853, 1728, 1367, 1145, 844, 732.

tert-Butyl (_S_)-3-(cyclopentylthio)butanoate 4k

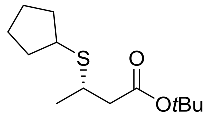

To a solution of *tert*-butyl crotonate **2e** (32 μL, 0.20 mmol, 1.0 eq) and **1g** (0.020 mmol, 0.10 eq) in Et_2O (0.40 mL) at 0 °C was added cyclopentanethiol **3g** (64 μL, 0.60 mmol, 3.0 eq) according to General Procedure **E**. The reaction mixture was purified by FCC (petroleum ether: Et_2O 99:1) to afford **4k** as a colourless oil in 99% yield (48.4 mg) and 96:4 er [determined by HPLC, chiralpak IA, hexane/isopropanol 100:0, λ 220 nm, 1.0 mL/min, t (major) = 13.15 min, t (minor) = 16.11 min].

$[\alpha]_D^{23}$ = +5.3 (*c* 1.1, $CHCl_3$); **^{1}H NMR** ($CDCl_3$, 400 MHz) δ (ppm): 3.22 – 3.09 (m, 2 H), 2.53 (dd, *J* = 15.0, 6.0 Hz, 1 H), 2.32 (dd, *J* = 15.0, 8.5 Hz, 1 H), 2.08 – 1.93 (m, 2 H), 1.78 – 1.68 (m, 2 H), 1.60 – 1.45 (m, 4 H), 1.44 (s, 9 H), 1.31 (d, *J* = 6.5 Hz, 3 H); **^{13}C NMR** ($CDCl_3$, 100 MHz) δ (ppm): 171.1, 80.8, 43.9, 42.8, 36.6, 34.4, 34.2, 28.2, 24.9, 24.8, 21.8; **HRMS** (ESI+) mass calculated for $[M+Na]^+$ ($C_{13}H_{24}O_2NaS$) requires *m/z* 267.1389, found *m/z* 267.1382; **IR** (film) ν_{max}/cm^{-1}: 2960, 1727, 1368, 1147, 907, 843, 730.

***tert*-Butyl (*S*)-3-(phenethylthio)butanoate 4l**

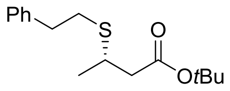

To a solution of *tert*-butyl crotonate **2e** (32 µL, 0.20 mmol, 1.0 eq) and **1g** (0.020 mmol, 0.10 eq) in Et_2O (0.40 mL) at 0 °C was added 2-phenylethanethiol **3h** (80 µL, 0.60 mmol, 3.0 eq) according to General Procedure **E**. The reaction mixture was purified by FCC (petroleum ether: Et_2O 99:1) to afford **4l** as a colourless oil in 96% yield (54 mg) and 94:6 er [determined by HPLC, chiralpak IA, hexane/isopropanol 100:0, λ 210 nm, 1.0 mL/min, t (major) = 9.23 min, t (minor) = 10.49 min].

$[\alpha]_D^{23}$ = +5.6 (*c 1.3,* $CHCl_3$); **^{1}H NMR** ($CDCl_3$, 400 MHz) δ (ppm): 7.33 – 7.27 (m, 2 H), 7.24 – 7.18 (m, 3 H), 3.26 – 3.16 (m, 1 H), 2.91 – 2.87 (m, 2 H), 2.83 – 2.79 (m, 2 H), 2.55 (dd, *J* = 15.0, 6.0 Hz, 1 H), 2.36 (dd, *J* = 15.0, 8.5 Hz, 1 H), 1.46 (s, 9 H), 1.33 (d, *J* = 6.5 Hz, 3 H); **^{13}C NMR** ($CDCl_3$, 100 MHz) δ (ppm): 170.9, 140.7, 128.6 (2 C), 126.4, 80.8, 43.6, 36.7, 36.4, 32.2, 28.2, 21.4; **HRMS** (ESI+) mass calculated for $[M+Na]^+$ ($C_{16}H_{24}O_2NaS$) requires *m/z* 303.1389, found *m/z* 303.1374. **IR** (film) ν_{max}/cm^{-1}: 2976, 1726, 1367, 1146, 843, 732, 697.

***tert*-Butyl (*S*)-3-((4-methoxybenzyl)thio)butanoate 4m**

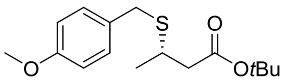

To a solution of *tert*-butyl crotonate **2e** (32 µL, 0.20 mmol, 1.0 eq) and **1g** (0.020 mmol, 0.10 eq) in Et_2O (0.40 mL) at -15 °C was added 4-methoxy-α-toluenethiol **3i** (84 µL, 0.60 mmol, 3.0 eq) and the reaction mixture was stirred at -15 °C for 24 h according to a modified General Procedure **E**. The reaction mixture was purified by FCC (petroleum ether: Et_2O 99:1) to afford **4m** as a colourless oil in >99% yield (58.8 mg) and 95:5 er [determined by HPLC, chiralpak AS-H, hexane/isopropanol 99:1, λ 220 nm, 1.0 mL/min, t (major) = 8.27 min, t (minor) = 12.35 min].

$[\alpha]_D^{23}$ = - 1.9 (*c* 1.0, $CHCl_3$); ^{1}H NMR ($CDCl_3$, 400 MHz) δ (ppm): 7.25 - 7.22 (m, 2 H), 6.86 – 6.82 (m, 2 H), 3.79 (s, 3 H), 3.72 (s, 2 H), 3.11 – 3.02 (m, 1 H), 2.53 (dd, *J* = 15.0, 6.0 Hz, 1 H), 2.33 (dd, *J* = 15.0, 8.5 Hz, 1 H), 1.44 (s, 9 H), 1.28 (d, *J* = 7.0 Hz, 3 H); **^{13}C NMR** ($CDCl_3$, 100 MHz) δ (ppm): 170.8, 158.7, 130.3, 130.0, 114.0, 80.8, 55.4, 43.4, 36.1, 34.7, 28.2, 21.2; **HRMS** (ESI+) mass calculated for $[M+Na]^+$ ($C_{16}H_{24}NaO_3S$) requires *m/z* 319.1338, found *m/z* 319.1329; **IR** (film) ν_{max}/cm^{-1}: 2976, 1725, 1511, 1247, 1147, 833, 730.

***tert*-Butyl (*R*)-3-((4-methoxybenzyl)thio)-3-phenylpropanoate 4n**

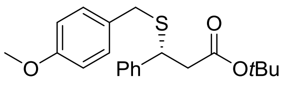

To a solution of *tert*-butylcinnamate **2f** (40.0 mg, 0.20 mmol, 1.0 eq) and **1g** (0.020 mmol, 0.10 eq) in Et_2O (0.40 mL) at 0 °C was added 4-methoxy-α-toluenethiol **3i** (84 µL, 0.60 mmol, 3.0 eq) according to a modified General Procedure **E** stirring for 72 h at 0 °C. The reaction mixture was purified by FCC (petroleum ether: Et_2O 99:1) to afford **4n** as a colourless oil in 98% yield (70.0 mg) and 88:12 er [determined by HPLC, chiralpak IA, hexane/isopropanol 100:0, λ = 240 nm, 0.5 mL/min, t (minor) = 12.18 min, t (minor) = 13.15 min].

$[\alpha]_D^{23}$ = +89.0 (*c 1.0*, $CHCl_3$); ^{1}**H NMR** ($CDCl_3$, 400 MHz) δ (ppm): 7.30 - 7.26 (m, 4 H), 7.24 - 7.19 (m. 1 H), 7.12 – 7.08 (m, 2 H), 6.80 – 6.75 (m, 2 H), 4.07 (dd, *J* = 8.5 Hz, 7.0 Hz, 1 H), 3.75 (s, 3 H), 3.48 (d, *J* = 13.5 Hz, 1 H), 3.39 (d, *J* = 13.5 Hz, 1 H), 2.74 (dd, *J* = 15.0, 7.5 Hz, 1 H), 2.69 (dd, *J* = 15.0, 8.5 Hz, 1 H), 1.25 (s, 9 H); ^{13}**C NMR** ($CDCl_3$, 100 MHz) δ (ppm): 169.9, 158.7, 141.3, 130.1, 129.9, 128.5, 128.1, 127.5, 114.0, 80.9, 55.4, 45.3, 42.7, 35.1, 28.0; **HRMS** (ESI+) mass calculated for $[M+Na]^+$ ($C_{21}H_{26}O_3NaS$) requires *m/z* 381.1495, found *m/z* 381.1495. **IR** (film) ν_{max}/cm^{-1}: 2977, 1729, 1511, 1367, 1249, 1174, 1140, 832, 750, 700.

***tert*-Butyl (*S*)-3-(propylthio)pentanoate 4o**

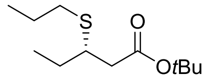

To a solution of *tert*-butyl (*E*)-pent-2-enoate **2g** (31 mg, 0.20 mmol, 1.00 eq) and **1g** (0.020 mmol, 0.10 eq) in Et_2O (0.40 mL) at 0 °C was added 1-propanethiol **3a** (56 µL, 0.60 mmol, 3.0 eq) according to a modified General Procedure **E** stirring for 72 h at 0 °C. The reaction mixture was purified by FCC (petroleum ether: Et_2O 99:1) to afford **4o** as a colourless oil in 84% yield (39 mg) and 93:7 er [determined by HPLC, chiralpak IA, hexane/isopropanol 100:0, λ = 220 nm, 1.0 mL/min, t (major) = 8.37 min, t (minor) = 9.86 min].

$[\alpha]_D^{23}$ = -1.2 (*c* 1.24, $CHCl_3$); ^{1}**H NMR** ($CDCl_3$, 400 MHz) δ (ppm): 2.99 - 2.92 (m, 1 H), 2.52 - 2.46 (m, 4 H), 1.68 - 1.55 (m, 4 H), 1.00 (t, *J* = 7.5 Hz, 3 H), 0.98 (t, *J* = 7.5 Hz, 3 H); ^{13}**C NMR** ($CDCl_3$, 100 MHz) δ (ppm): 171.3, 80.8, 43.7, 42.0, 32.9, 28.2, 28.1, 23.3, 13.7, 11.4; **HRMS** (ESI+) mass calculated for $[M+Na]^+$ ($C_{12}H_{24}O_2NaS$) requires *m/z* 255.1389, found *m/z* 255.1384. **IR** (film) ν_{max}/cm^{-1}: 2965, 2931, 1729, 1368, 1148, 909, 733.

tert-Butyl (S)-3-(propylthio)decanoate 4p

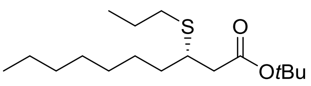

To a solution of *tert*-butyl (*E*)-dec-2-enoate **2h** (45.2mg, 0.20 mmol, 1.00 eq) and **1g** (0.020 mmol, 0.10 eq) in Et_2O (0.40 mL) at 0 °C was added 1-propanethiol **3a** (56 µL, 0.60 mmol, 3.0 eq) according to a modified General Procedure **E** stirring for 72 h at 0 °C. The product was purified by FCC (99:1 petrol: Et_2O) to afford **4p** as a colourless oil in 89% yield (54.0 mg) and 92:8 er [determined by HPLC, chiralpak IA, hexane/isopropanol 100:0, λ = 240 nm, 1.0 mL/min, t (major) = 15.44 min, t (minor) = 16.75 min].

$[\alpha]_D^{23}$ = -2.1 (*c* 1.0, $CHCl_3$); **^{1}H NMR** ($CDCl_3$, 400 MHz) δ (ppm): 3.01 - 2.95 (m, 1 H), 2.52 - 2.43 (m, 4 H), 1.64 – 1.52 (m, 5 H), 1.45 (s, 9 H), 1.32 – 1.21 (m, 9 H), 0.97 (t, *J* = 7.5 Hz, 3 H), 0.87 (t, *J* = 7.0 Hz, 3 H); **^{13}C NMR** ($CDCl_3$, 100 MHz) δ (ppm): 171.3, 80.7, 42.4, 42.0, 35.2, 32.8, 31.9, 29.6, 29.3, 28.2, 26.9, 23.3, 22.8, 14.2, 13.7; **HRMS** (ESI+) mass calculated for $[M+Na]^+$ ($C_{17}H_{34}O_2NaS$) requires *m/z* 325.2172, found *m/z* 325.2172; **IR** (film) ν_{max}/cm^{-1}: 2928, 2857, 1729, 1368, 1147, 757.

tert-Butyl (S)-3-(propylthio)hept-6-enoate 4q

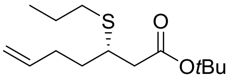

To a solution of *tert*-butyl (*E*)-hepta-2,6-dienoate **2i** (36.4 mg, 0.20 mmol, 1.0 eq) and **1g** (0.020 mmol, 0.10 eq) in Et_2O (0.40 mL) at 0 °C was added 1-propanethiol **3a** (56 µL, 0.60 mmol, 3.0 eq) according to a modified General Procedure **E** stirring for 48 h at 0 °C. The product was purified by FCC (99:1 petrol: Et_2O) to afford **4q** as a colourless oil in 84% yield (43.0 mg) and 94:6 er [chiralpak IA, hexane/isopropanol 100:0, λ = 230 nm, 1.0 mL/min, t (major) = 9.73 min, t (minor) = 11.36 min].

$[\alpha]_D^{23}$ = -6.4 (*c* 0.9, $CHCl_3$); **^{1}H NMR** ($CDCl_3$, 400 MHz) δ (ppm): 5.78 (ddt, *J* = 17.0, 10.0, 6.5 Hz, 1 H), 5.03 (‘dq’, *J* = 17.0, 1.5 Hz, 1 H), 4.96 (‘dq’, *J* = 10.0, 1.5 Hz, 1 H), 2.99 (‘qd’, *J* = 7.5, 5.5 Hz, 1 H), 2.54 – 2.41 (m, 4 H), 2.29 – 2.12 (m , 2 H), 1.71 – 1.52 (m, 4 H), 1.44 (s, 9 H), 0.97 (t, *J* = 7.5 Hz, 3 H); **^{13}C NMR** ($CDCl_3$, 100 MHz) δ (ppm): 171.1, 138.1, 115.2, 80.8, 42.5, 41.5, 34.3, 32.8, 31.1, 28.2, 23.2,13.7; **HRMS** (ESI+) mass calculated for $[M+Na]^+$ ($C_{14}H_{26}O_2NaS$) requires *m/z* 281.1546, found *m/z* 281.1533; **IR** (film) ν_{max}/cm^{-1}: 2967, 2931, 1727, 1367, 1250, 1144, 909, 731.

tert-Butyl (_S_)-5-phenyl-3-(propylthio)pentanoate 4r

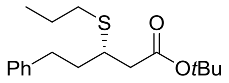

To a solution of _tert_-butyl (_E_)-5-phenylpent-2-enoate **2j** (46.4 mg, 0.20 mmol, 1.0 eq) and **1g** (0.020 mmol, 0.10 eq) in Et_2O (0.40 mL) at 0 °C was added 1-propanethiol **3a** (56 µL, 0.60 mmol, 3.0 eq) according to a modified General Procedure **E** stirring for 72 h at 0 °C. The product was purified by FCC (99:1 petrol: Et_2O) to afford **4r** as a colourless oil in 88% yield (54.0 mg) and 94:6 er [chiralpak IA, hexane/isopropanol 100:0, λ = 210 nm, 1.0 mL/min, t (major) = 17.39 min, t (minor) = 20.08 min].

$[\alpha]_D^{23}$ = -6.6 (_c_ 1.14, $CHCl_3$); **^{1}H NMR** ($CDCl_3$, 400 MHz) δ (ppm): 7.31 – 7.26 (m, 2 H), 7.22 – 7.17 (m, 3 H), 3.03 (‘qd’, _J_ = 7.5, 5.0 Hz, 1 H), 2.88 – 2.71 (m, 2 H), 2.59 – 2.45 (m, 4 H), 1.97 – 1.79 (m, 2 H), 1.66 – 1.56 (m, 2 H), 1.45 (s, 9 H), 1.00 (t, _J_ = 7.5 Hz, 3 H); **^{13}C NMR** ($CDCl_3$, 100 MHz) δ (ppm): 171.0, 141.9, 128.6, 128.5, 126.0, 80.8, 42.5, 41.6, 36.8, 33.1, 32.7, 28.2, 23.2, 13.7; **HRMS** (ESI+) mass calculated for $[M+Na]^+$ ($C_{18}H_{28}O_2NaS$) requires _m/z_ 331.1702, found _m/z_ 331.1702. **IR** (film) ν_{max}/cm^{-1}: 2964, 1726, 1454, 1367, 1249, 1141, 847, 748, 693.

tert-Butyl (_S_)-5-((_tert_-butoxycarbonyl)amino)-3-(propylthio)pentanoate 4s

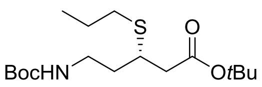

To a solution of _tert_-butyl (_E_)-5-((tert-butoxycarbonyl)amino)pent-2-enoate **2k** (54.2 mg, 0.20 mmol, 1.00 eq) at 0 °C was added 1-propanethiol **3a** (56 µL, 0.60 mmol, 3.0 eq) according to a modified General Procedure **E** stirring for 72 h at 0 °C. The product was purified by FCC (99:1 petrol: Et_2O) to afford **4s** as a colourless oil in 81% yield (56.0 mg) and 94:6 er [determined by HPLC, chiralpak AD-H, hexane/isopropanol 99:1, λ = 210 nm, 1.0 mL/min, t (minor) = 17.56 min, t (major) = 18.79 min].

$[\alpha]_D^{23}$ = - 7.3 (_c_ 0.8, $CHCl_3$); **^{1}H NMR** ($CDCl_3$, 400 MHz) δ (ppm): 4.77 (br s, 1 H), 3.33 – 3.18 (m, 2 H), 3.04 – 2.96 (m, 1H), 2.55 - 2.42 (m, 4 H), 1.85 - 1.62 (m, 2 H), 1.61 - 1.53 (m, 2 H), 1.44 (s, 9 H), 1.42 (s, 9 H), 0.96 (t, _J_ = 7.5 Hz, 3 H); **^{13}C NMR** ($CDCl_3$, 100 MHz) δ (ppm): 171.0, 156.0, 81.0, 79.3, 42.4, 39.5, 38.5, 34.9, 32.6, 28.5, 28.2, 23.2, 13.7; **HRMS** (ESI+) mass calculated for $[M+Na]^+$ ($C_{17}H_{33}NNaO_4NaS$) requires _m/z_ 370.2023, found _m/z_ 370.2019; **IR** (film) ν_{max}/cm^{-1}: 3470, 2979, 2932, 1713, 1504, 1366, 1249, 1144, 736.

tert-Butyl (S)-5-((tert-butyldimethylsilyl)oxy)-3-(propylthio)pentanoate 4t

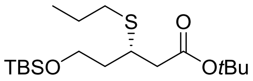

To a solution of *tert*-butyl (*E*)-5-((*tert*-butyldimethylsilyl)oxy)pent-2-enoate **2l** (57.2 mg, 0.20 mmol, 1.0 eq) at 0 °C was added 1-propanethiol **3a** (56 μL, 0.60 mmol, 3.0 eq) according to a modified General Procedure **E** stirring for 72 h at 0 °C. The product was purified by FCC (99:1 petrol: Et_2O) to afford **4t** as a colourless oil in 97% yield (70.0 mg) and 94:6 er [determined by HPLC, chiralpak IA, hexane/isopropanol 100:0, λ = 230 nm, 1.0 mL/min, t (major) = 15.25 min, t (minor) = 17.40 min].

$[\alpha]_D^{23}$ = -6.8 (*c* 1.2, $CHCl_3$); **^{1}H NMR** ($CDCl_3$, 400 MHz) δ (ppm): 3.81 - 3.68 (m, 2 H), 3.20 - 3.08 (m, 1 H), 2.54 – 2.45 (m, 4 H), 1.84 – 1.66 (m, 2 H), 1.59 (sxt, *J* = 7.5 Hz, 2 H), 1.45 (s, 9 H), 0.97 (t, *J* = 7.5 Hz, 3 H), 0.88 (s, 9 H), 0.04 (s, 6 H); **^{13}C NMR** ($CDCl_3$, 100 MHz) δ (ppm): 171.0, 80.7, 60.5, 42.7, 38.8, 38.2, 32.9, 28.2, 26.1, 23.3, 18.4, 13.7, -5.2, -5.2; **HRMS** (ESI+) mass calculated for $[M+H]^+$ ($C_{18}H_{39}O_3SSi$) requires *m/z* 363.2384, found *m/z* 363.2376. **IR** (film) ν_{max}/cm^{-1}: 2958, 2930, 2858, 1731, 1472, 1367, 1255, 1147, 1100, 836, 776, 735.

1.6 Preparative scale SMA reaction with 1 mol% catalyst

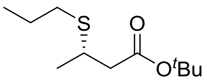

To a solution of *tert*-butyl crotonate **2e** (1.12 mL, 7.0 mmol, 1.0 eq) and **1g** (0.070 mmol, 0.010 eq) in Et_2O (1.4 mL) at 0 °C was added 1-propanethiol **3a** (1.89 mL, 21.0 mmol, 3.0 eq) and stirring was maintained for 24 h whereupon the reaction mixture was quenched by the addition of 1.0 M AcOH (in CH_2Cl_2, 0.7 mL). The volatiles were removed under a stream of nitrogen and the crude reaction mixture was purified by FCC (99:1 petrol: Et_2O) to afford **4e** as a colourless oil in 97% yield (1.48 g) and 94:6 er [determined by HPLC, chiralpak IA, hexane/isopropanol 100:0, λ 210 nm, 1.0 mL/min, t (major) = 9.36 min, t (minor) = 10.58 min]. Spectroscopic data consistent with that reported in section 1.5.

1.7 Derivatisation of β-Mercaptoester products

tert-Butyl (_S_)-3-(propylsulfonyl)butanoate 5a

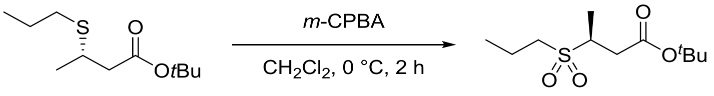

To a solution of 3-chloroperbenzoic acid (86 mg, 0.5 mmol, 2.5 eq) in dichloromethane (5 mL) was added *tert*-butyl (*S*)-3-(propylthio)butanoate **4e** (43.6 mg, 0.2 mmol, 1.0 eq, 94:6 er) in dichloromethane (1 mL) at 0 ˚C. The reaction was stirred at 0 ˚C for 2 h, and then quenched with $NaHCO_3$ (2 mL). The mixture was extracted with Et_2O (3 x 5 mL) and the organic layers combined, dried (Na_2SO_4), filtered and concentrated *in vacuo.* The crude reaction mixture was purified by FCC to afford the title compound **5a** as a colourless oil in 96% yield (48 mg) and 94:6 er [determined by HPLC, chiralpak IA, hexane/isopropanol 95:5, λ = 220 nm, 1.0 mL/min, t (major) = 10.93 min, t (major) = 14.85 min].

$[\alpha]_D^{23}$ = +4.6 (*c 1.1*, $CHCl_3$); **^{1}H NMR** ($CDCl_3$, 400 MHz) δ (ppm): 3.50 - 3.41 (m, 1 H), 2.97 (dd, *J* = 16.5, 4.5 Hz, 1 H), 2.92 (t, *J* = 8 Hz, 2 H), 2.39 (dd, *J* = 16.5, 9.5 Hz, 1 H), 1.96 - 1.88 (m, 2 H), 1.46 (s, 9 H), 1.41 (d, *J* = 7.0 Hz, 3 H), 1.08 (t, *J* = 7.5 Hz, 3 H); **^{13}C NMR** ($CDCl_3$, 100 MHz) δ (ppm): 169.6, 82.0, 54.1, 52.0, 35.3, 28.2, 15.2, 14.0, 13.4; **HRMS** (ESI+) mass calculated for $[M+Na]^+$ ($C_{11}H_{22}O_4NaS$) requires *m/z* 273.1131, found *m/z* 273.1132; **IR** (film) ν_{max}/cm^{-1}: 2980, 2360, 1729, 1155, 754.

Methyl (_S_)-3-(propylthio)butanoate 4a

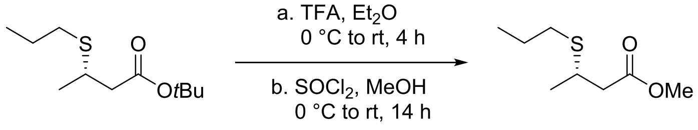

To a solution of *tert*-butyl (*S*)-3-(propylthio)butanoate **4e** (43.6 mg, 0.20 mmol, 1.00 eq, 96:4 er) in 1.0 mL Et_2O was added trifluoroacetic acid (0.1 mL) at 0 °C dropwise. The reaction mixture was warmed to rt and stirred for 4 h, and the volatiles removed by N_2 stream. Saturated aq $NaHCO_3$ (5 mL) was added into the mixture and the organic phase was extracted using Et_2O (2 x 5 mL), washed with brine (5 mL), dried ($NaSO_4$), filtered, and concentrated *in vacuo* to afford the crude mixture, which was used in the next step without purification. The carboxylic acid intermediate was dissolved in MeOH (5 mL). To the

solution was added $SOCl_2$ (0.12 mL, 0.44 mmol, 2.2 eq) at 0 ˚C. The reaction was warmed to room temperature and stirred overnight. The reaction mixture was then quenched with saturated aq $NaHCO_3$ and then extracted with CH_2Cl_2 (3 x 5 mL). The organic phase was combined, dried (Na_2SO_4), filtered and concentrated *in vacuo*. The crude reaction mixture was purified by FCC (99:1 petrol: Et_2O) to afford the title compound **5b** as a colourless oil in 78% yield (27 mg) and 94:6 er [determined by HPLC, chiralpak IA, hexane/isopropanol 100:0, λ = 220 nm, 1.0 mL/min, t (major) = 19.96 min, t (minor) = 23.58 min]; **$[\alpha]_D^{25}$** = + 9.8 (c 0.9, $CHCl_3$). Other data consistent with that reported in section 1.5.

(*S*)-3-((4-Methoxybenzyl)thio)butan-1-ol 5b

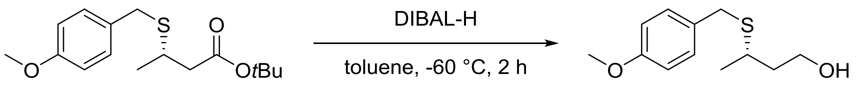

To a solution of *tert*-butyl (*S*)-3-((4-methoxybenzyl)thio)butanoate **4m** (28 mg, 0.095 mmol, 1.0 eq, 95:5 er) in toluene (1 mL) at -60˚C was added DIBAL-H (1 M in toluene, 0.2 mL, 0.20 mmol, 2.2 eq) dropwise. The reaction was stirred at -60˚C for 2 hours and then quenched with saturated NH_4Cl (aq), extracted three times with CH_2Cl_2 (3 x 5 mL). The combined organics were dried (Na_2SO_4), filtered and concentrated *in vacuo*. The crude mixture was purified by FCC to afford the title compound **5c** as a colourless oil 93% yield (20 mg) and 93:7 er [determined by HPLC, chiralpak AD-H, hexane/isopropanol 97:3, λ = 220 nm, 1.0 mL/min, t (minor) = 37.72 min, t (major) = 39.48 min].

$[\alpha]_D^{23}$ = - 0.2 (*c 1.0*, $CHCl_3$); **^{1}H NMR** ($CDCl_3$, 400 MHz) δ (ppm): 7.28 (d, *J* = 8.5 Hz, 2 H), 6.87 (d, *J* = 8.5 Hz, 2 H), 3.82 (s, 3 H), 3.78 – 3.66 (m, 4 H), 2.84 (sxt, *J* = 6.5 Hz, 1 H), 2.16 (s, 1 H), 1.79 (q, *J* = 6.5 Hz, 2 H), 1.34 (d, *J* = 6.5 Hz, 3 H); **^{13}C NMR** ($CDCl_3$, 100 MHz) δ (ppm): 158.6, 130.4, 129.9, 113.9, 60.6, 55.3, 39.0, 36.7, 34.3, 21.7; **HRMS** (ESI+) mass calculated for $[M+Na]^+$ ($C_{12}H_{18}O_2NaS$) requires *m/z* 249.0920, found *m/z* 249.0921. **IR** (film) ν_{max}/cm^{-1}: 3390, 2957, 1510, 1236, 1032, 831, 734.

1.8 Determination of Absolute Stereochemistry

1,1,1,3,3,3-Hexafluoropropan-2-yl (*R*)-3-((4-methoxybenzyl)thio)-3-phenylpropanoate 12

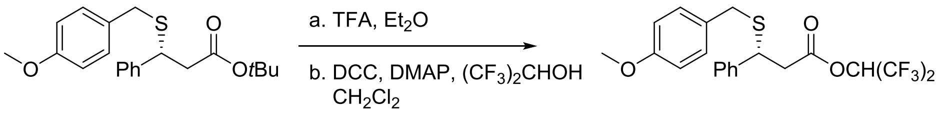

To a solution of *tert*-butyl (*R*)-3-((4-methoxybenzyl)thio)-3-phenylpropanoate **4n** (68.0 mg, 0.19 mmol, 1.00 eq, 88:12 er) in 1.0 mL Et_2O was added trifluoroacetic acid (0.1 mL) at 0 °C dropwise. The reaction mixture was warmed to rt and stirred for 4 h, and the volatiles removed under a N_2 stream to afford the crude mixture, which was used in the next step without purification. The carboxylic acid intermediate was dissolved in dichloromethane (5 mL). To the solution was added 4-dimethylaminopyridine (5 mg, 0.04 mmol) and *N,N'*-dicyclohexylcarbodiimide (49 mg, 0.24 mmol) and 1,1,1,3,3,3-hexafluoro-2-propanol (0.025 mL, 0.24 mmol) at 0 ˚C. The reaction was warmed to room temperature and stirred overnight. The reaction mixture was filtered and extracted three times with dichloromethane (5 mL). The organic phase was combined, dried (Na_2SO_4), filtered and concentrated *in vacuo*. The crude mixture was purified by FCC (99:1 petrol: Et_2O) to afford **12** as a colourless oil in 82% yield (70.0 mg) and 90:10 er [determined by HPLC, chiralpak AS-H, hexane/isopropanol 99:1, λ = 230 nm, 1.0 mL/min, t (major) = 6.68 min, t (minor) = 11.64 min; the order of elution of the two enantiomers is consistent to that given in the literature].[13]

$[\alpha]_D^{23}$ = +102.2 (*c* 0.85, $CHCl_3$), [lit.[13] $[\alpha]_D^{25}$ -65.9 (c 0.41, $CHCl_3$) for (*S*)-**12**]. From the optical rotation and the HPLC chromatograms, the absolute configuration of **12** was determined to be (*R*).[13]

^{1}H NMR ($CDCl_3$, 400 MHz) δ (ppm): 7.30 - 7.16 (m, 5 H), 7.05 (d, J = 8.5 Hz, 2 H), 6.75 (d, J = 8.5 Hz, 2 H), 5.57 (spt, J_{HF} = 6.1 Hz, 1 H), 4.06 (t, J = 8.0 Hz, 1 H), 3.72 (s, 3 H), 3.47 (d, J = 13.5 Hz, 1 H), 3.38 (d, J = 13.5 Hz, 1 H), 2.96 (d, J = 7.5 Hz, 2 H); **^{13}C NMR** ($CDCl_3$, 100 MHz) δ (ppm): 167.5, 158.9, 140.0, 130.1, 129.3, 128.9, 128.0, 127.8, 121.6 (q, J_{FC} = 280.0 Hz), 114.1, 66.6 (spt, J_{FC} = 34.6 Hz), 55.4, 44.4, 40.5, 35.3; **^{19}F NMR** ($CDCl_3$, 377 MHz) δ (ppm): -73.3. Data consistent with that reported in literature.[13]

2 References

(1) Núñez, M. G.; Farley, A. J. M.; Dixon, D. J. *J. Am. Chem. Soc.* **2013**, *135*, 16348-16351.

(2) Farley, A. J. M.; Sandford, C.; Dixon, D. J. *J. Am. Chem. Soc.* **2015**, *137*, 15992-15995.

(3) Raheem, I. T.; Jacobsen, E. N. *Adv. Synth. Cat.* **2005**, *347*, 1701-1708.

(4) Wu, Y.; Zhao, J.; Chen, J.; Pan, C.; Li, L.; Zhang, H. *Org. Lett.* **2009**, *11*, 597-600.

(5) Denmark, S. E.; Beutner, G. L. *J. Am. Chem. Soc.* **2003**, *125*, 7800-7801.

(6) Davies, S. G.; Lee, J. A.; Roberts, P. M.; Stonehouse, J. P.; Thomson, J. E. *J. Org. Chem.* **2012**, *77*, 7028-7045.

(7) Davies, S. G.; Fletcher, A. M.; Roberts, P. M.; Smith, A. D. *Tetrahedron* **2009**, *65*, 10192 - 10213.

(8) Claridge, T. D. W.; Davies, S. G.; Lee, J. A.; Nicholson, R. L.; Roberts, P. M.; Russell, A. J.; Smith, A. D.; Toms, S. M. *Org. Lett.* **2008**, *10*, 5437-5440.

(9) Kitajima, M.; Murakami, Y.; Takahashi, N.; Wu, Y.; Kogure, N.; Zhang, R.-P.; Takayama, H. *Org. Lett.* **2014**, *16*, 5000-5003.

(10) van der Westhuyzen, R.; Strauss, E. *J. Am. Chem. Soc.* **2010**, *132*, 12853-12855.

(11) Li, X.; Lantrip, D.; Fuchs, P. L. *J. Am. Chem. Soc.* **2003**, *125*, 14262 - 14263.

(12) Hawkins, J. M.; Lewis, T. A. *J. Org. Chem.* **1992**, *57*, 2114-2121.

(13) Fang, X.; Li, J.; Wang, C.-J. *Org. Lett.* **2013**, *15*, 3448-3451.

3 Supplementary Data

3.1 Copies of NMR spectra

^{1}H NMR of **9** (400 MHz, MeOD-d_4)

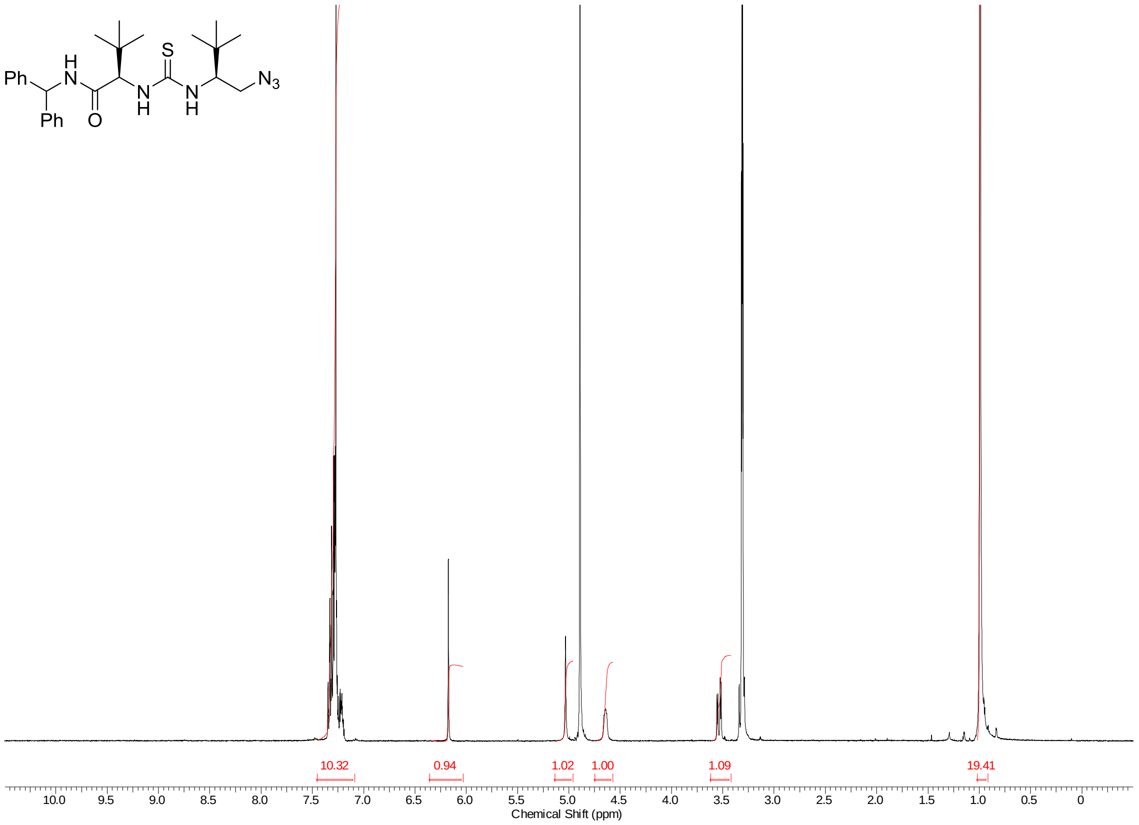

^{13}C NMR of **9** (125 MHz, MeOD-d_4)

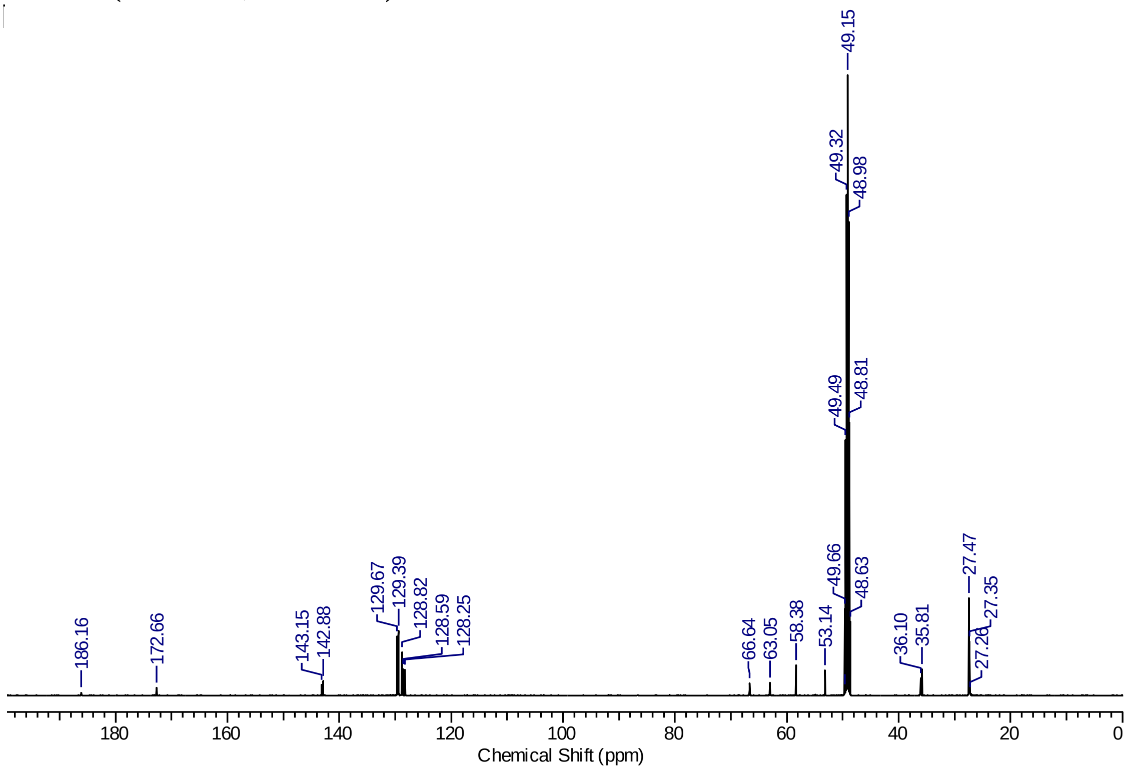

^{1}H NMR of **10** (400 MHz, MeOD-d_4)

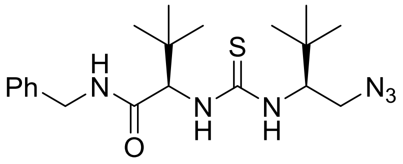

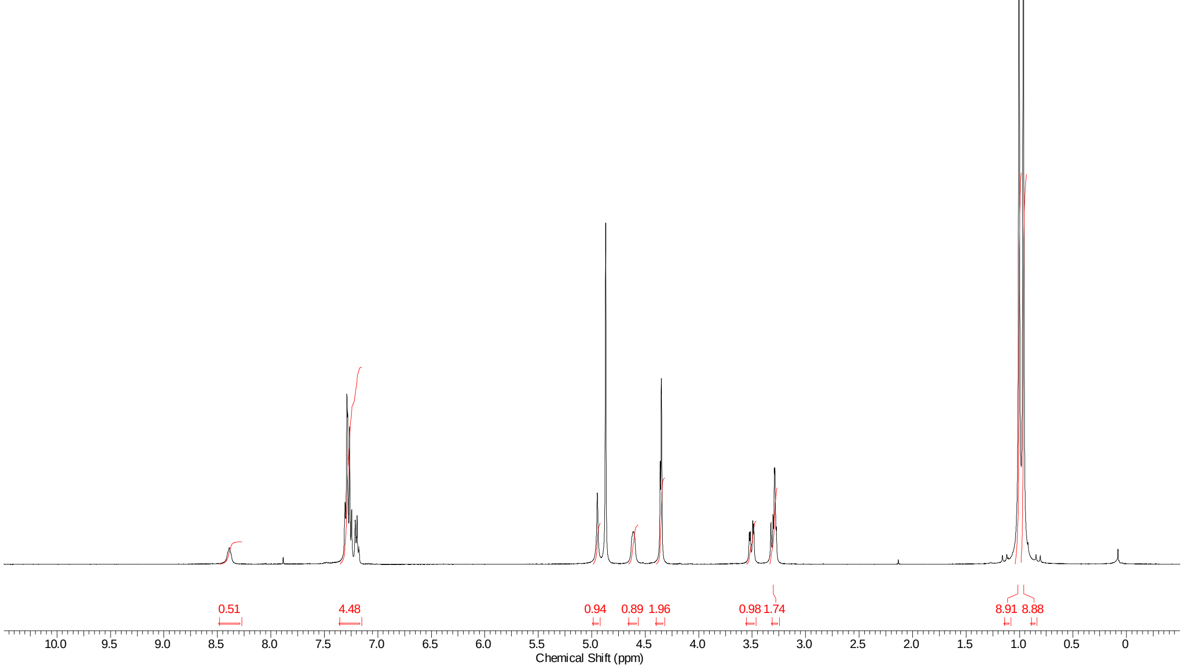

^{13}C NMR of **10** (100 MHz, MeOD-d_4)

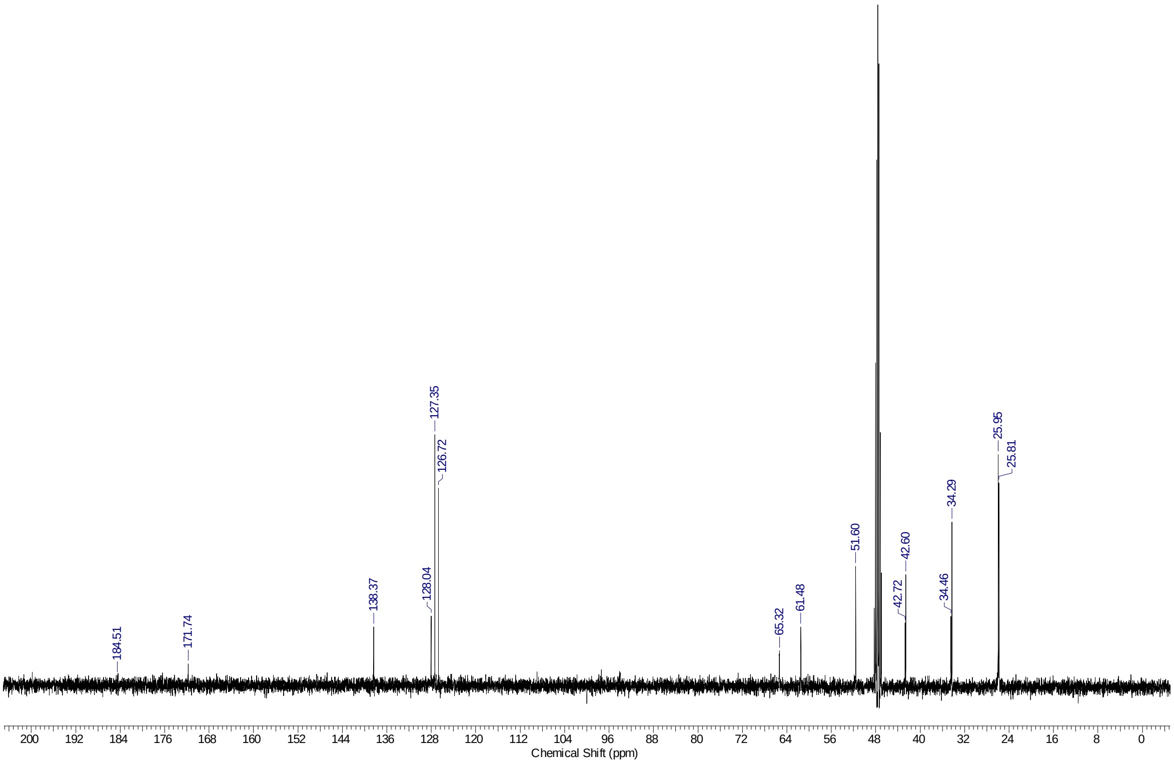

^{1}H NMR of **11** (400 MHz, MeOD-d$_4$)

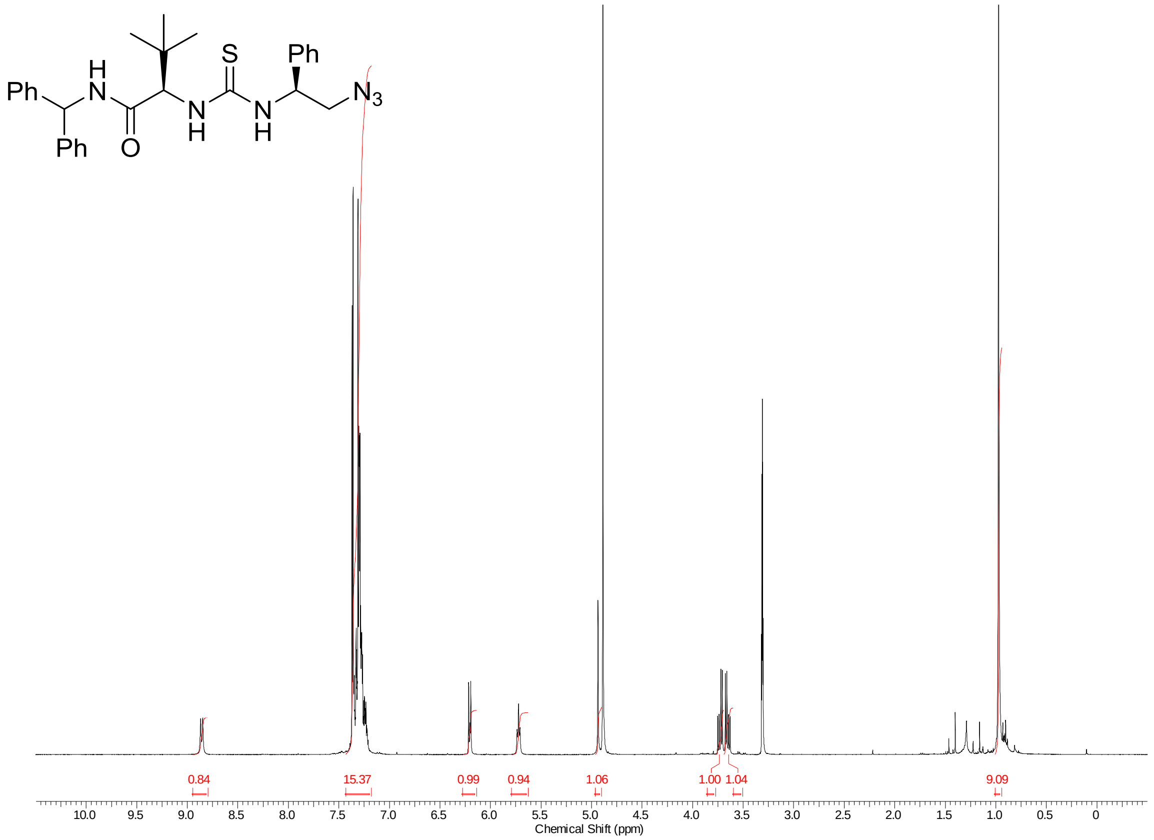

^{13}C NMR of **11** (100 MHz, MeOD-d$_4$)

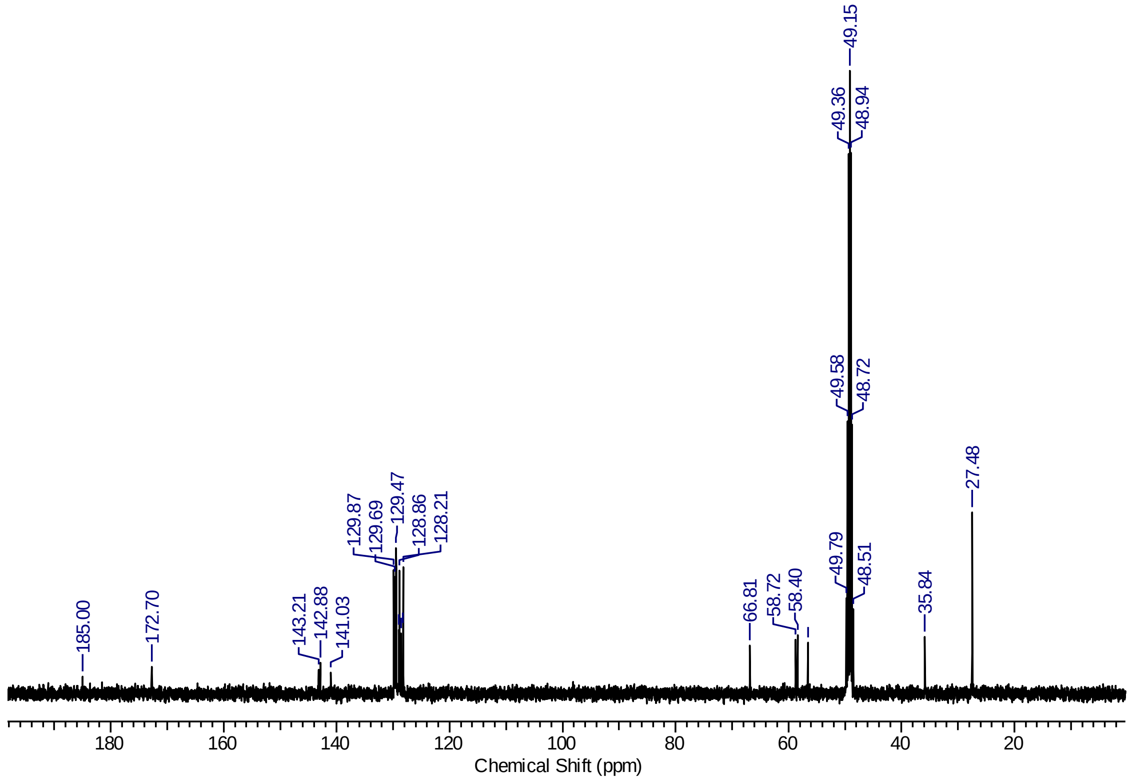

^{1}H NMR of **4a** (400 MHz, $CDCl_3$)

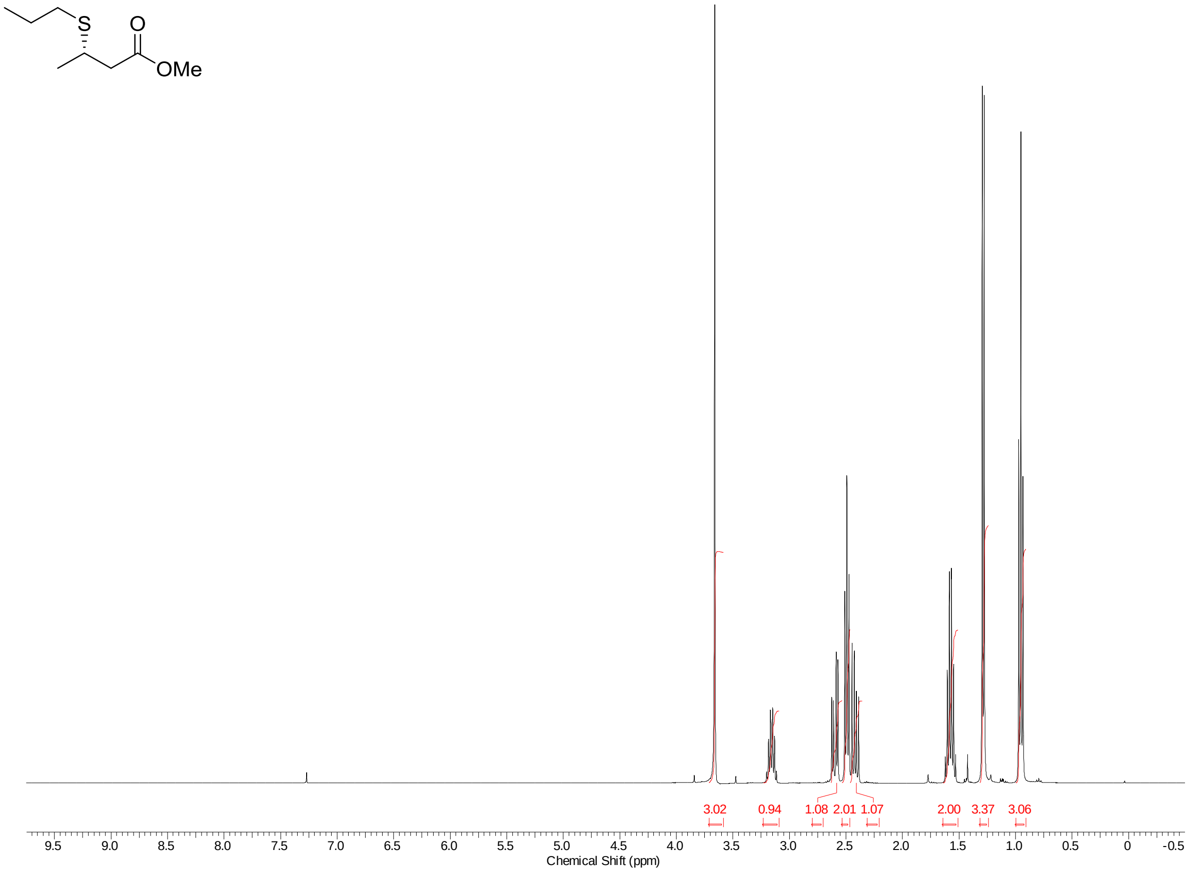

^{13}C NMR of **4a** (100 MHz, $CDCl_3$)

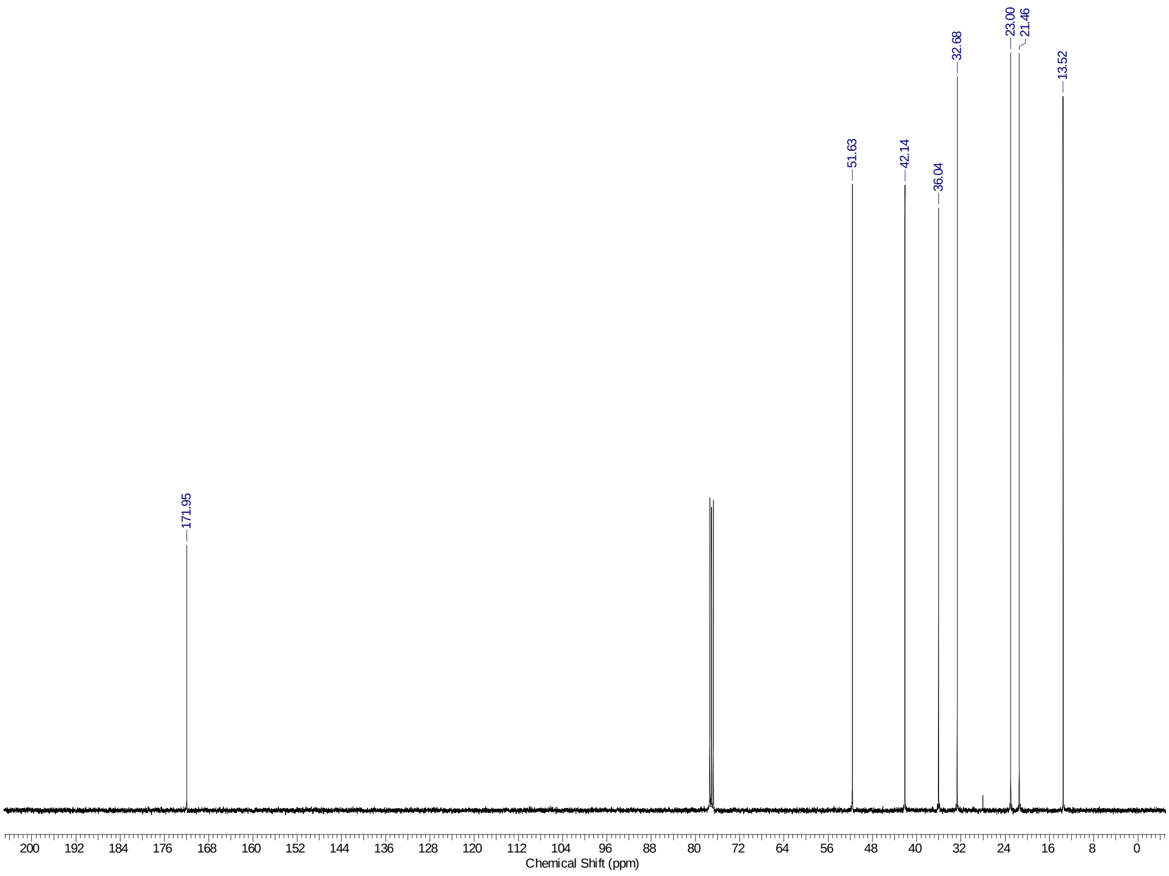

^{1}H NMR of **4b** (400 MHz, $CDCl_3$)

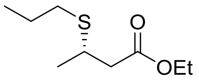

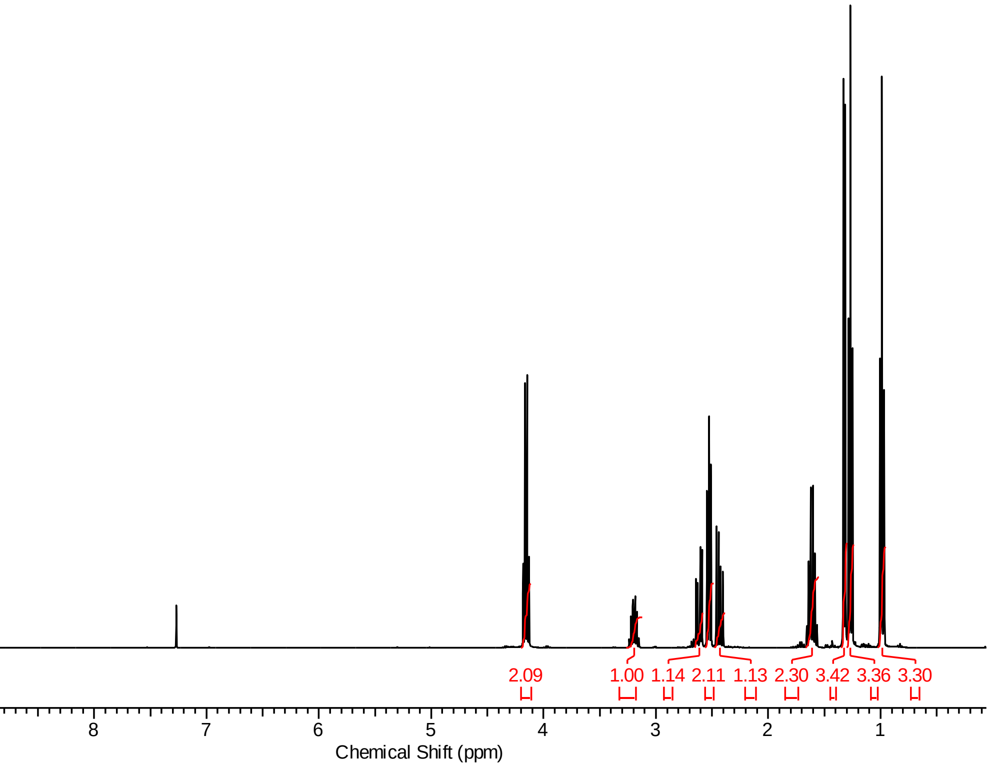

^{13}C NMR of **4b** (100 MHz, $CDCl_3$)

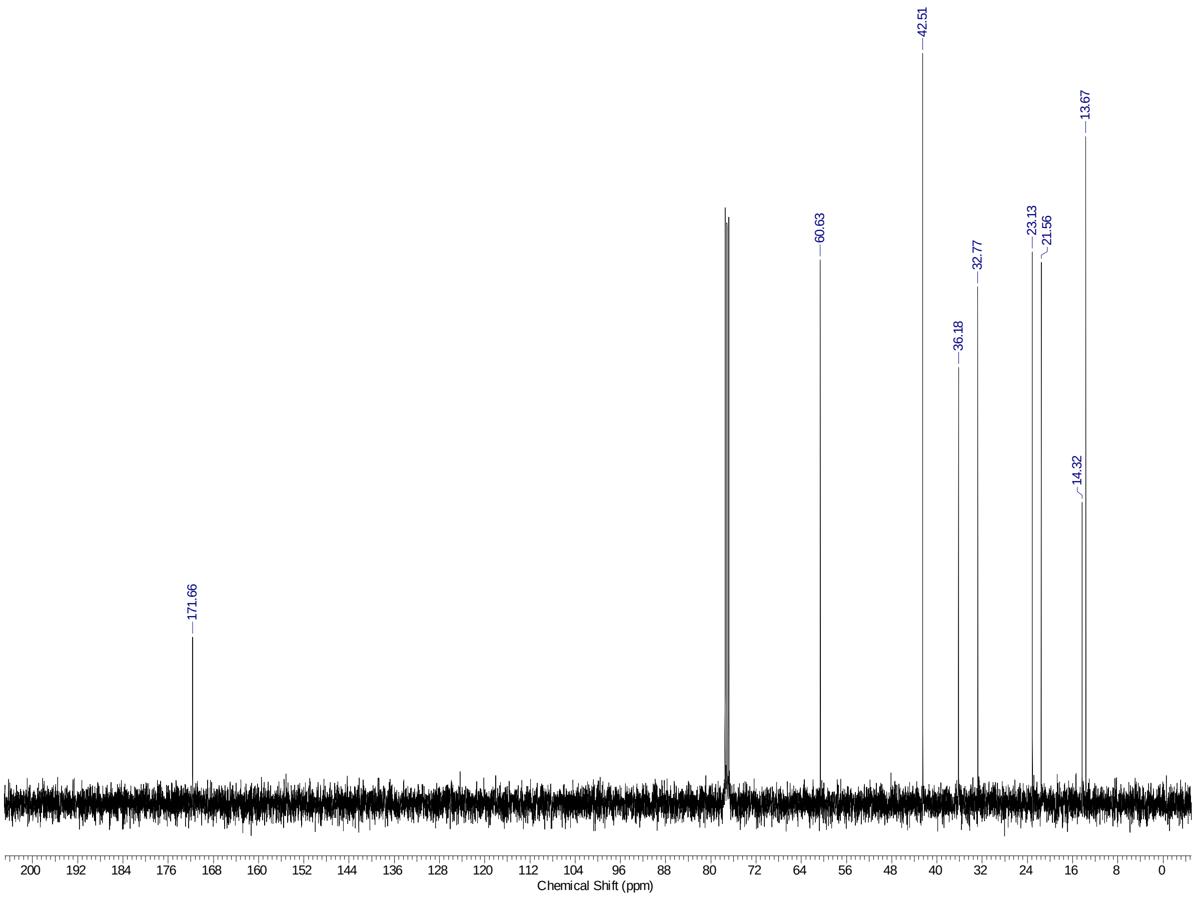

^{1}H NMR of **4c** (400 MHz, $CDCl_3$)

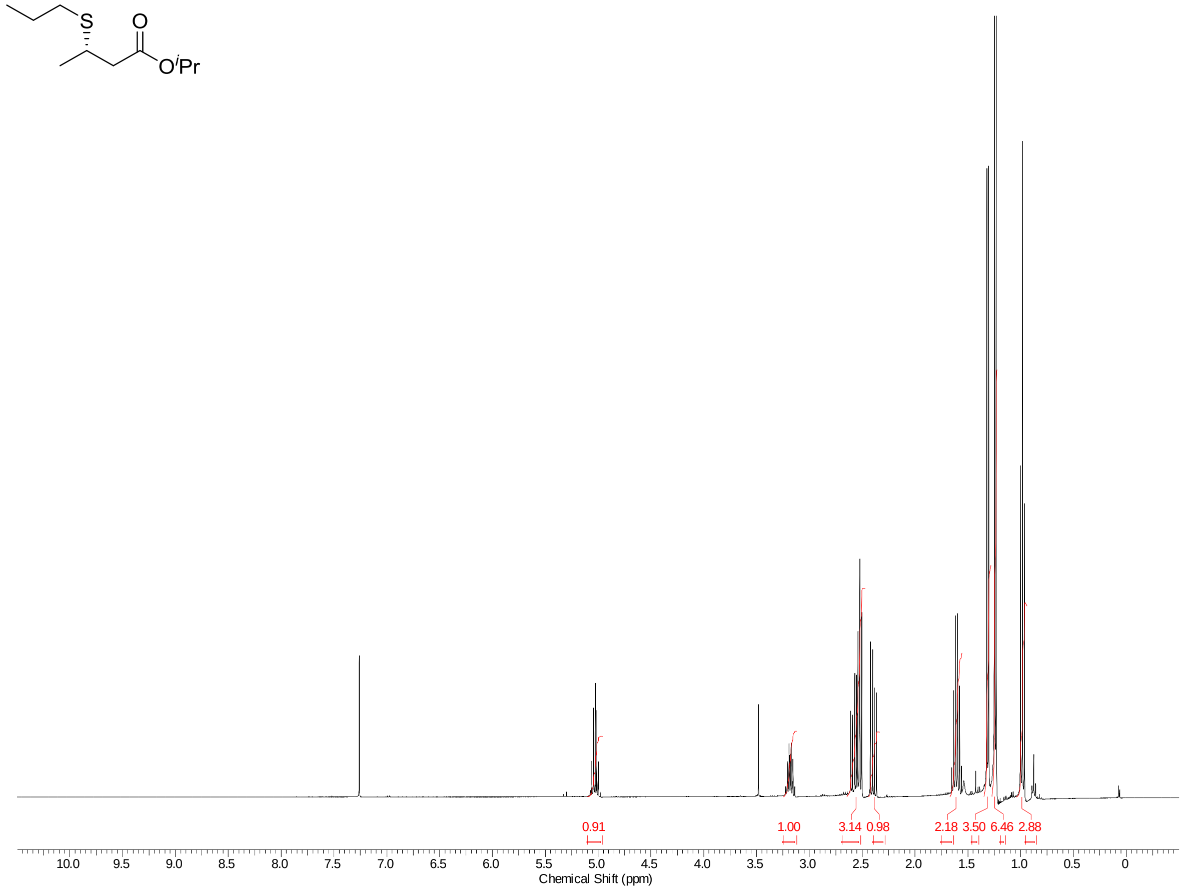

^{13}C NMR of **4c** (100 MHz, $CDCl_3$)

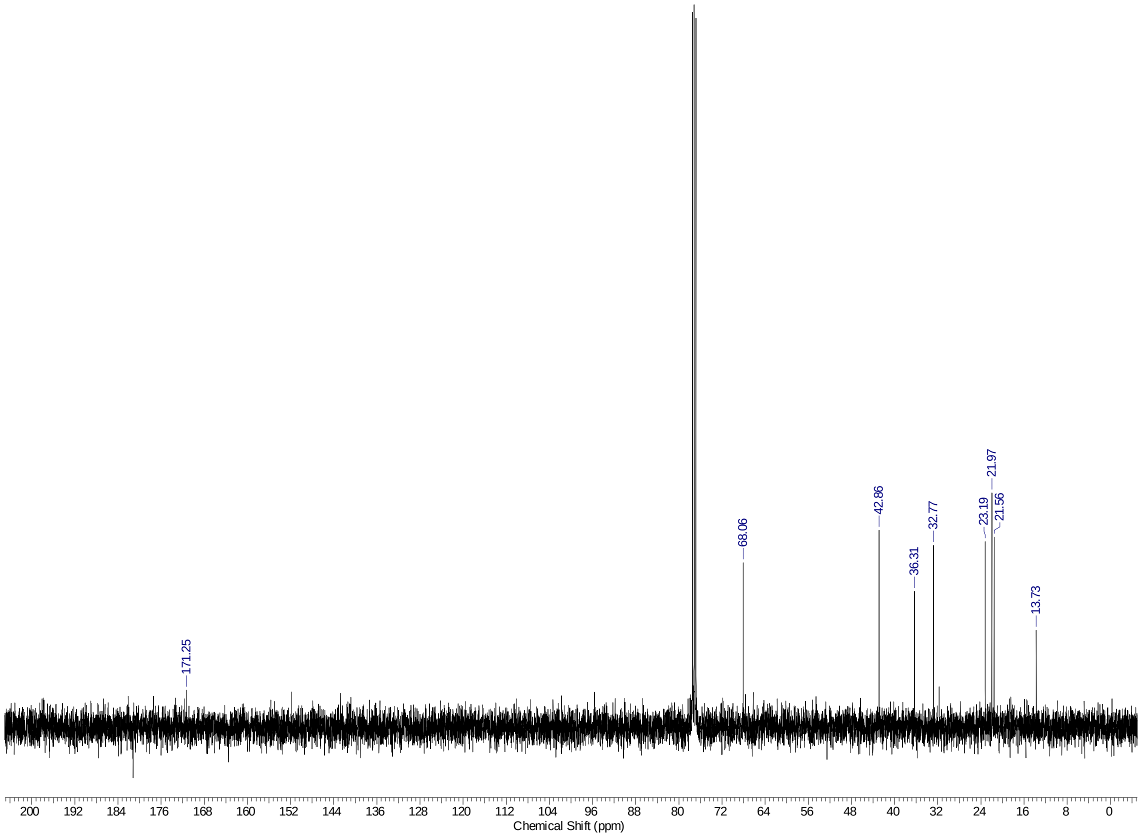

^{1}H NMR of **4d** (400 MHz, $CDCl_3$)

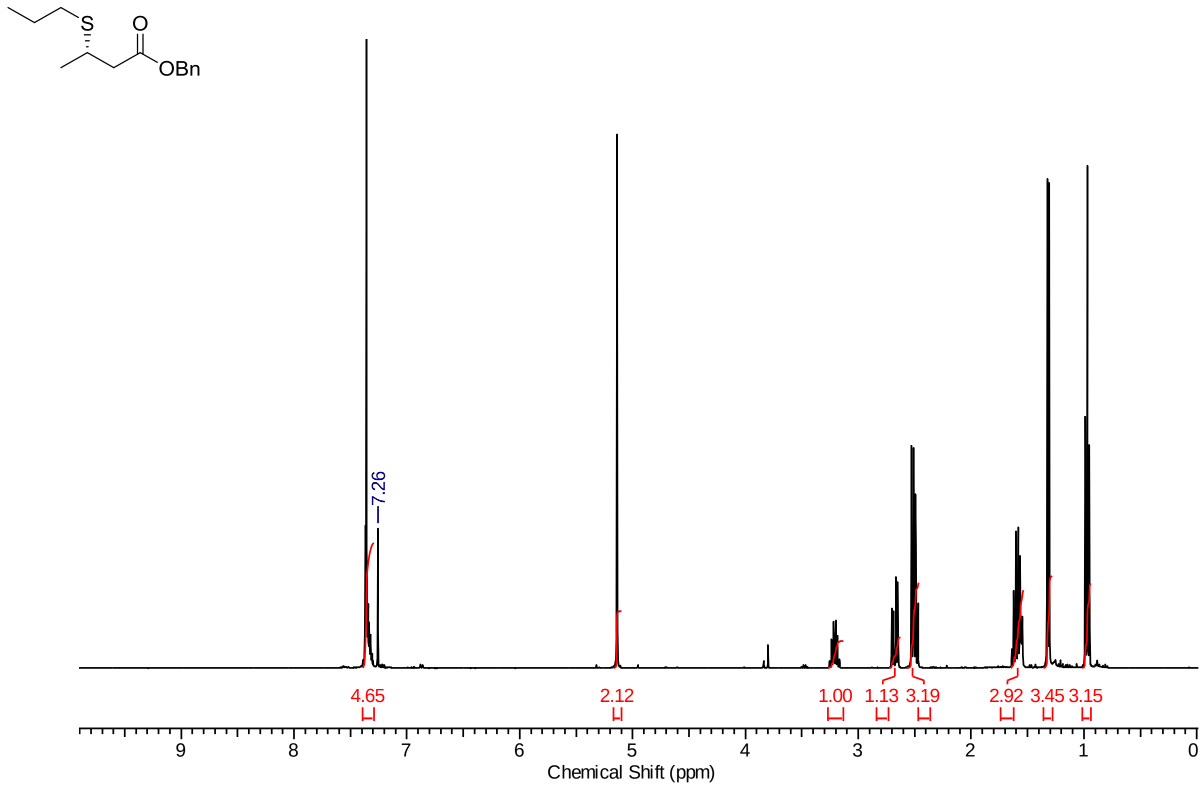

^{13}C NMR of **4d** (100 MHz, $CDCl_3$)

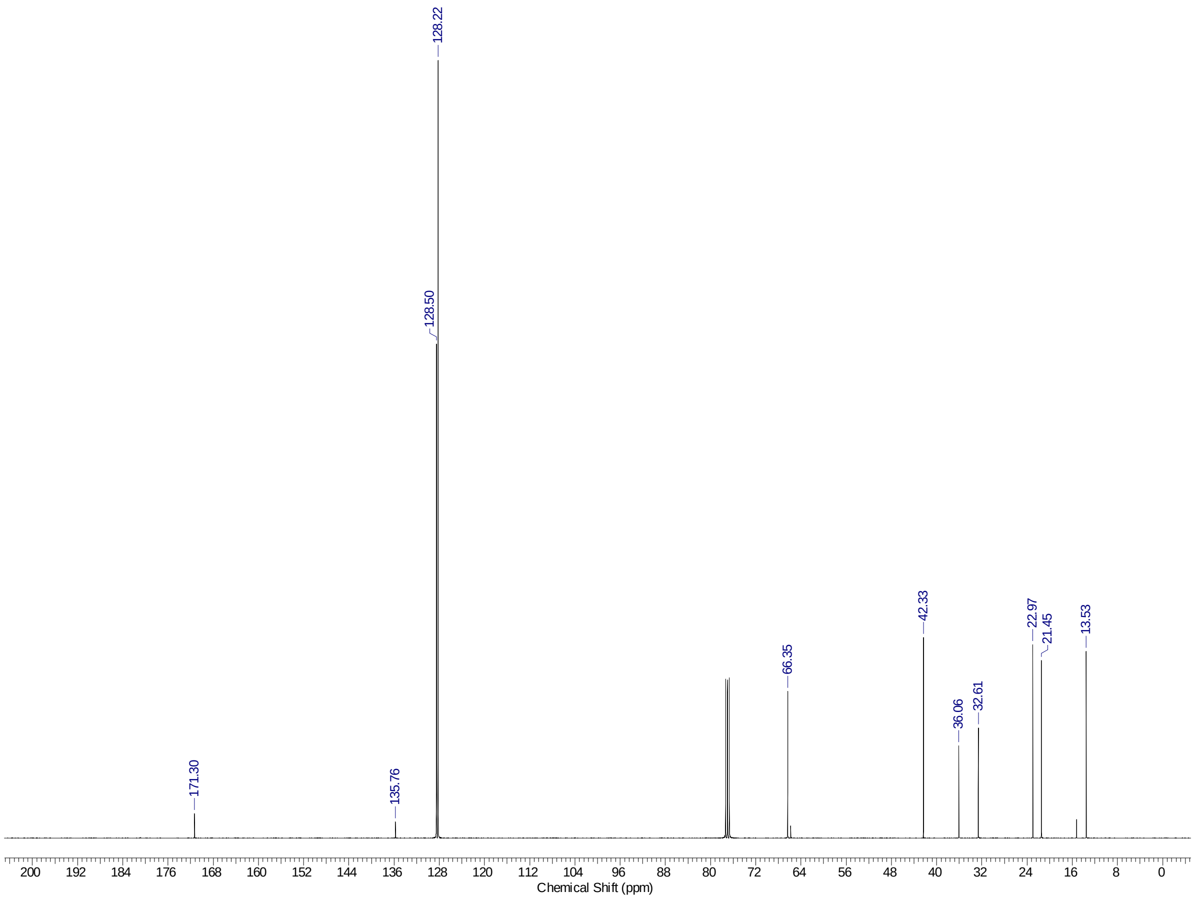

^{1}H NMR of **4e** (400 MHz, $CDCl_3$)

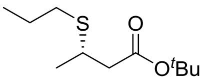

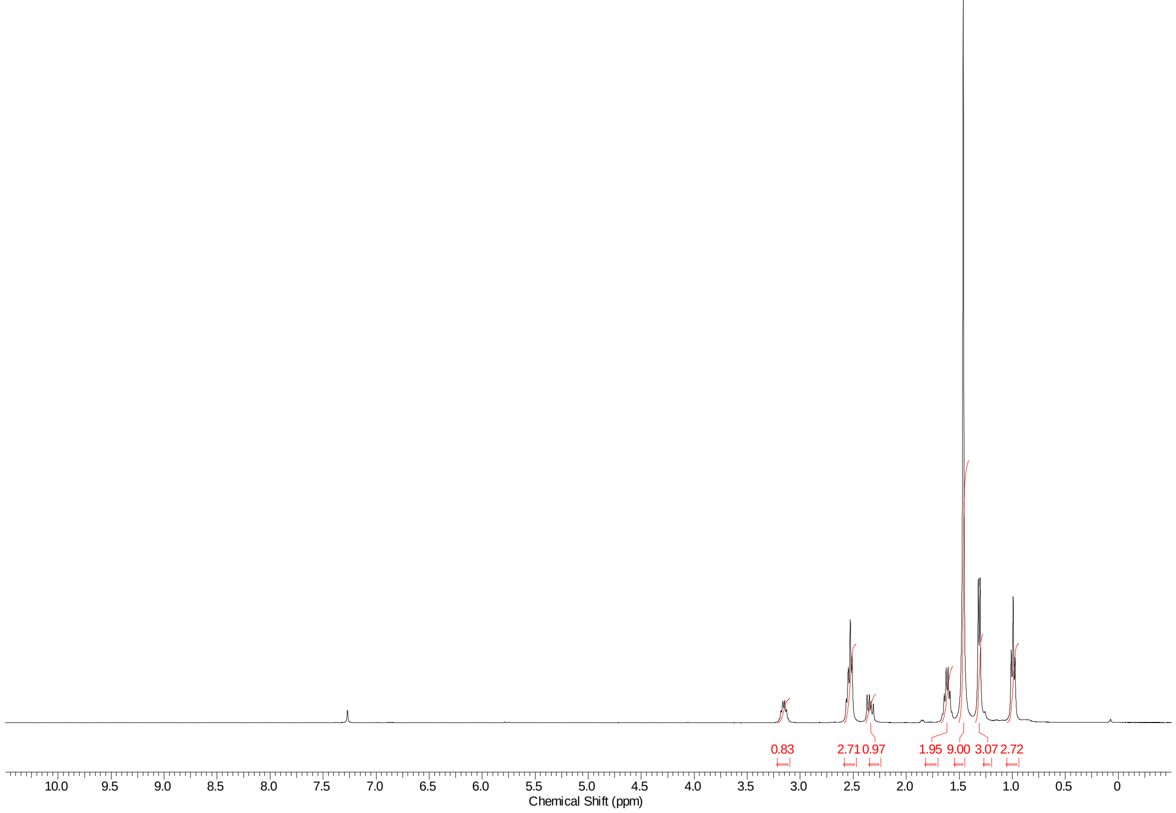

^{13}C NMR of **4e** (100 MHz, $CDCl_3$)

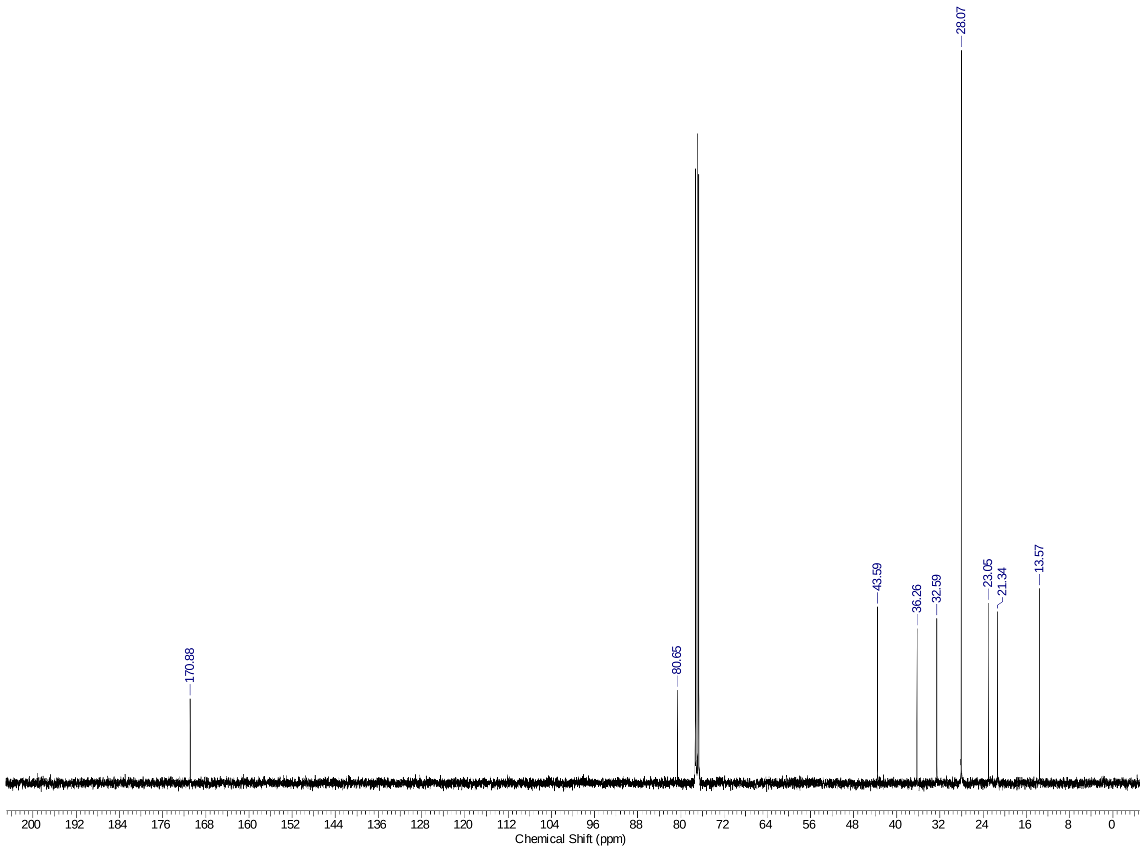

^{1}H NMR of **4f** (400 MHz, $CDCl_3$)

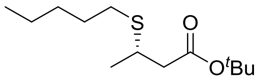

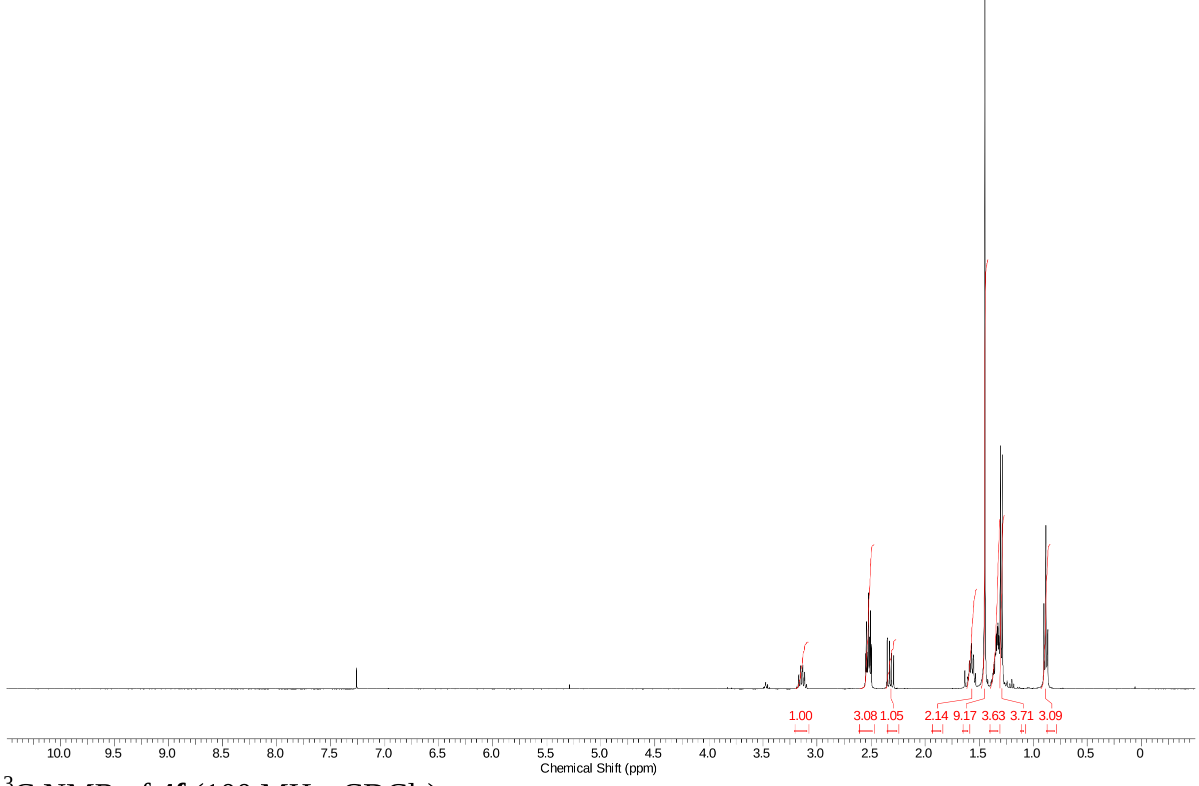

^{13}C NMR of **4f** (100 MHz, $CDCl_3$)

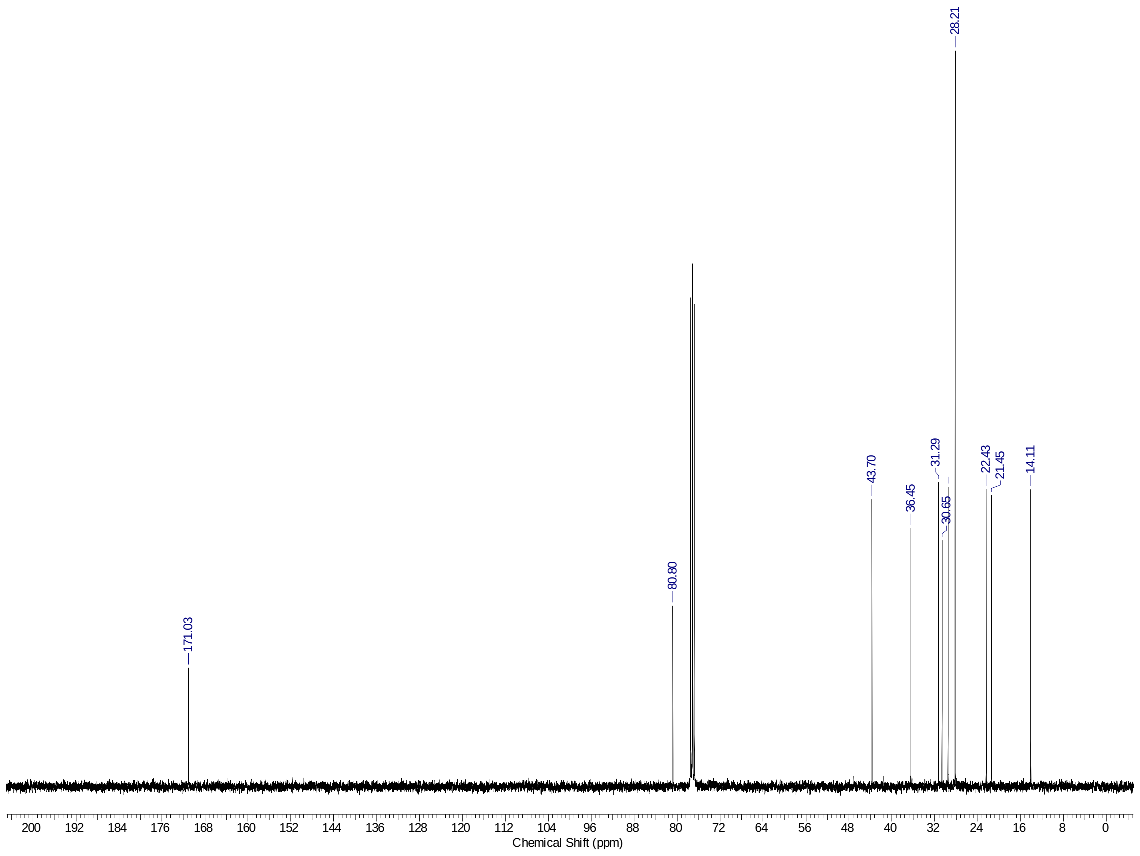

^{1}H NMR of **4g** (400 MHz, $CDCl_3$)

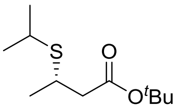

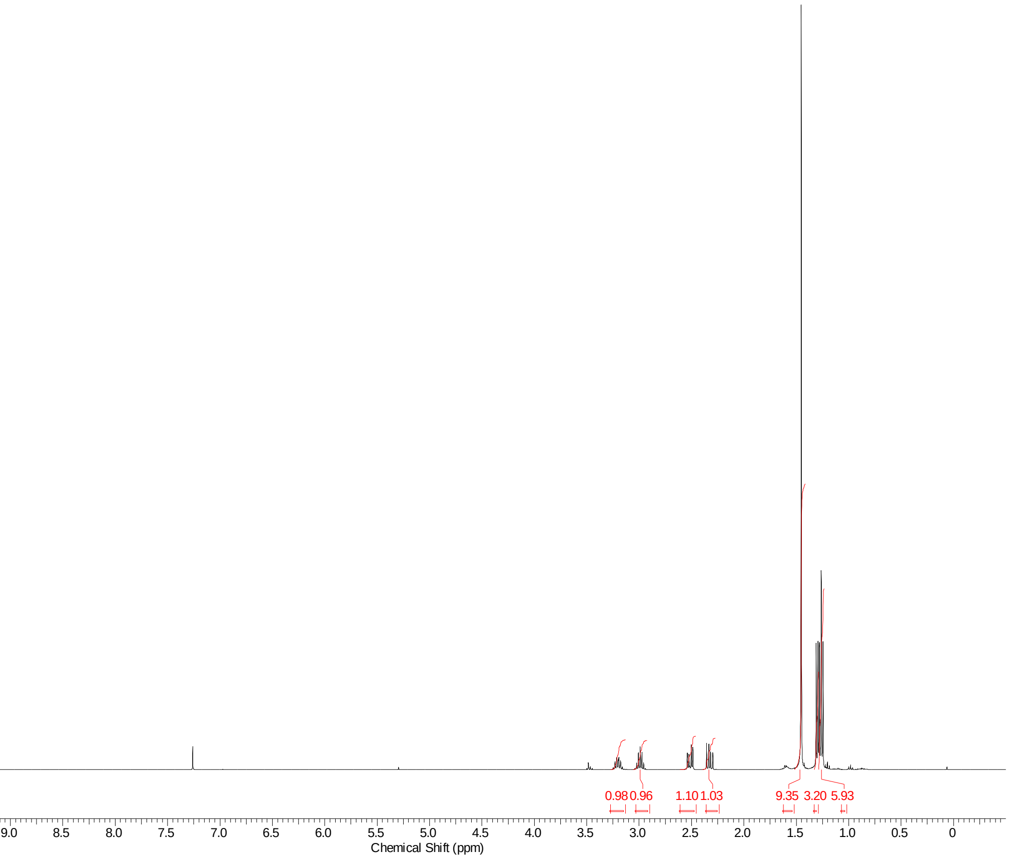

^{13}C NMR of **4g** (100 MHz, $CDCl_3$)

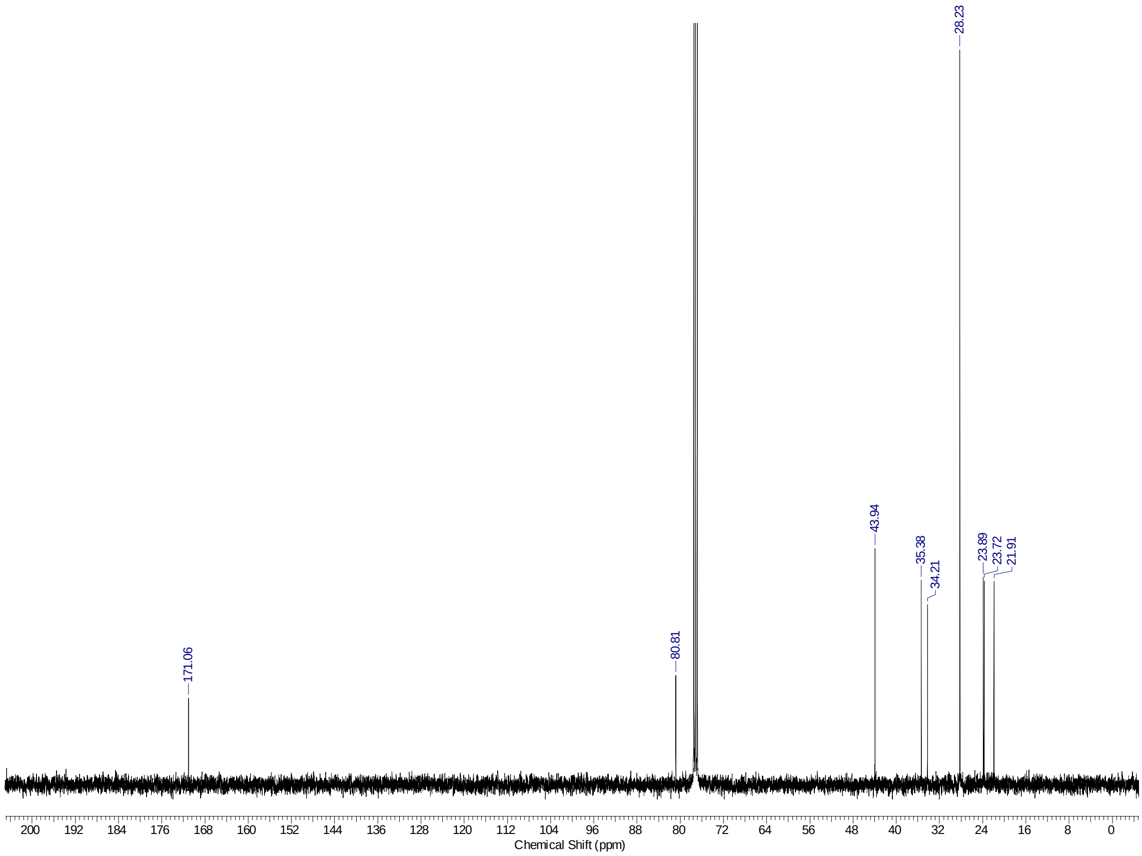

^{1}H NMR of **4h** (400 MHz, $CDCl_3$)

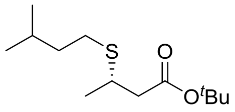

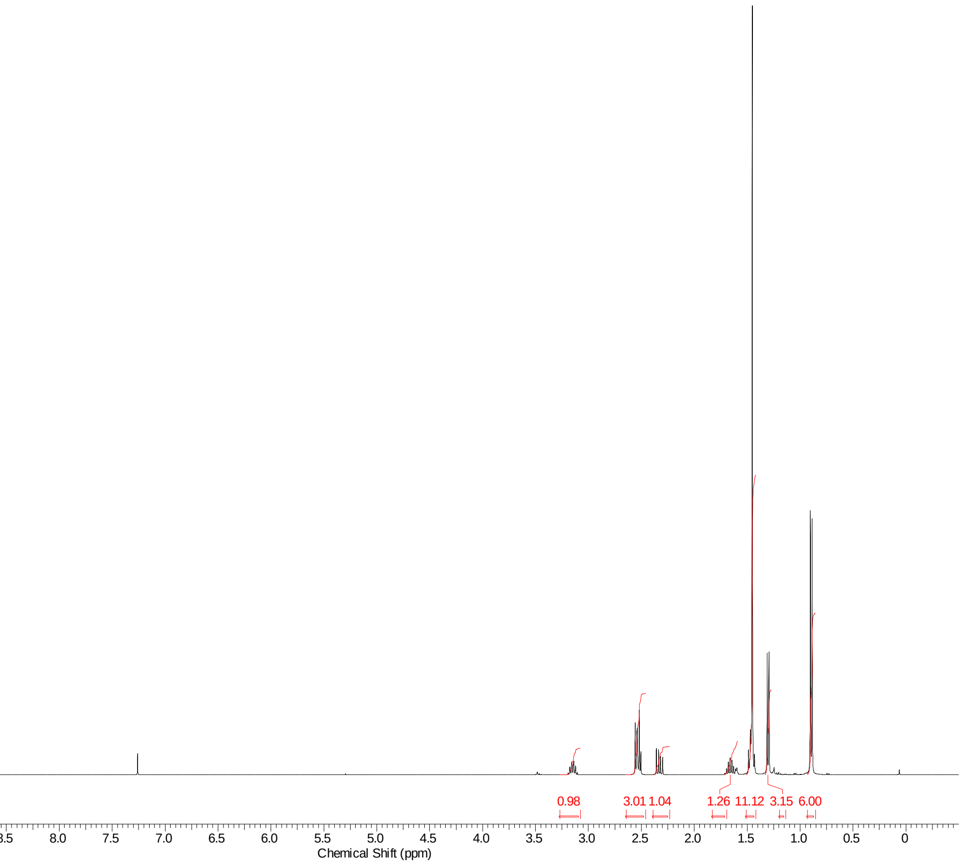

^{13}C NMR of **4h** (100 MHz)

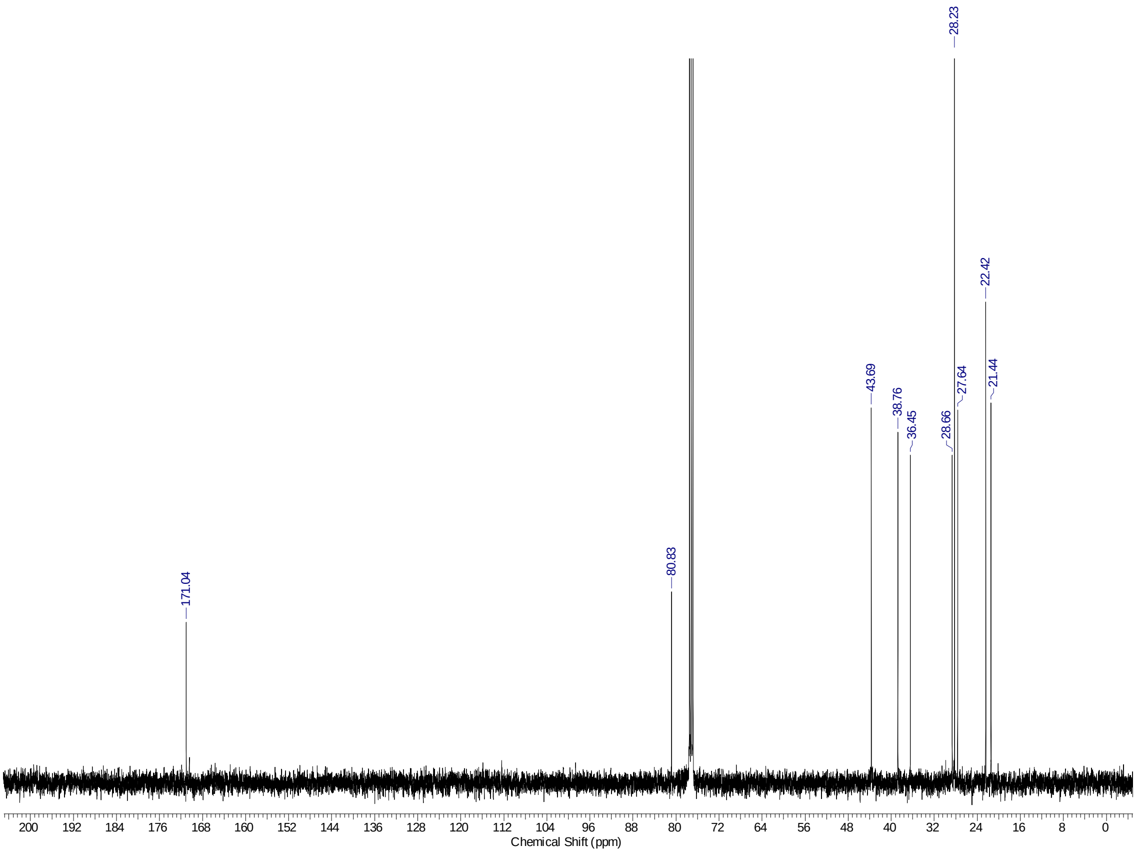

^{1}H NMR of **4i** (400 MHz, $CDCl_3$)

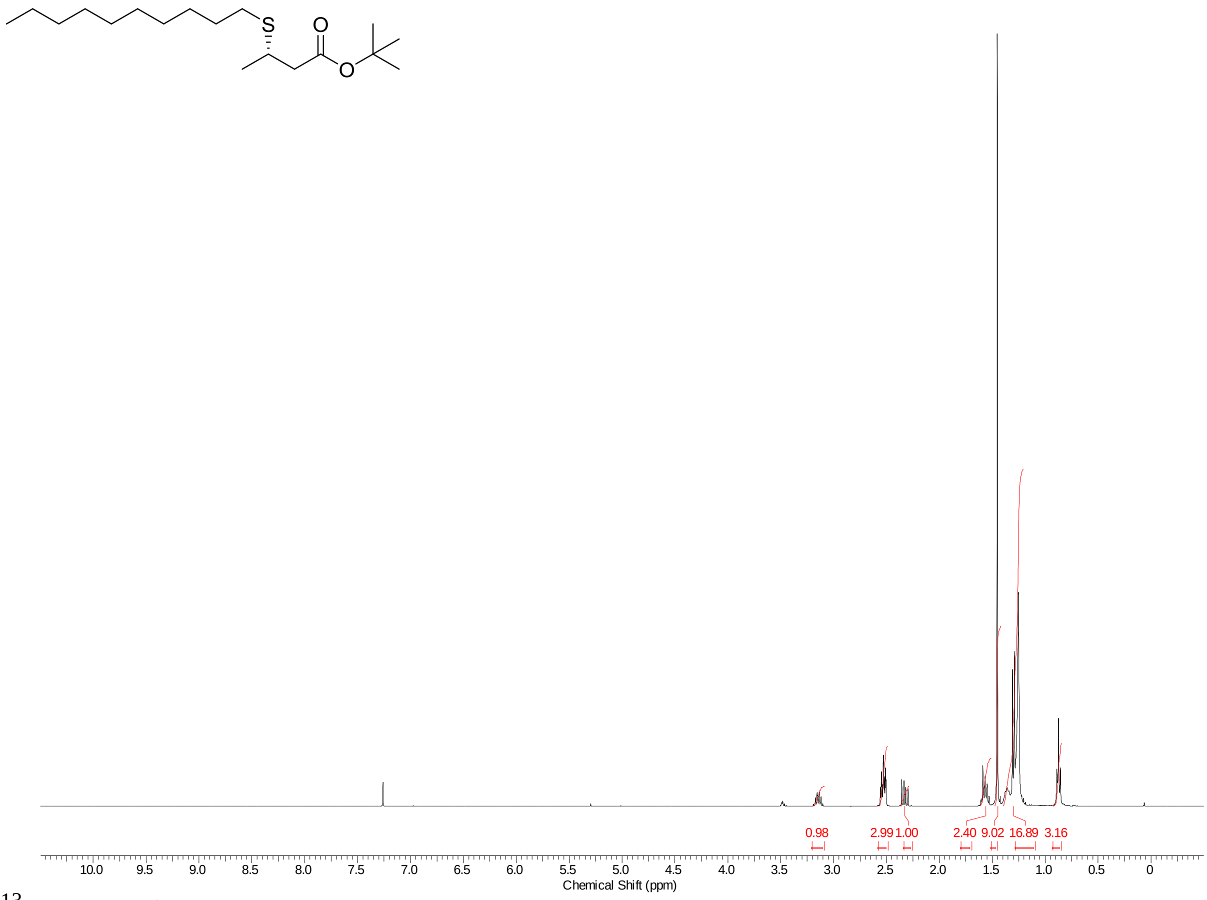

^{13}C NMR of **4i** (100 MHz, $CDCl_3$)

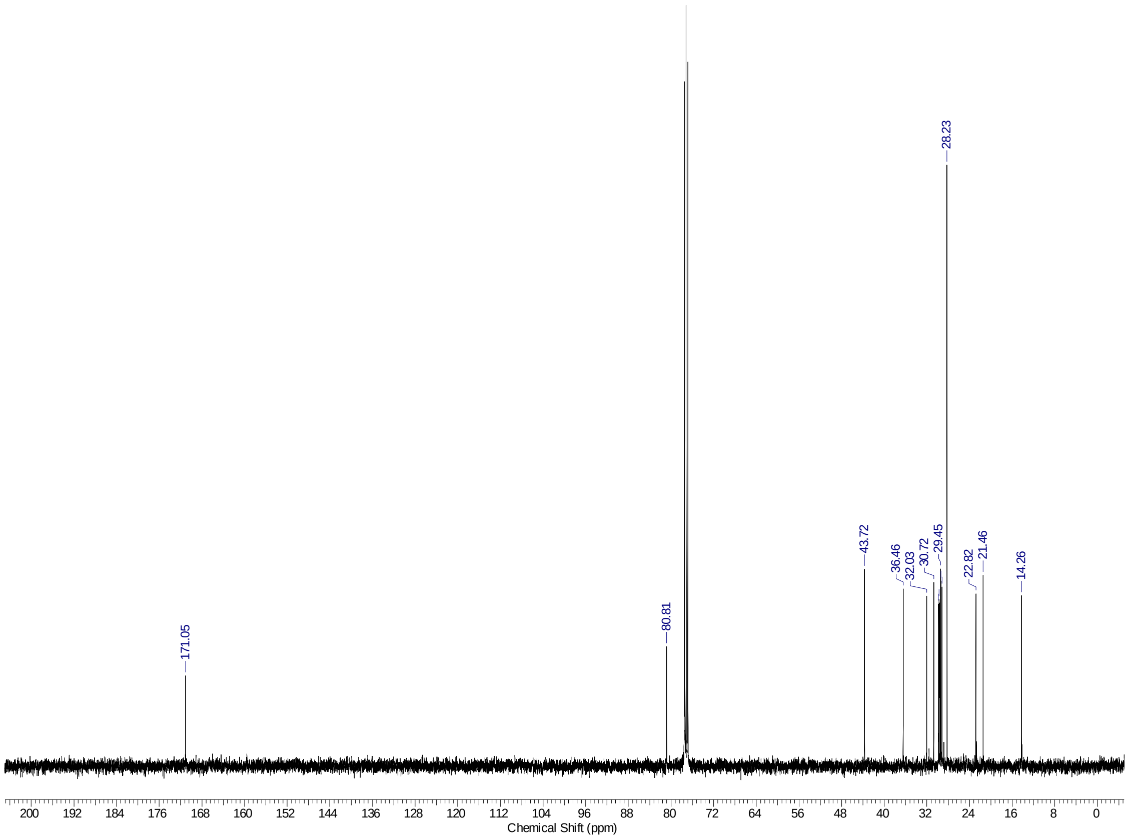

^{1}H NMR of **4j** (400 MHz, $CDCl_3$)

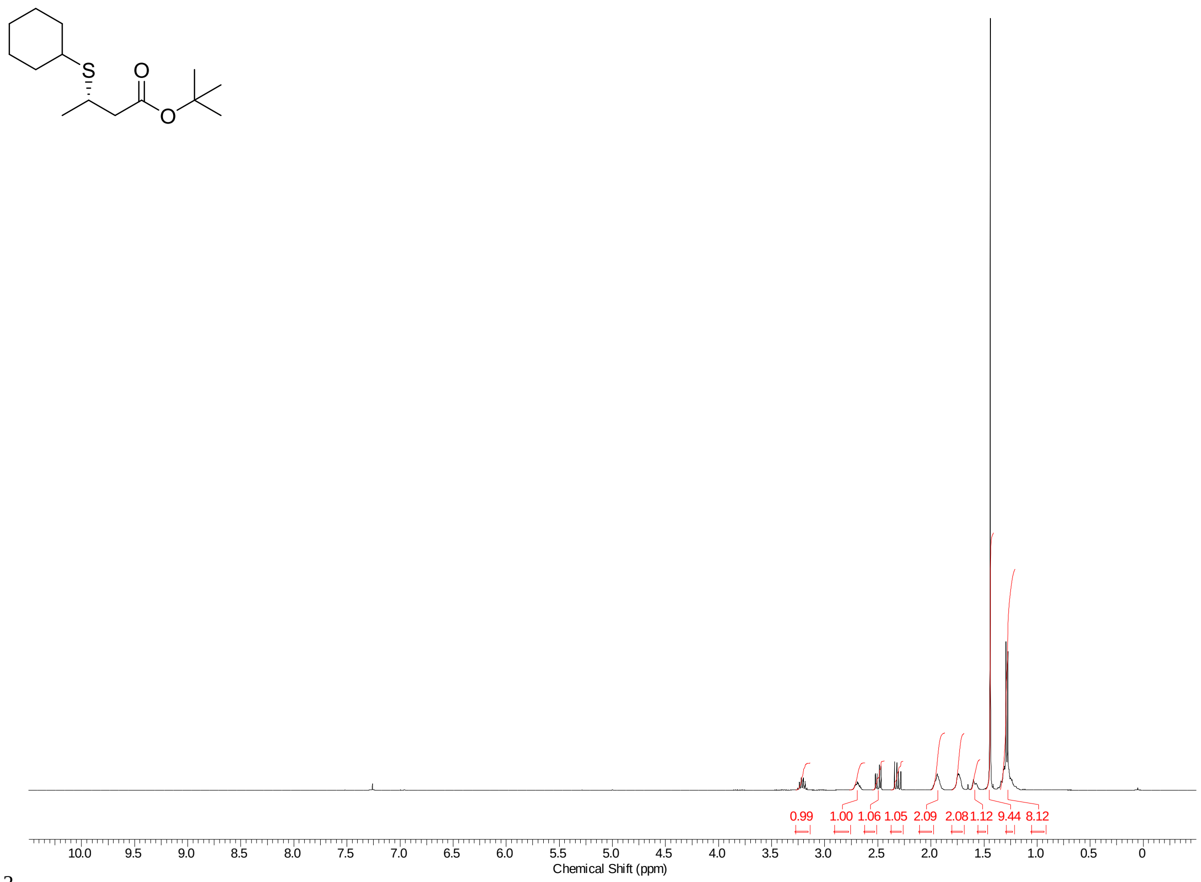

^{13}C NMR of **4j** (100 MHz, $CDCl_3$)

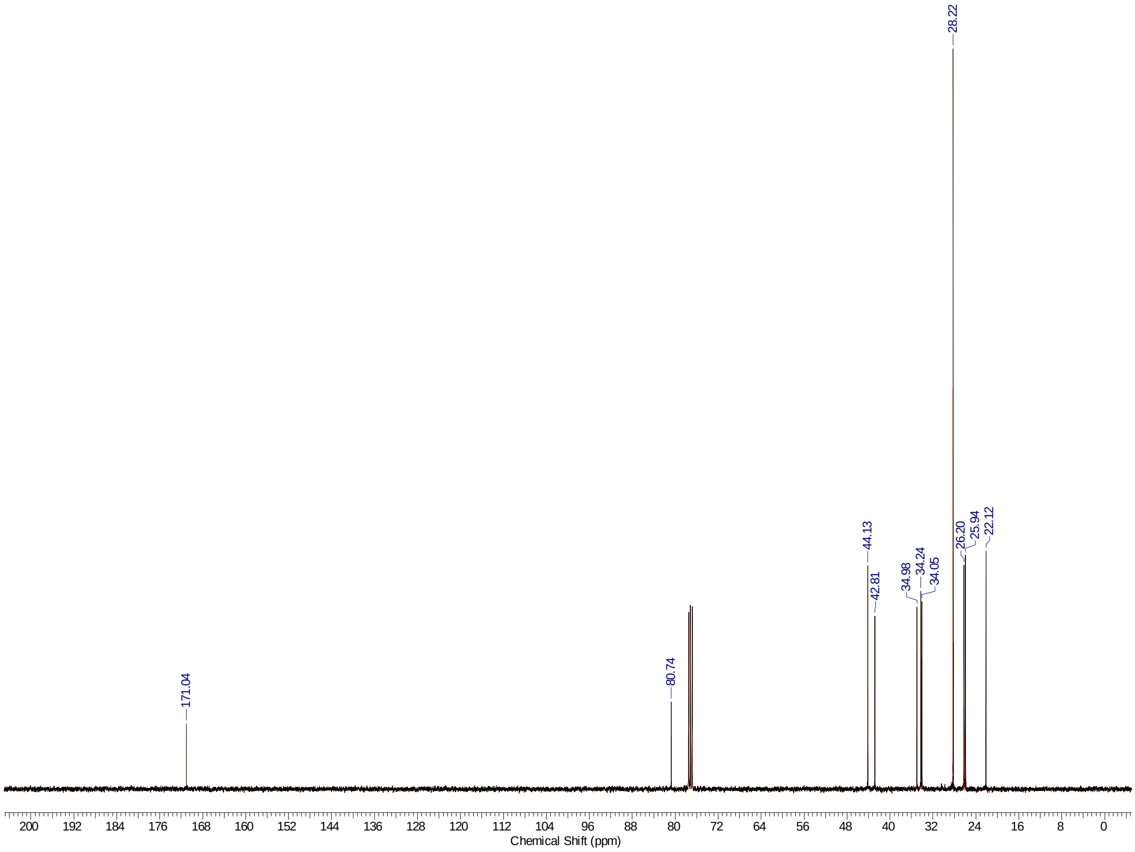

^{1}H NMR of **4k** (400 MHz, $CDCl_3$)

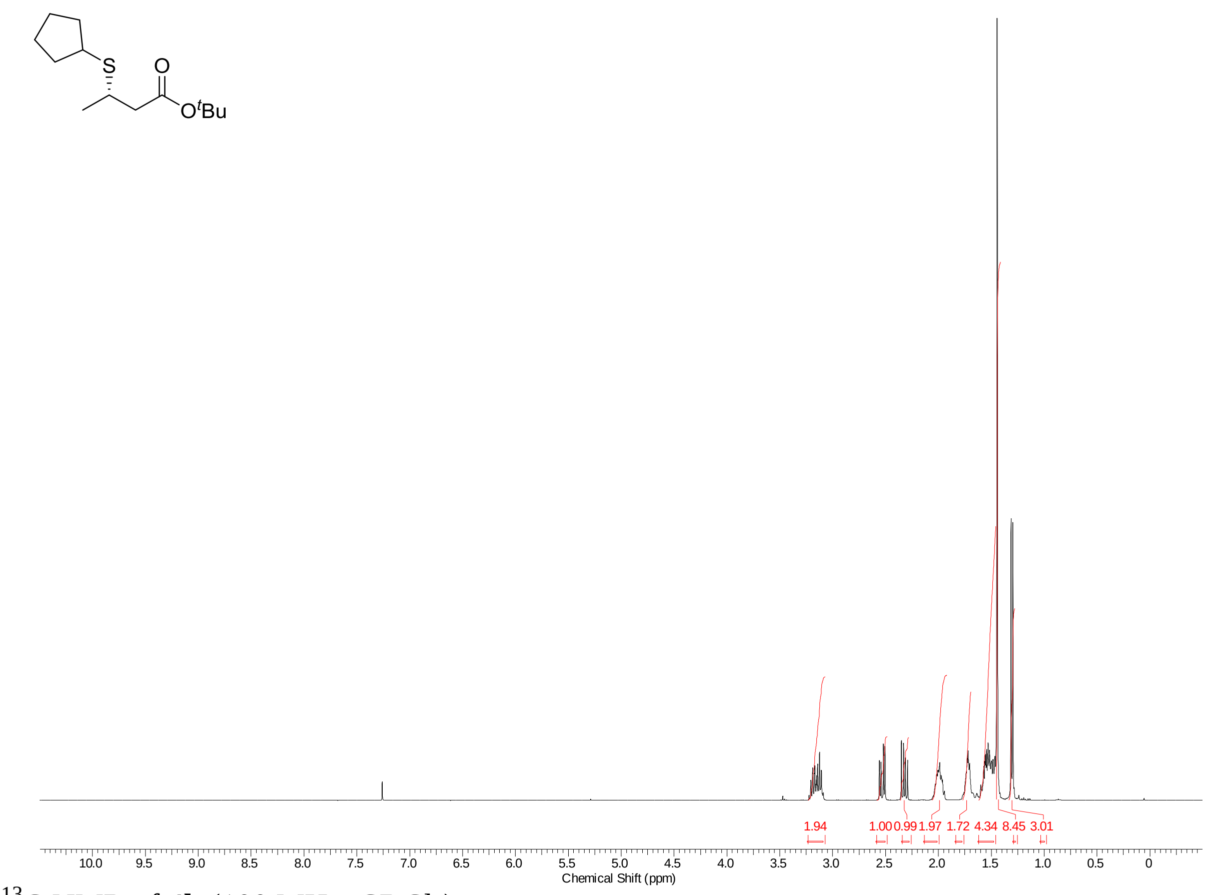

^{13}C NMR of **4k** (100 MHz, $CDCl_3$)

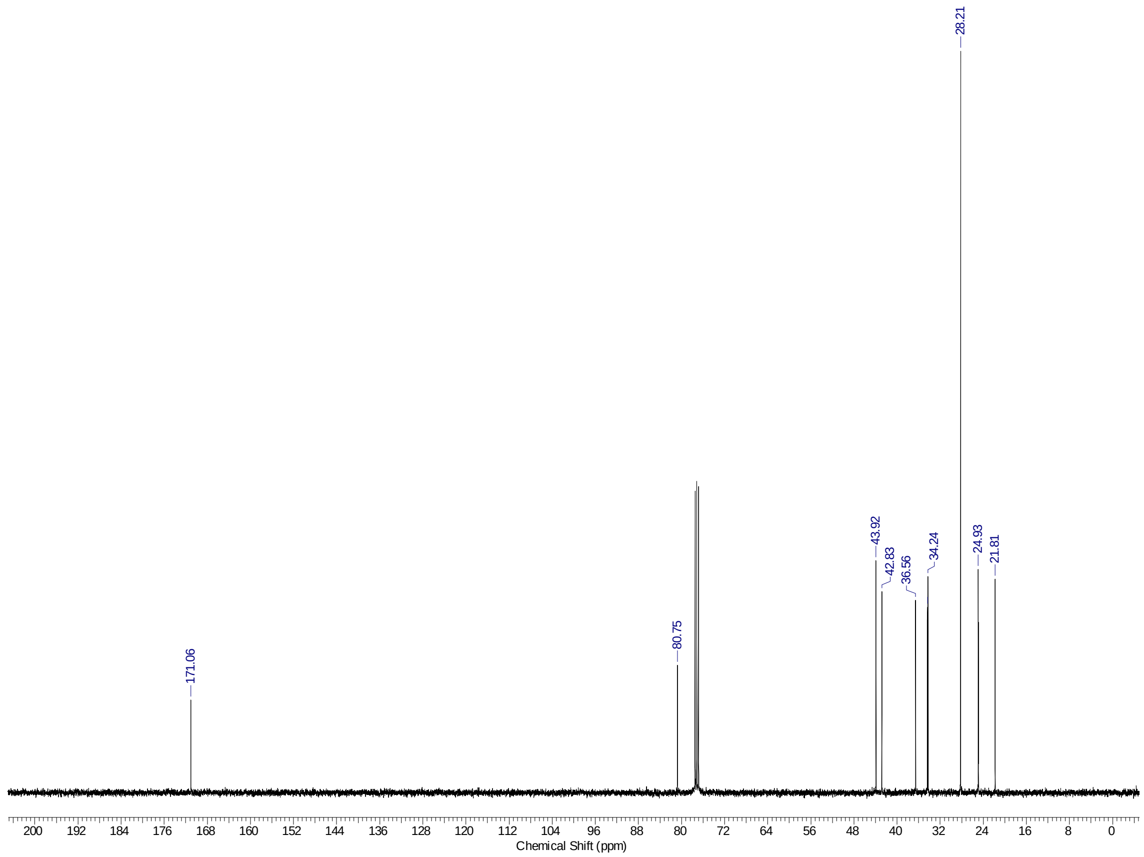

^{1}H NMR of **4l** (400 MHz, $CDCl_3$)

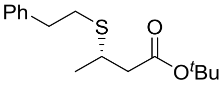

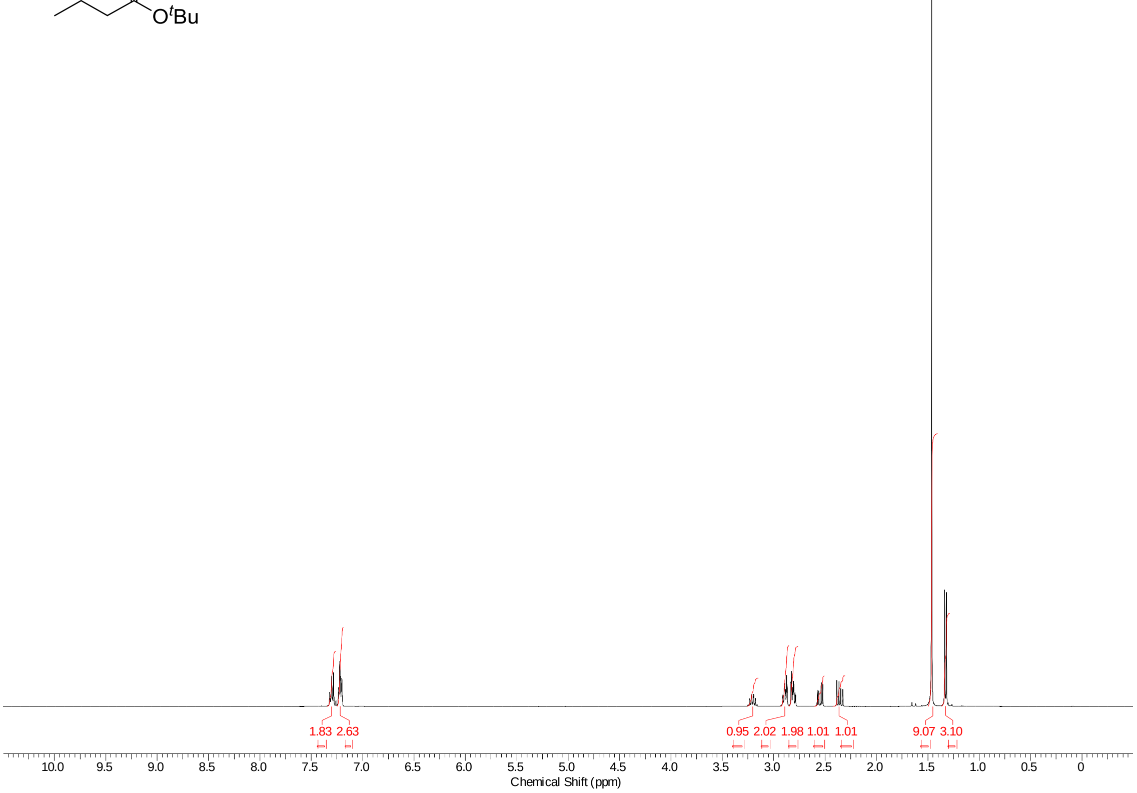

^{13}C NMR of **4l** (100 MHz, $CDCl_3$)

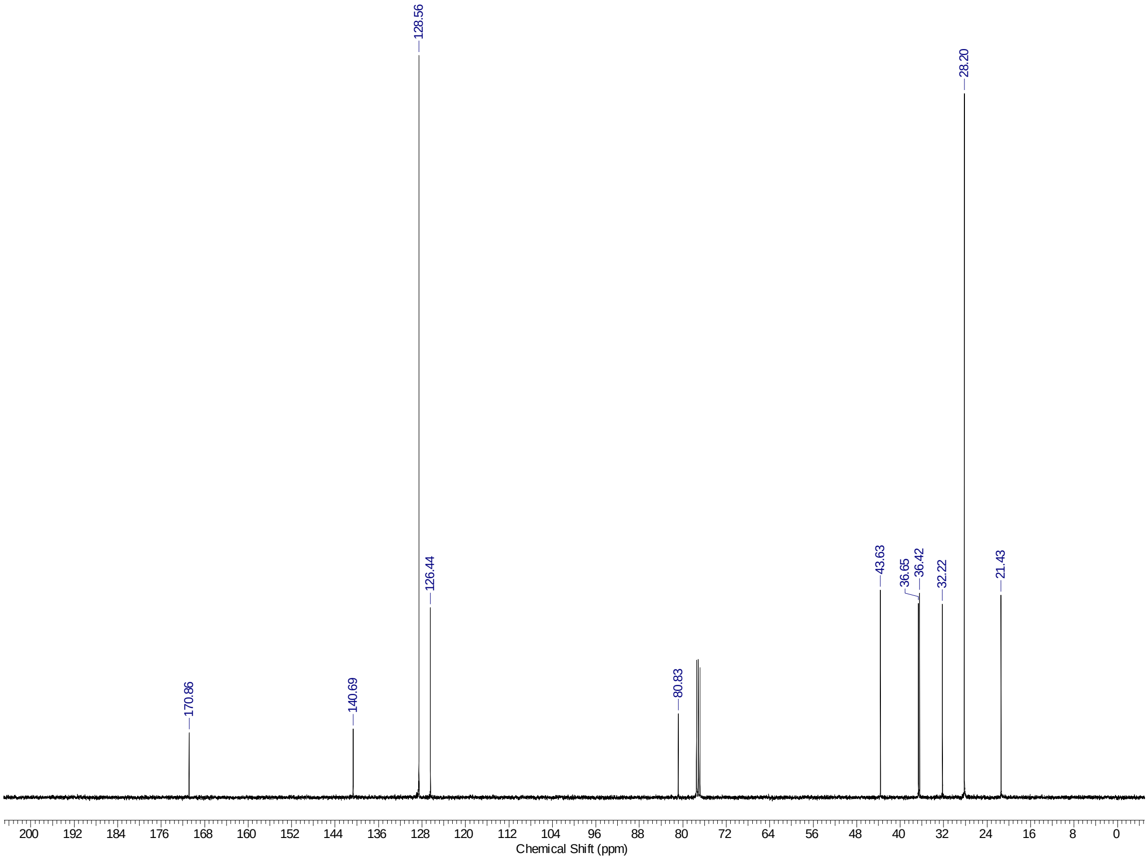

^{1}H NMR of **4m** (400 MHz, $CDCl_3$)

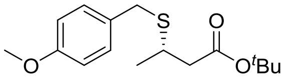

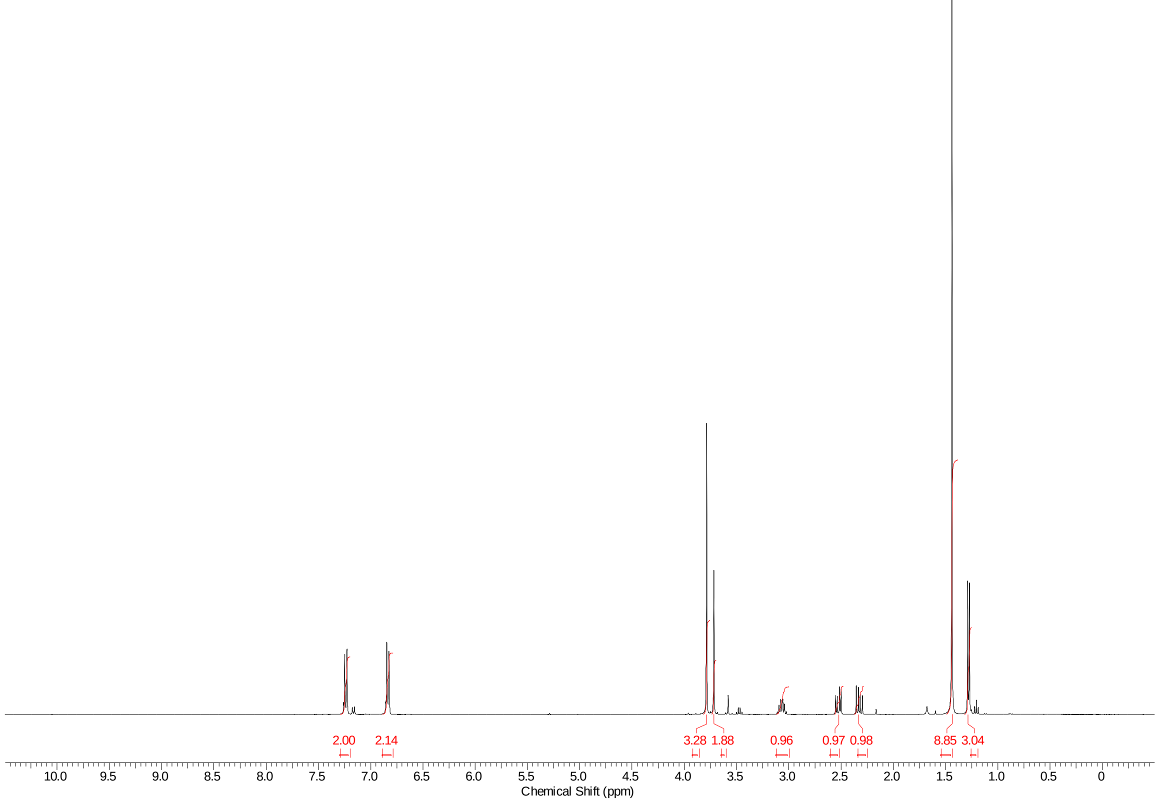

^{13}C NMR of **4m** (100 MHz, $CDCl_3$)

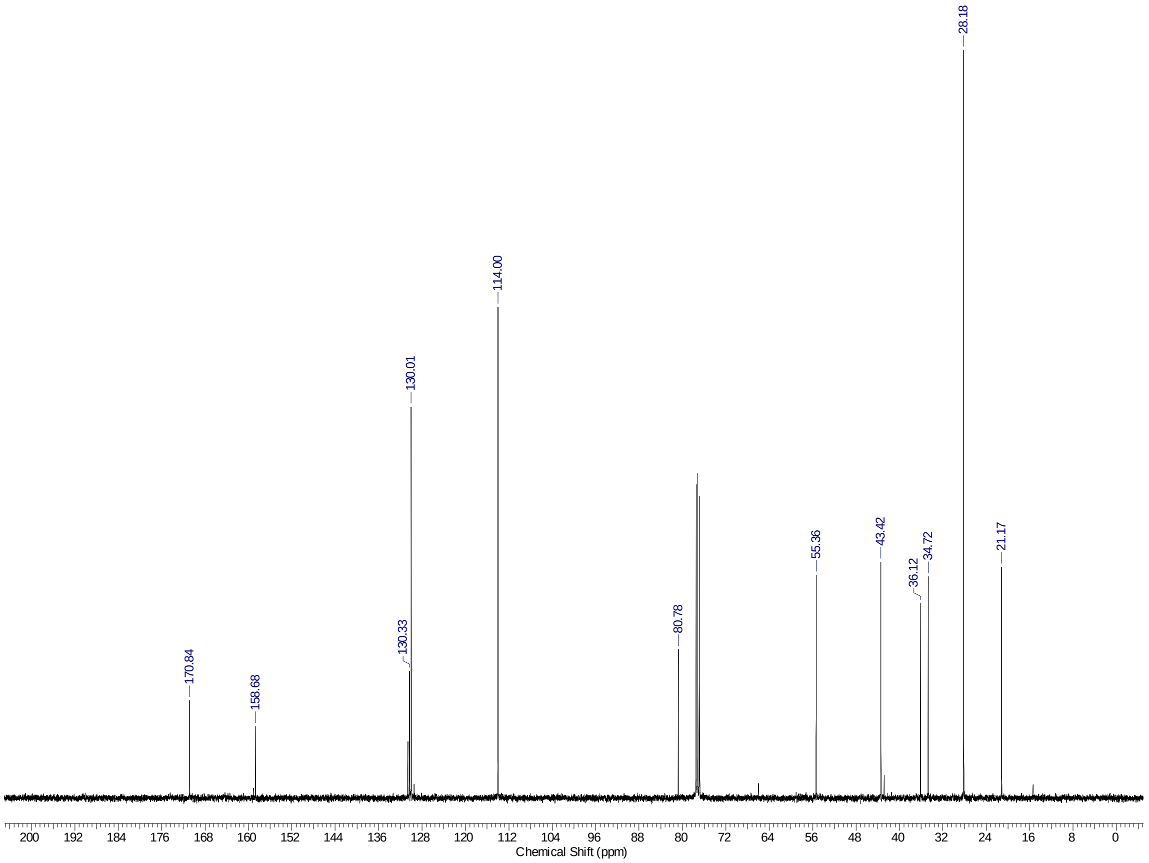

^{1}H NMR of **4n** (400 MHz, $CDCl_3$)

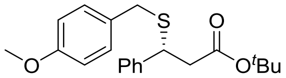

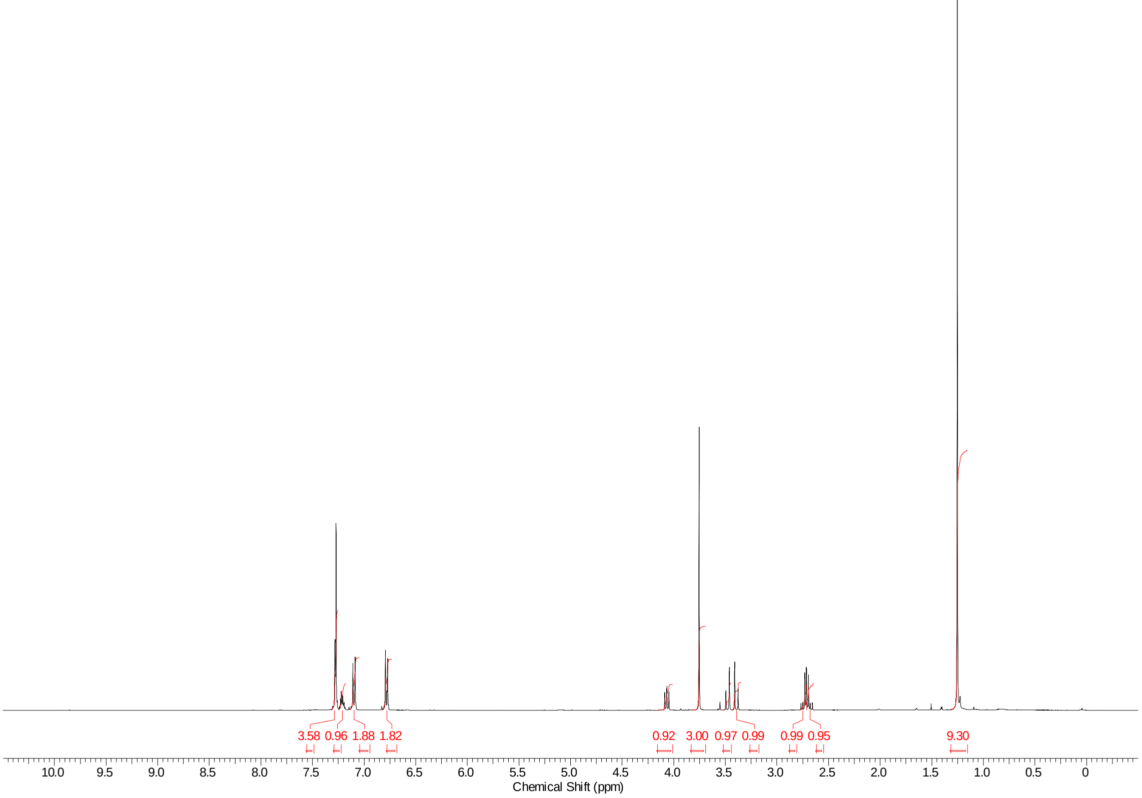

^{13}C NMR of **4n** (100 MHz, $CDCl_3$)

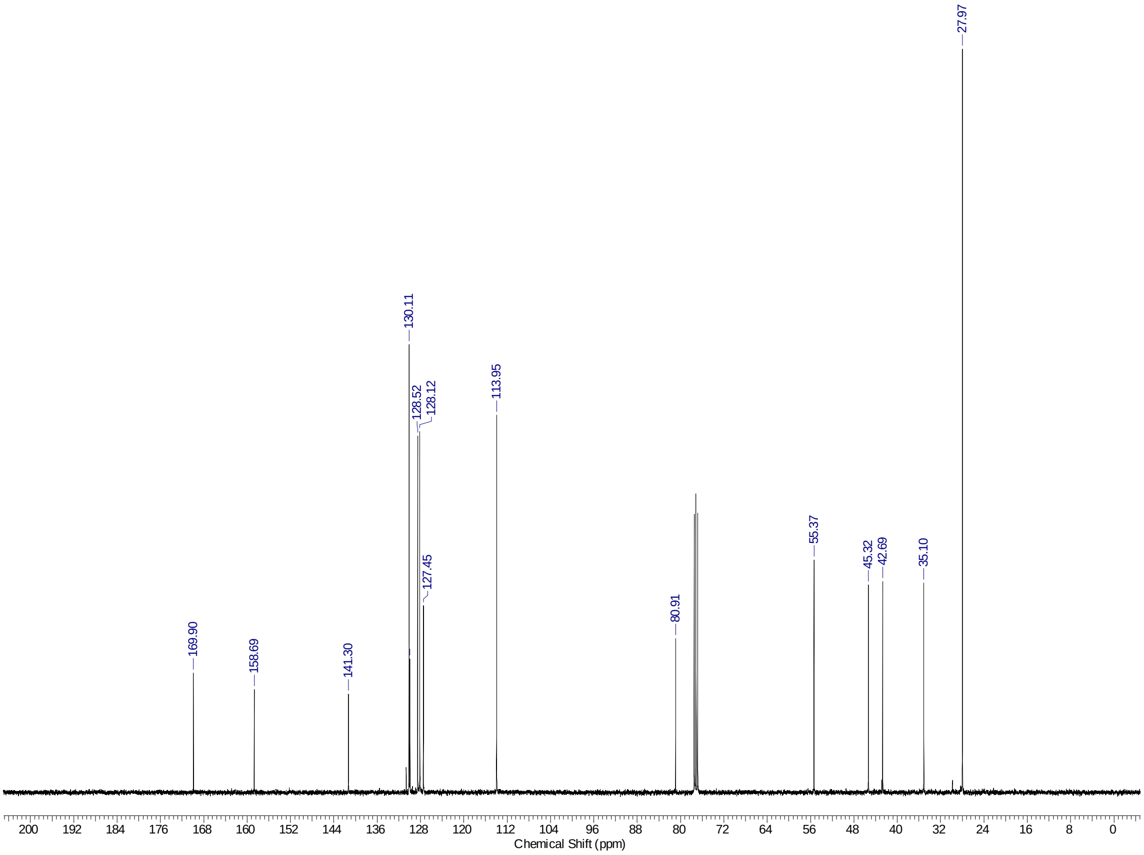

^{1}H NMR of **4o** (400 MHz, $CDCl_3$)

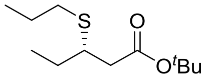

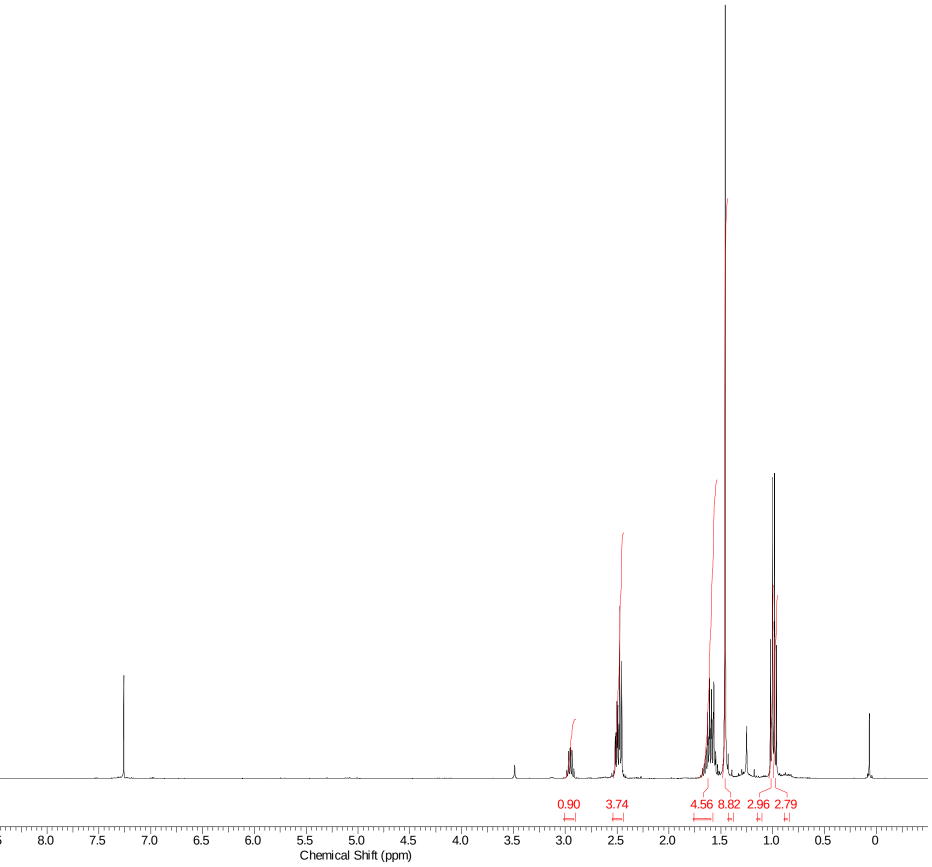

^{13}C NMR of **4o** (100 MHz, $CDCl_3$)

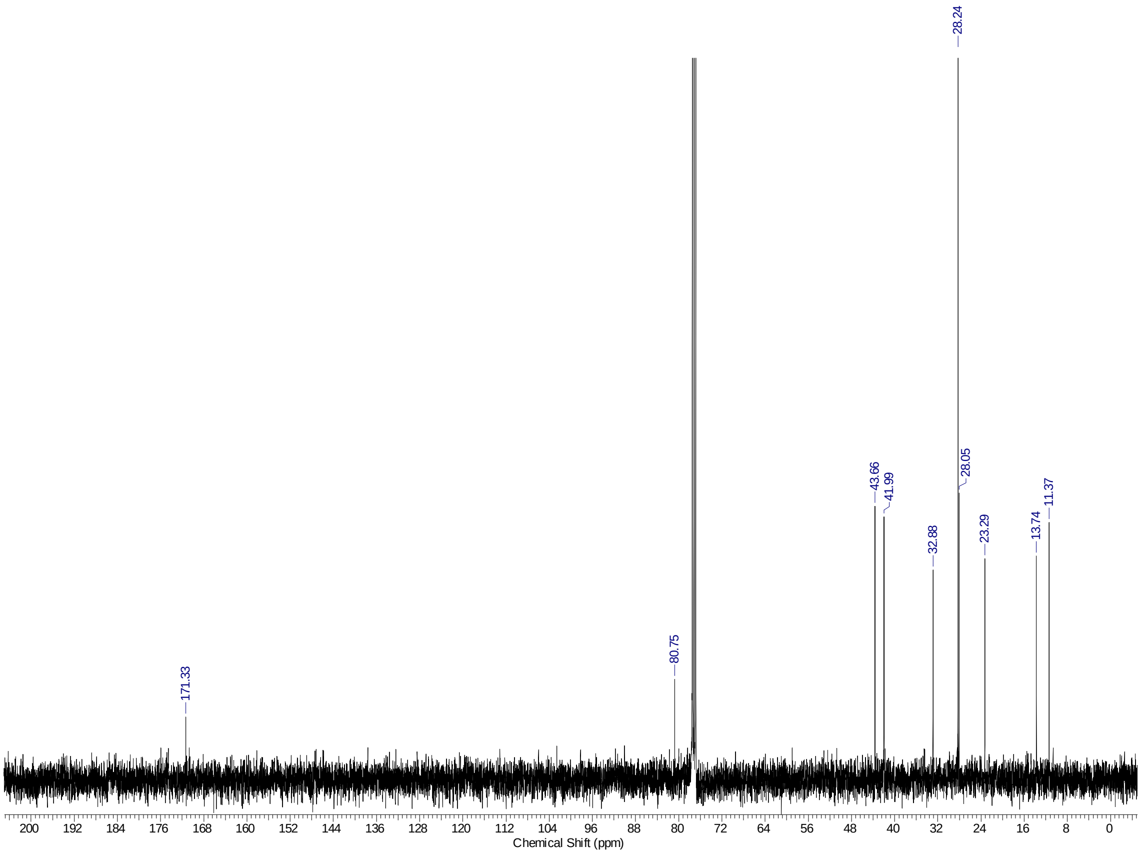

^{1}H NMR of **4p** (400 MHz, $CDCl_3$)

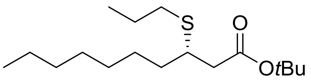

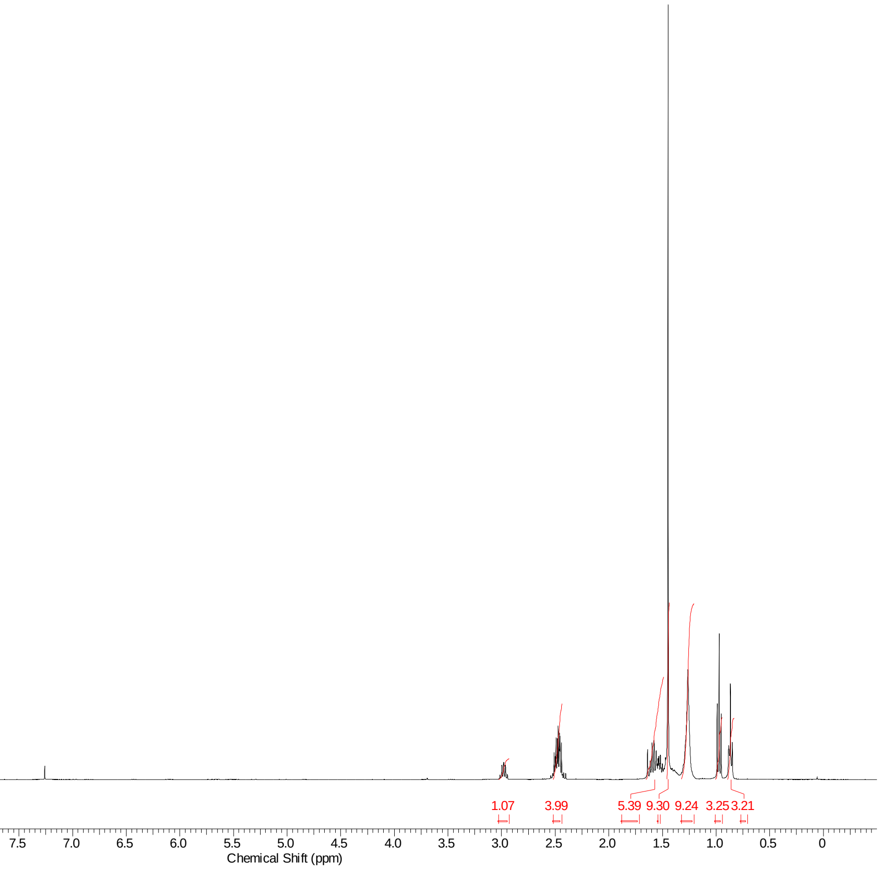

^{13}C NMR of **4p** (100 MHz, $CDCl_3$)

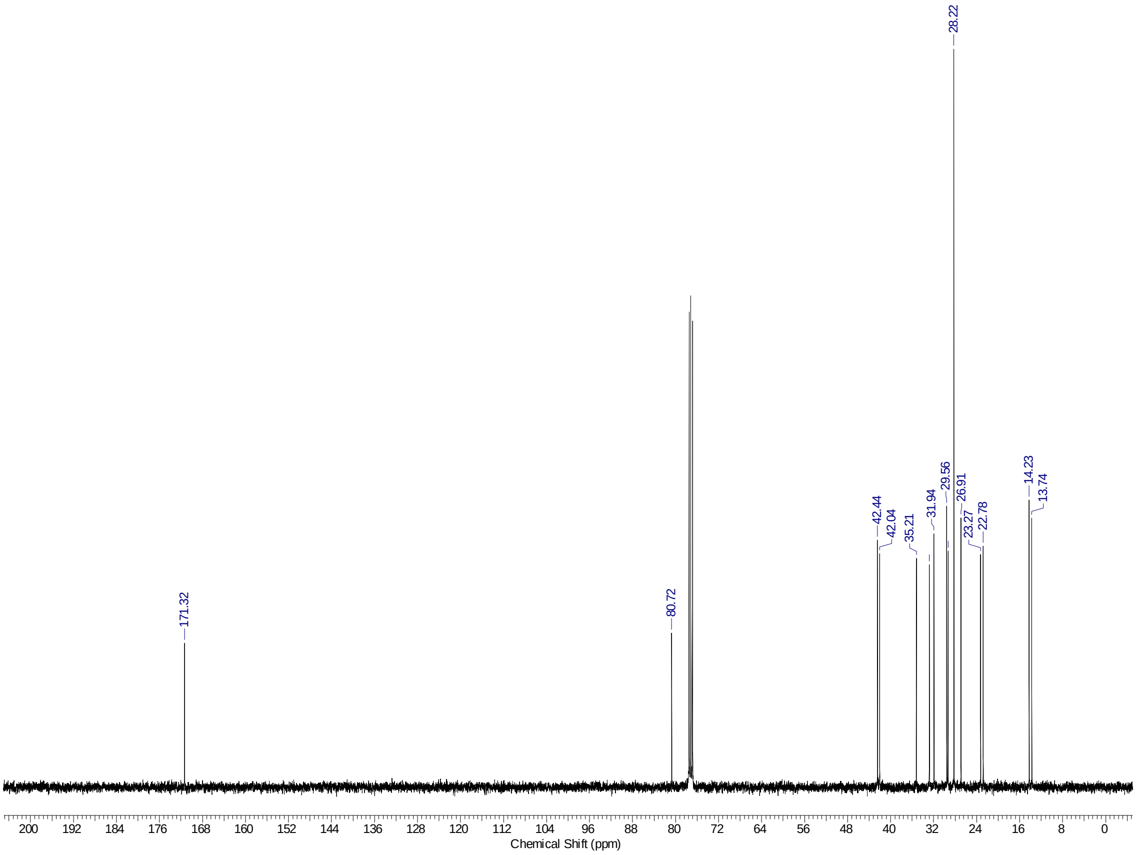

^{1}H NMR of **4q** (400 MHz, $CDCl_3$)

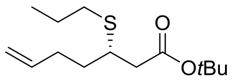

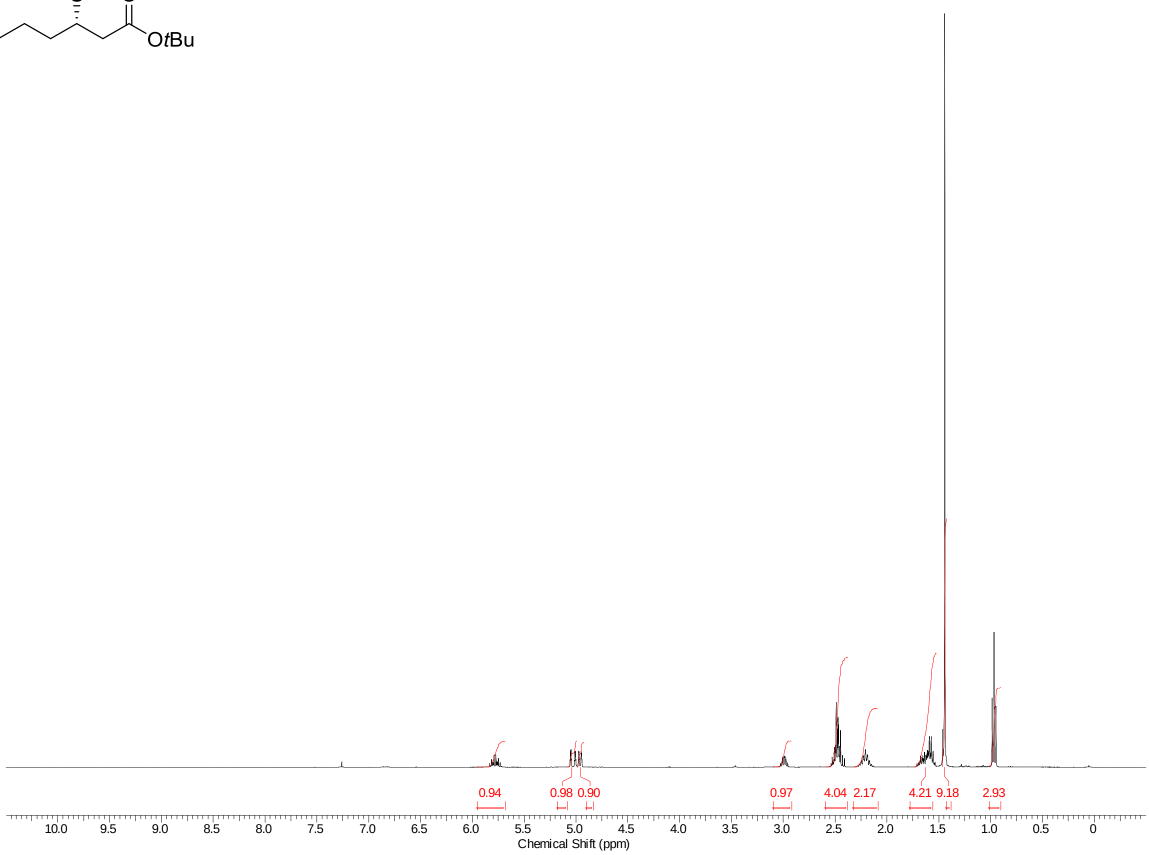

^{13}C NMR of **4q** (100 MHz, $CDCl_3$)

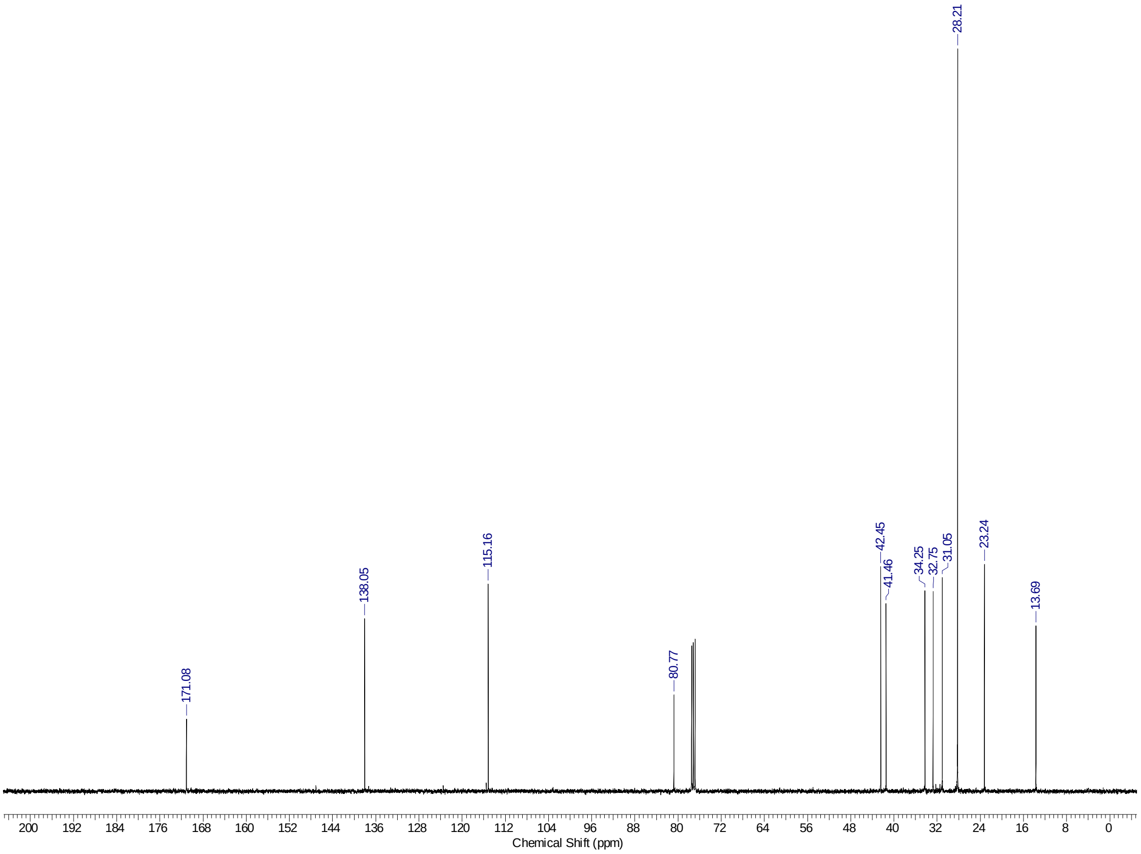

^{1}H NMR of **4r** (400 MHz, $CDCl_3$)

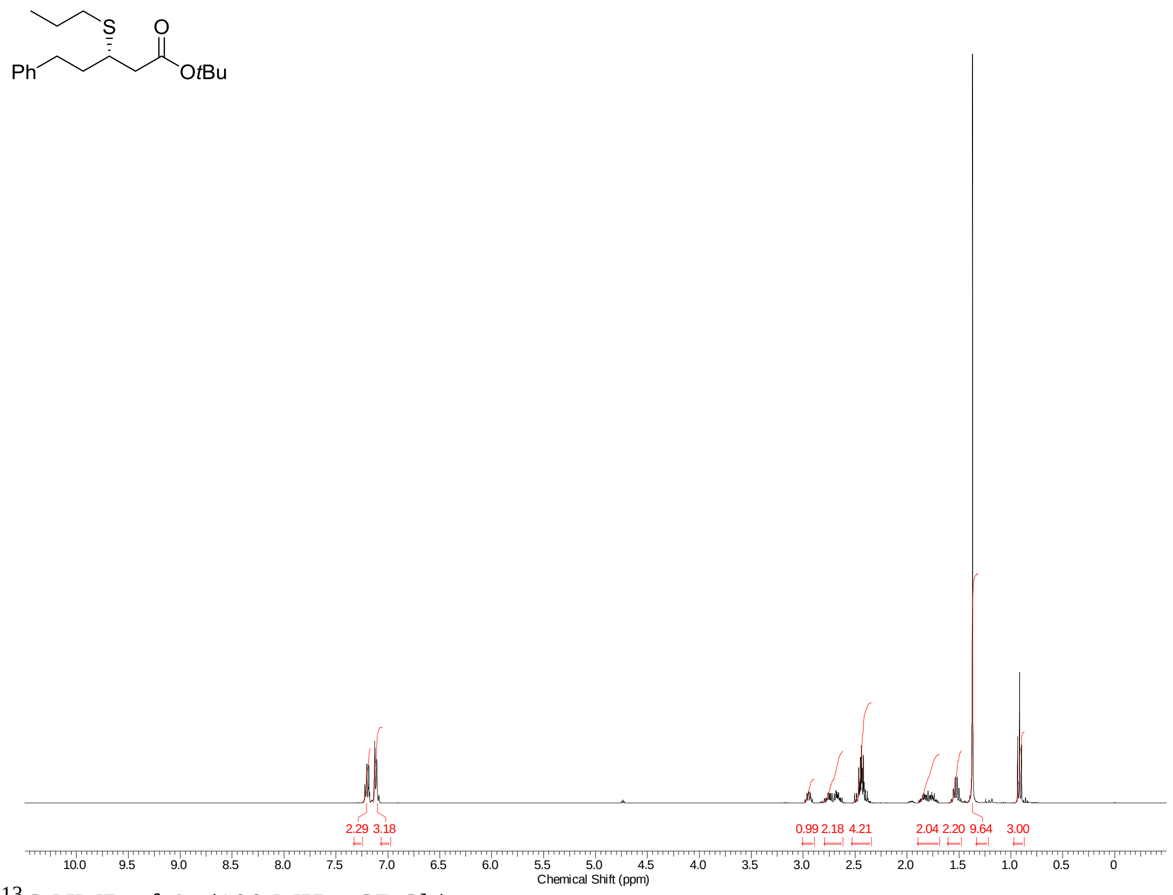

^{13}C NMR of **4r** (100 MHz, $CDCl_3$)

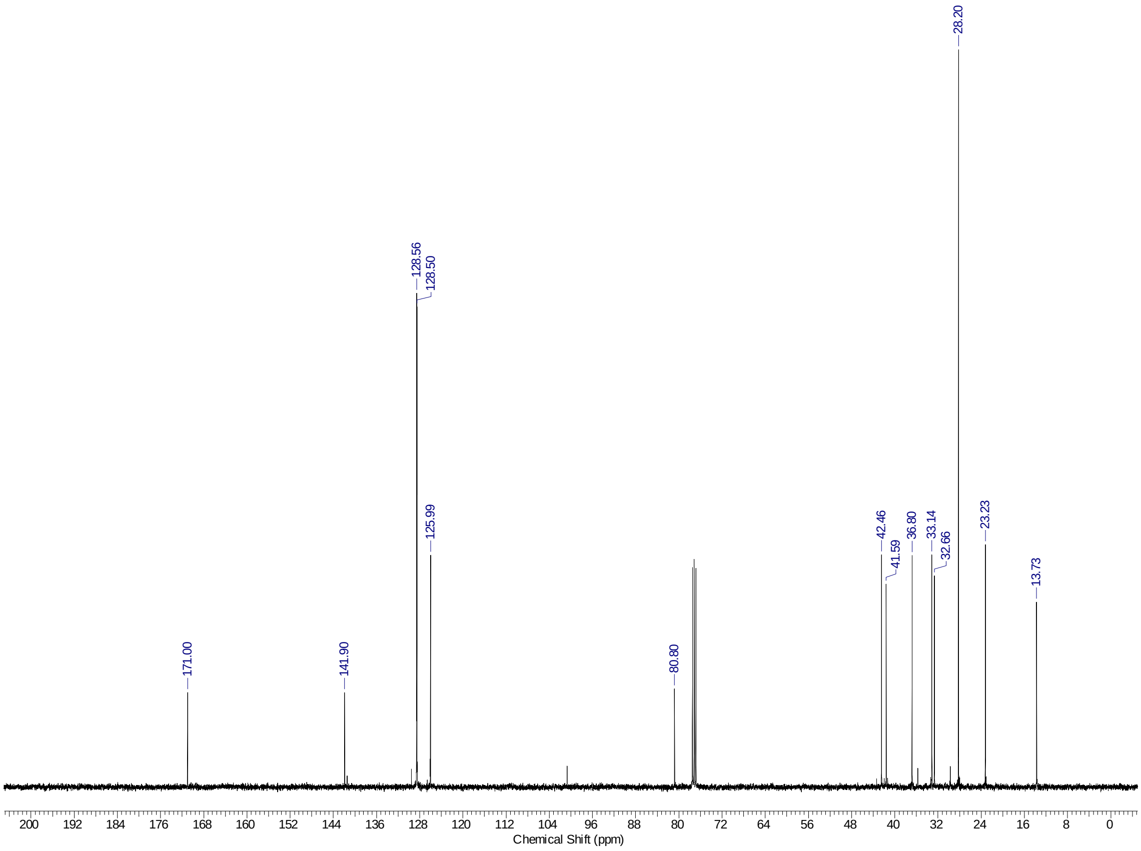

^{1}H NMR of **4s** (400 MHz, $CDCl_3$)

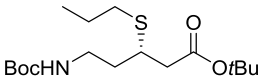

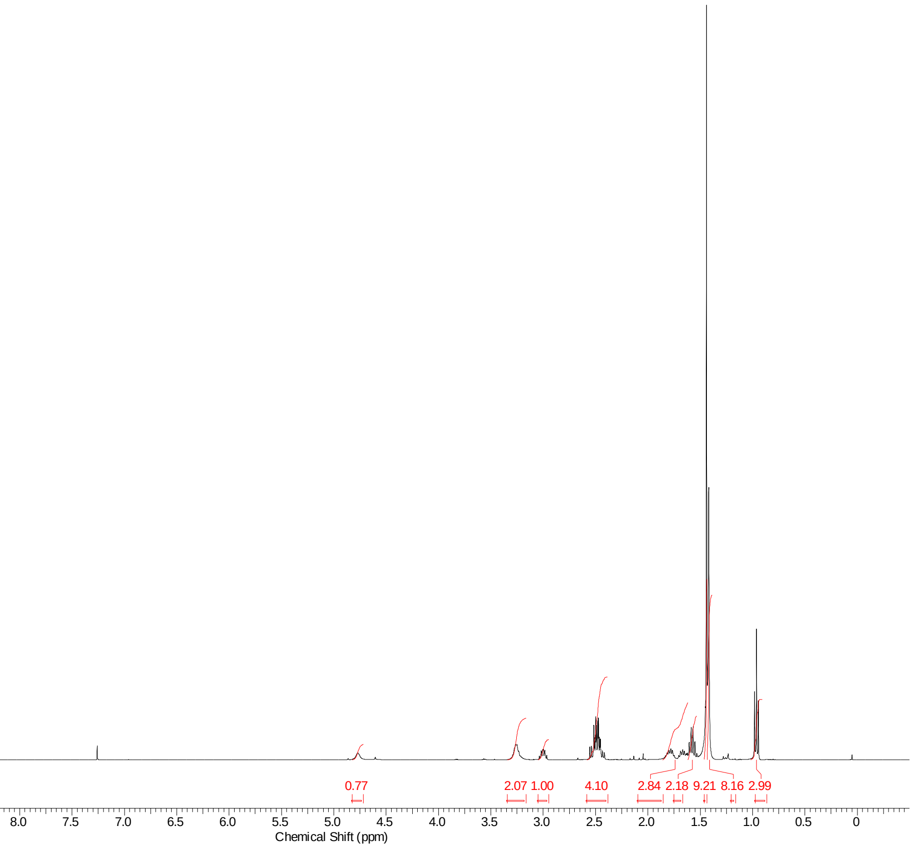

^{13}C NMR of **4s** (100 MHz, $CDCl_3$)

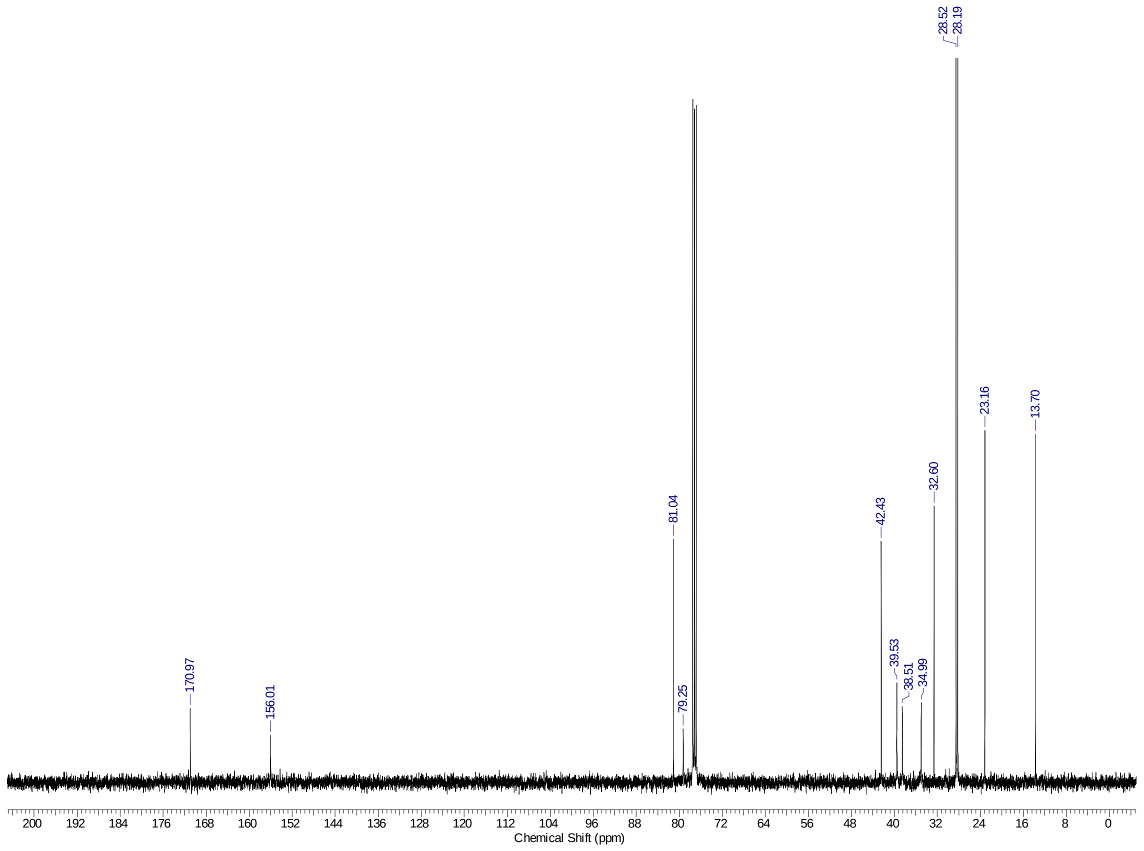

^{1}H NMR of **4t** (400 MHz, $CDCl_3$)

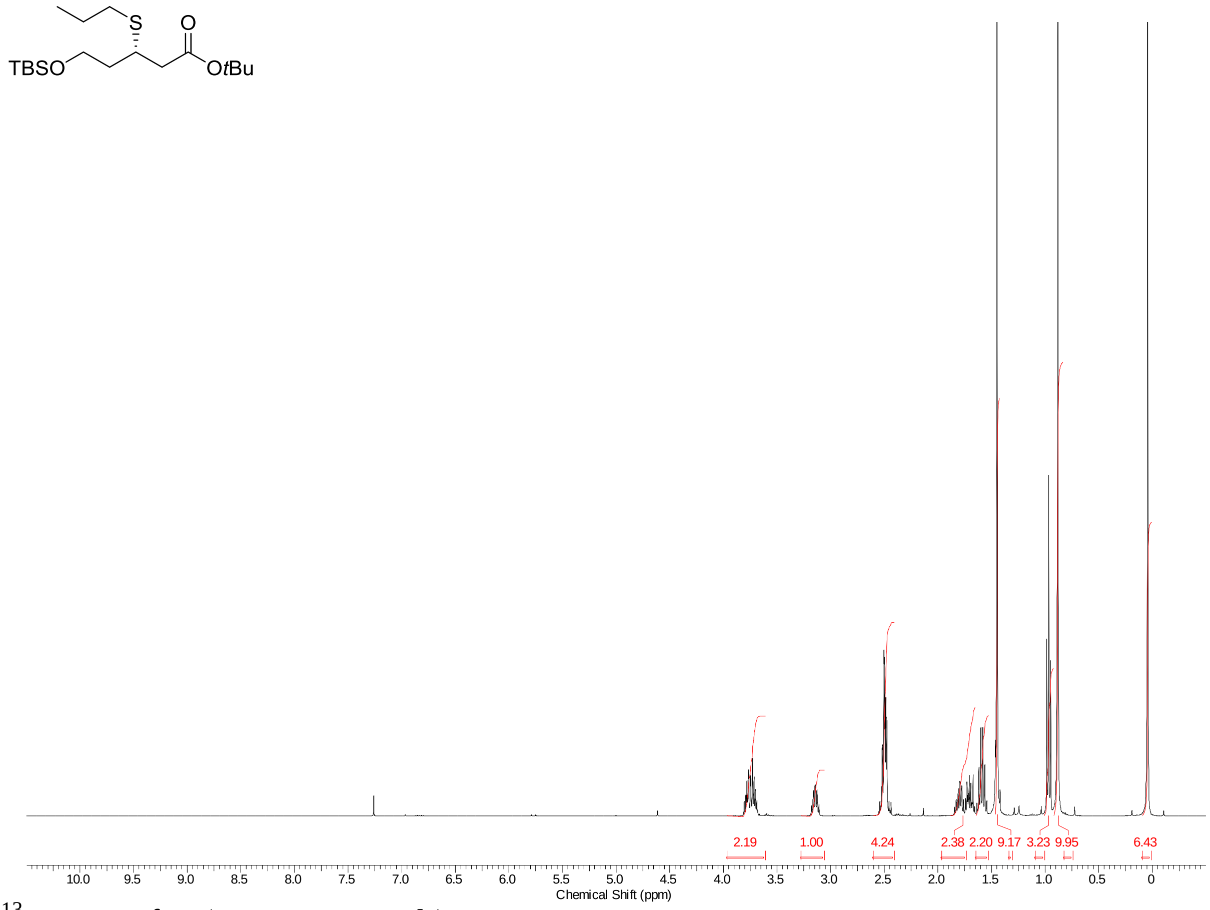

^{13}C NMR of **4t** (100 MHz, $CDCl_3$)

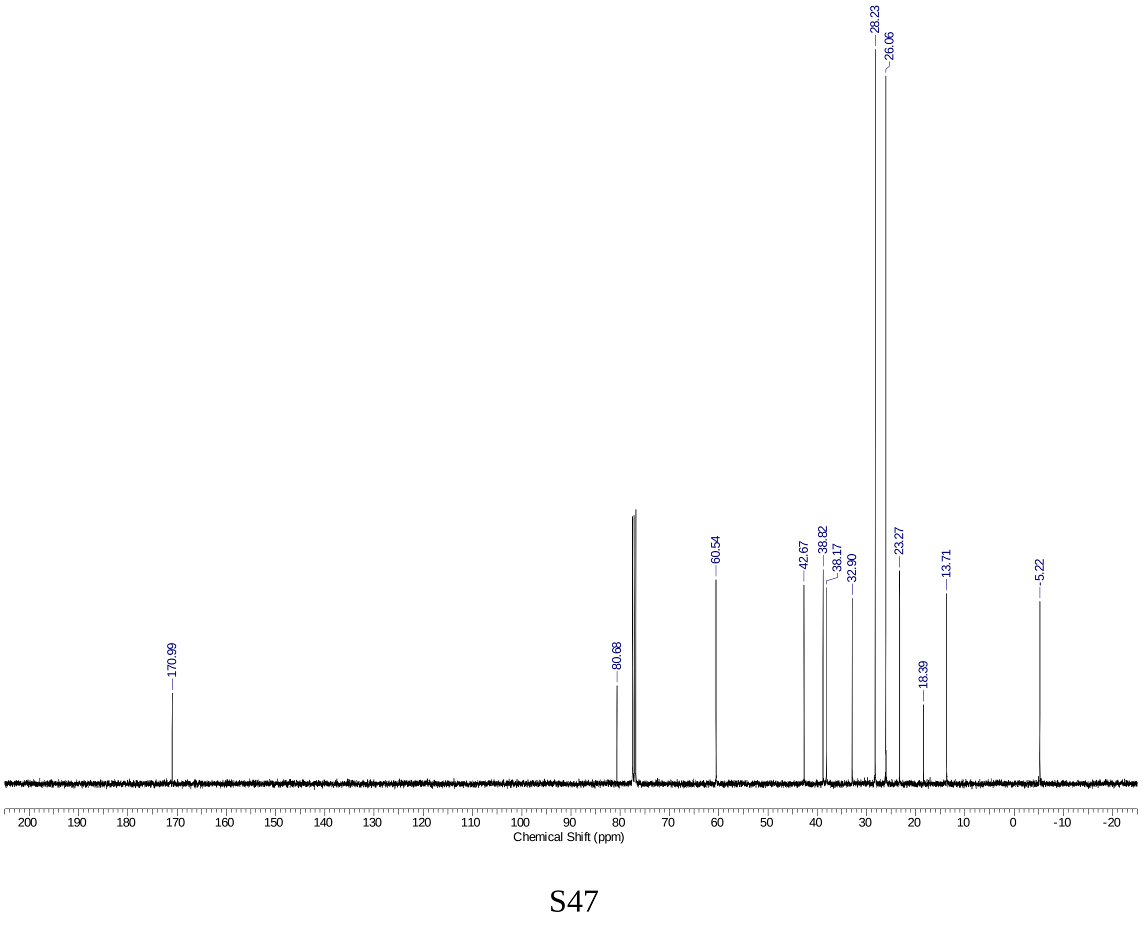

^{1}H NMR of **5a** (400 MHz, $CDCl_3$)

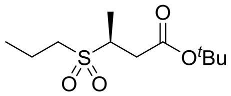

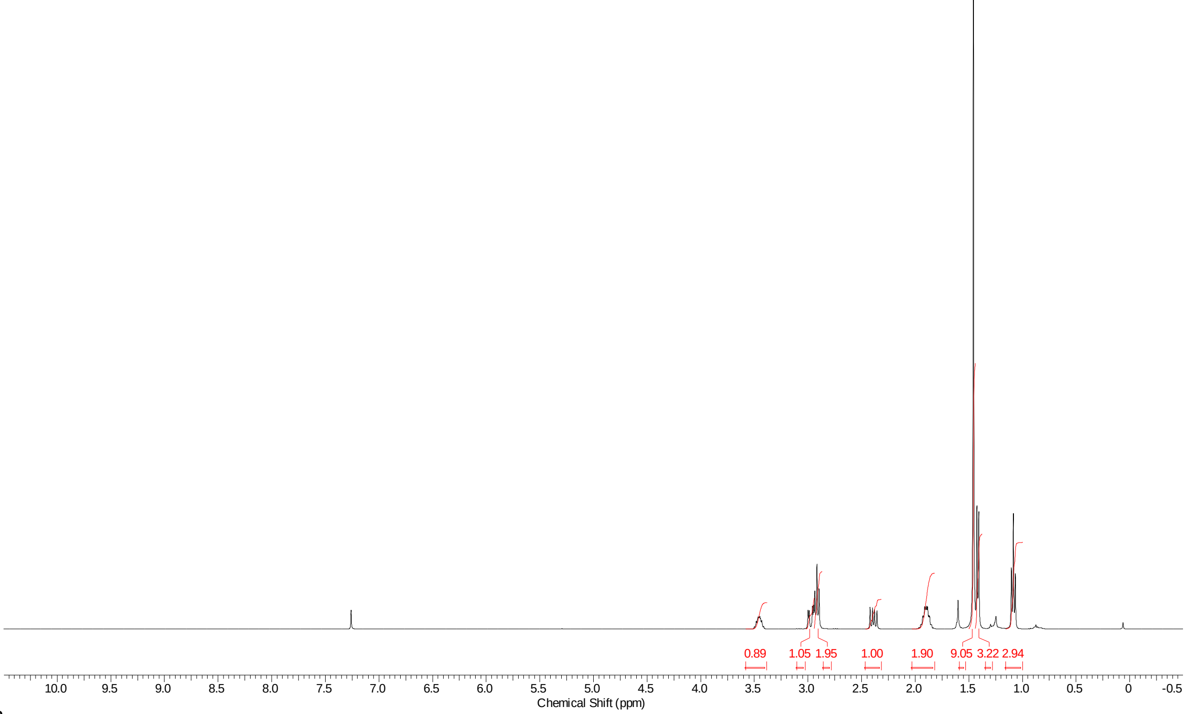

^{13}C NMR of **5a** (100 MHz, $CDCl_3$)

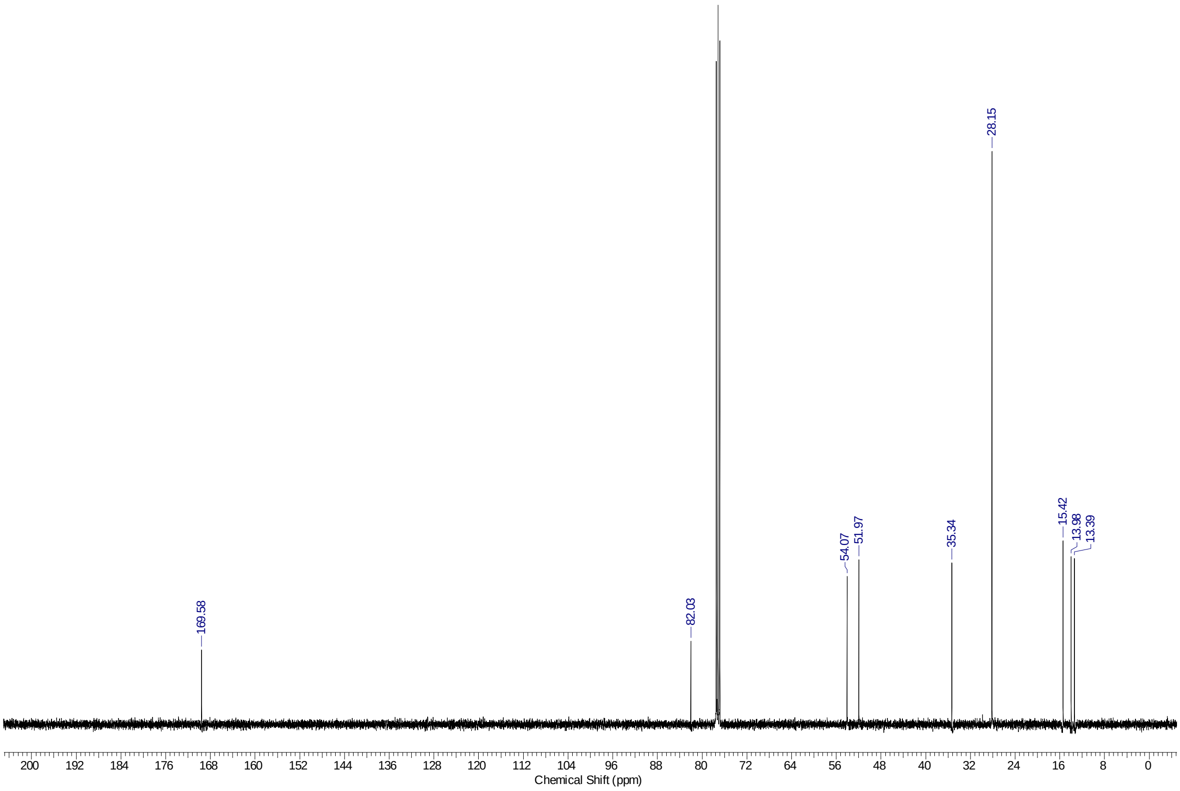

^{1}H NMR of **5b** (400 MHz, $CDCl_3$)

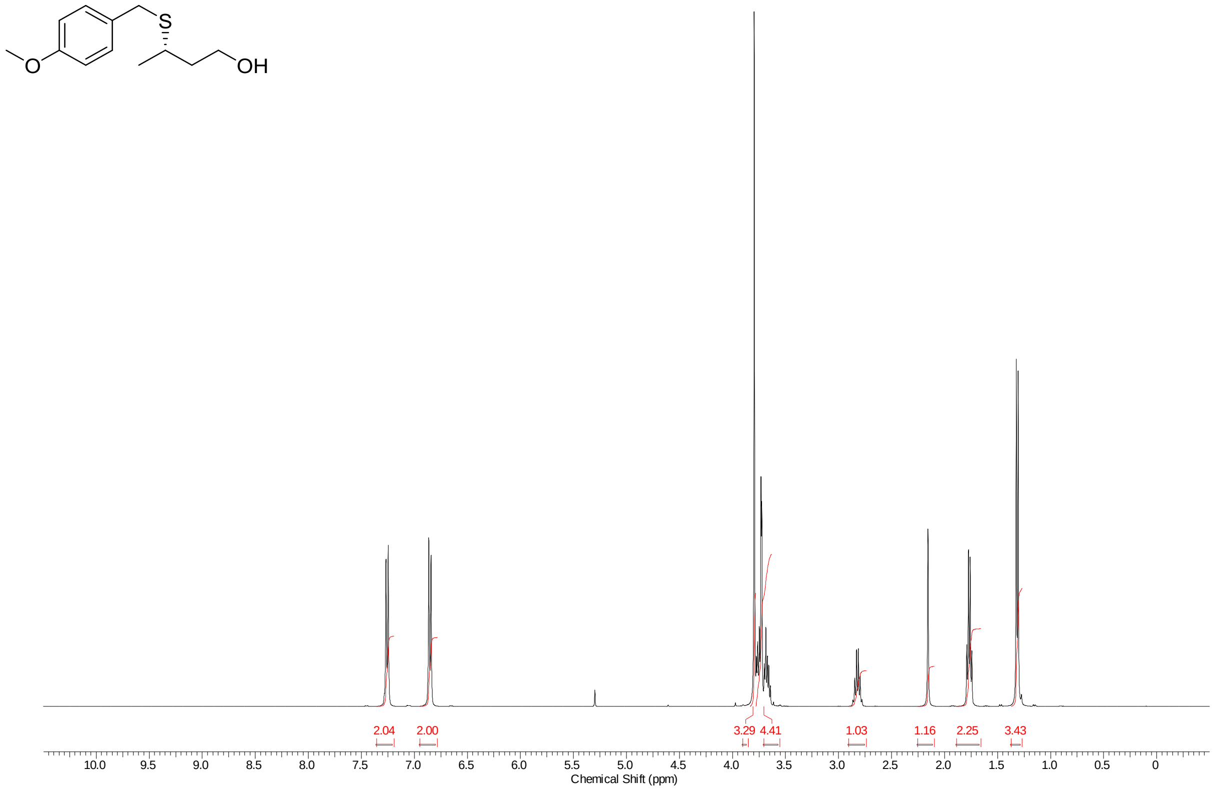

^{13}C NMR of **5b** (100 MHz, $CDCl_3$)

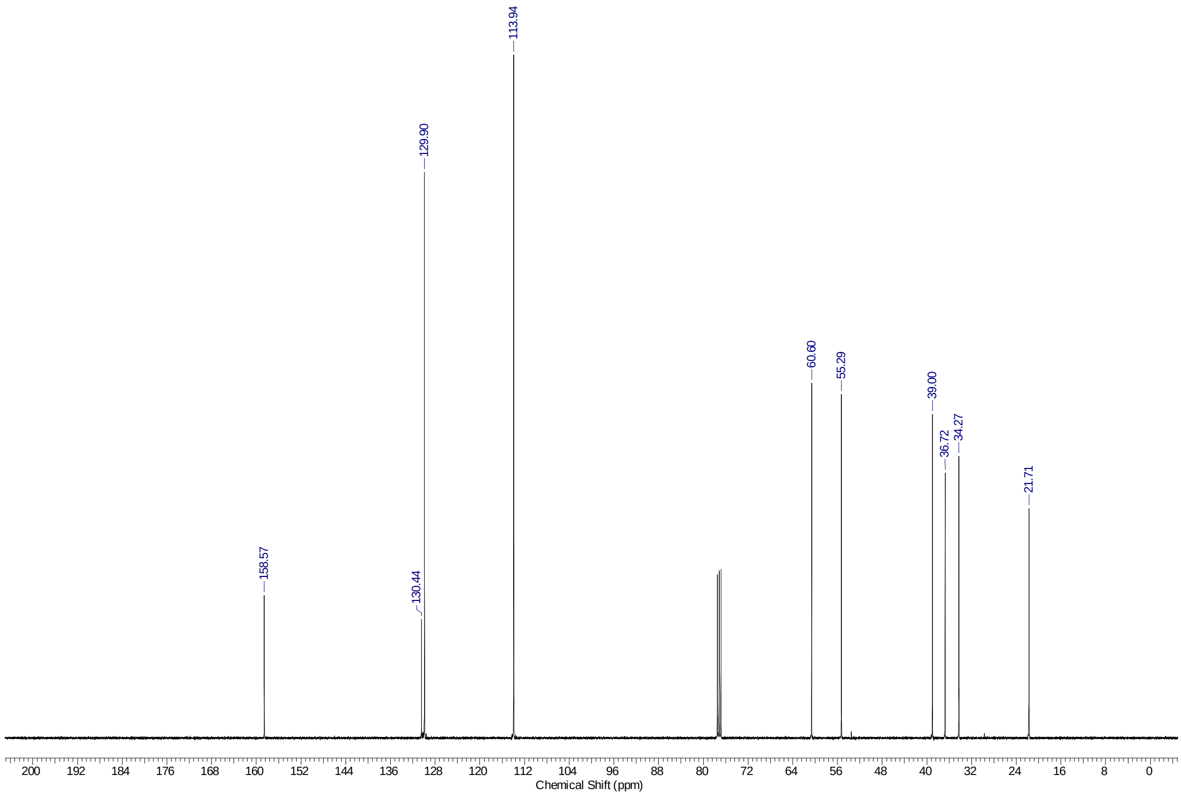

^{1}H NMR of **12** (400 MHz, $CDCl_3$)

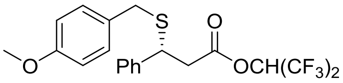

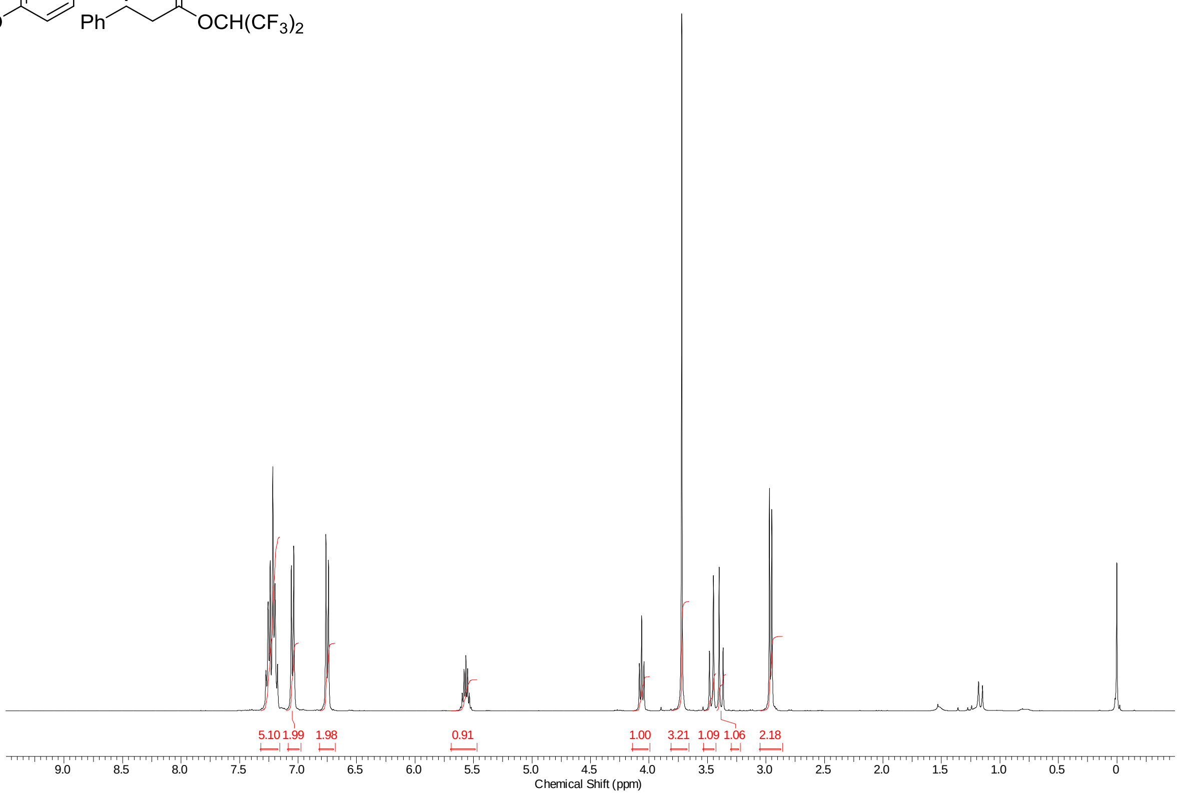

^{13}C NMR of **12** (100 MHz, $CDCl_3$)

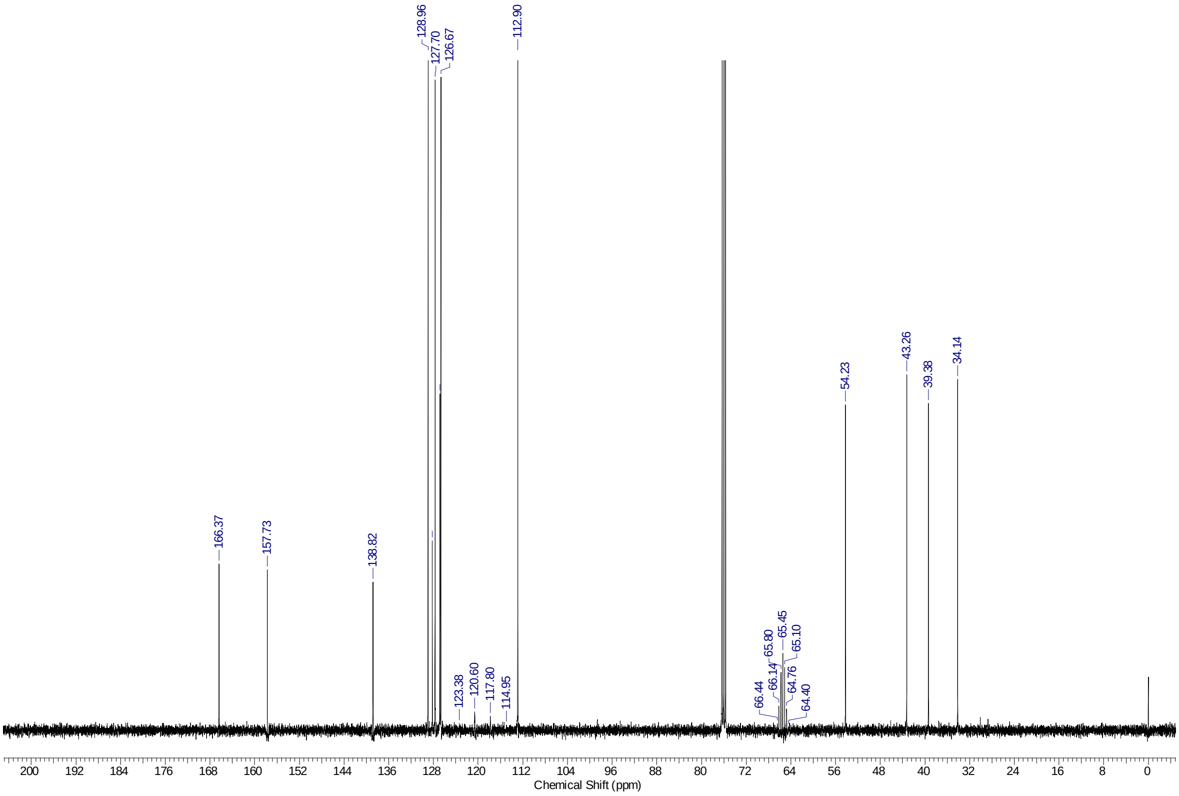

3.2 Copies of HPLC and GC spectra

HPLC and GC chromatograms of β-mercaptoesters 4 (section 1.5)

Methyl (*S*)-3-(propylthio)butanoate **4a**

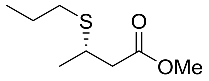

(Chiralpak IA, hexane/isopropanol = 99.5/0.5, 1 mL/min)

Racemic

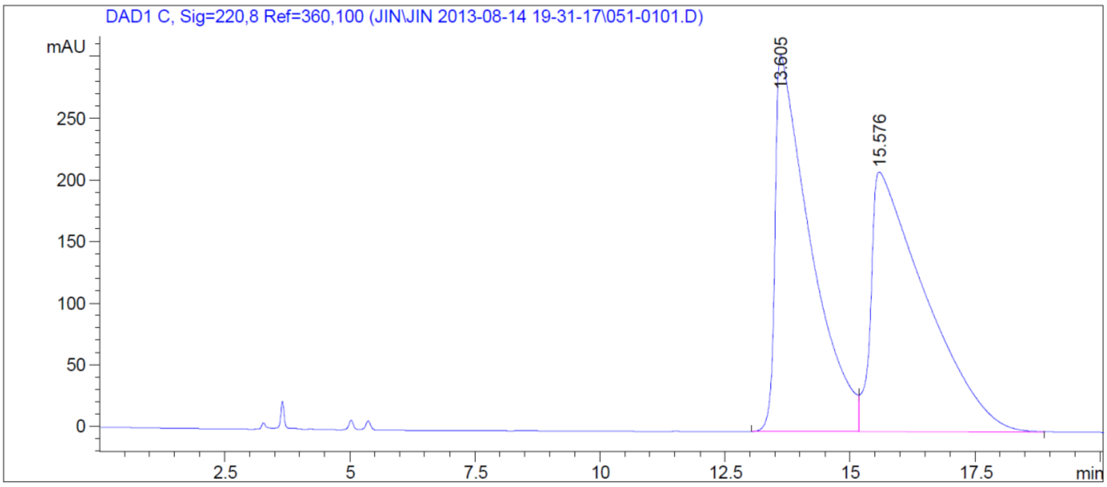

Peak #	RetTime [min]	Type	Width [min]	Area [mAU*s]	Height [mAU]	Area %
1	13.605	BV	0.6940	1.49835e4	305.58591	48.1785
2	15.576	VB	1.0642	1.61164e4	210.74866	51.8215

Enantiomerically enriched (81:19 er)

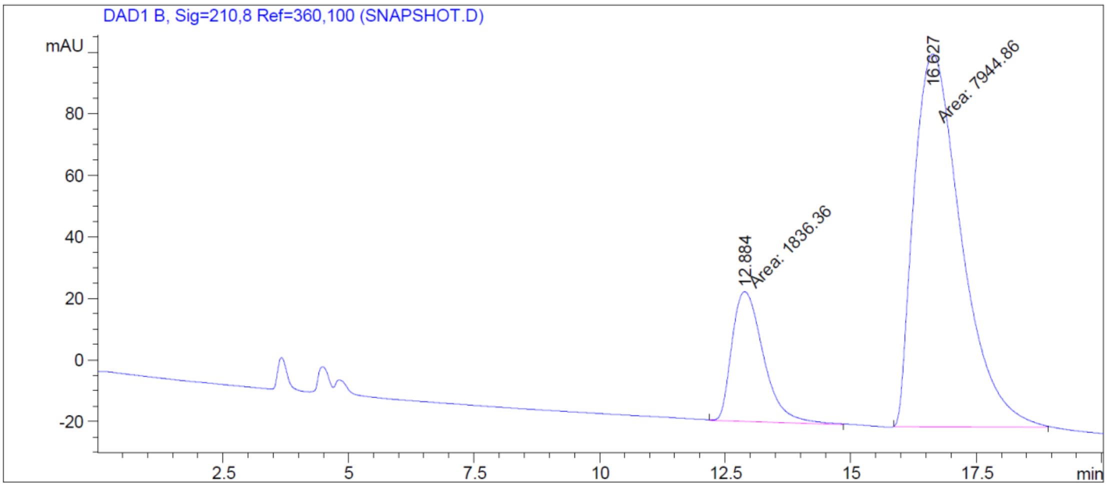

Peak #	RetTime [min]	Type	Width [min]	Area [mAU*s]	Height [mAU]	Area %
1	12.884	MM	0.7267	1836.36255	42.11743	18.7744
2	16.627	MM	1.0934	7944.86279	121.10498	81.2256

Ethyl (*S*)-3-(propylthio)butanoate **4b**

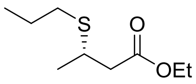

(Chiralpak IA, hexane/isopropanol = 100/0, 1 mL/min)

Racemic

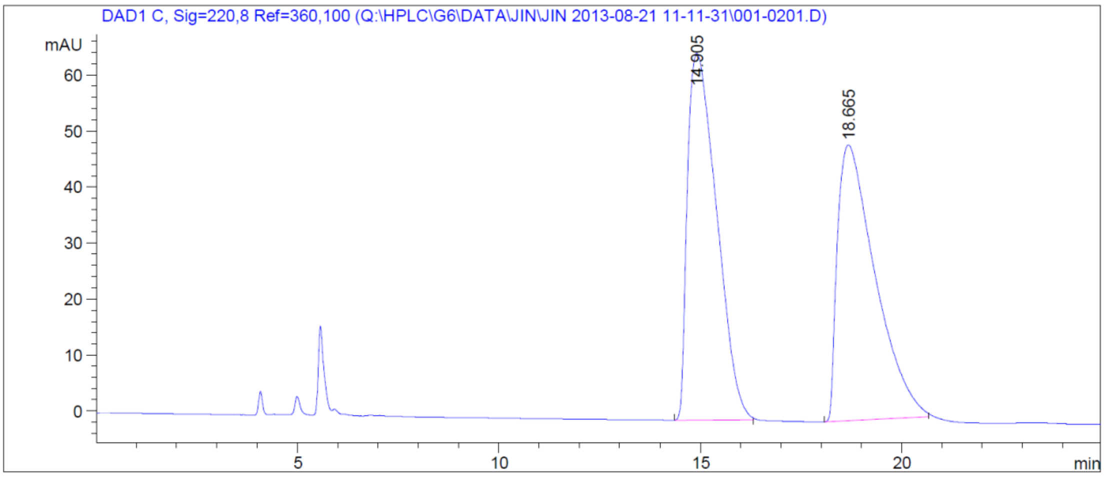

Signal 3: DAD1 C, Sig=220,8 Ref=360,100

Peak #	RetTime [min]	Type	Width [min]	Area [mAU*s]	Height [mAU]	Area %
1	14.905	BB	0.7777	3288.40430	65.50745	50.6757
2	18.665	BB	0.9565	3200.70508	49.23289	49.3243

Enantiomerically enriched (84:16 er)

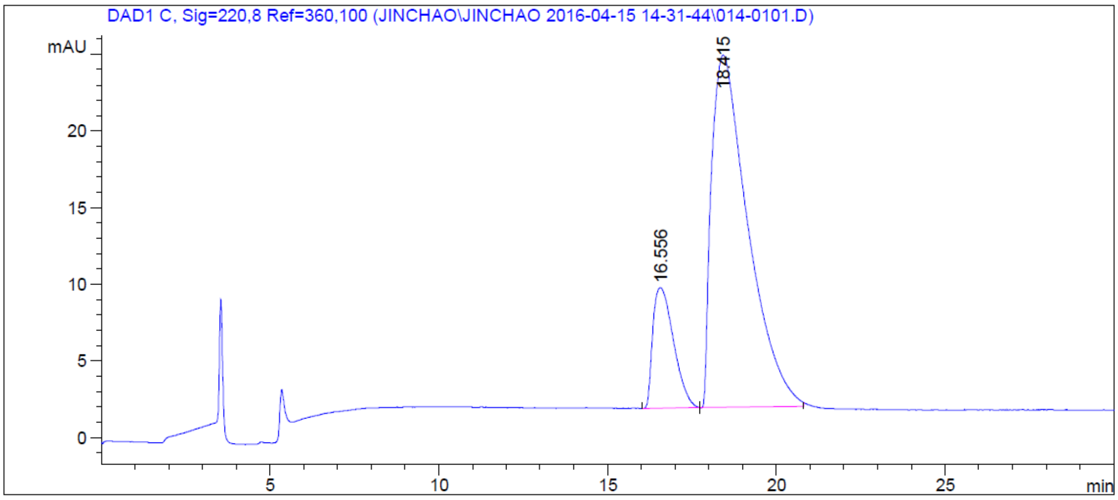

Peak #	RetTime [min]	Type	Width [min]	Area [mAU*s]	Height [mAU]	Area %
1	16.556	BB	0.6734	335.96121	7.84784	16.3077
2	18.415	BB	1.1274	1724.17236	22.97809	83.6923

Isopropyl (*S*)-3-(propylthio)butanoate **4c**

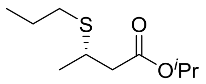

(Chiralpak AD-H, hexane/isopropanol = 99/1, 1 mL/min)

Racemic

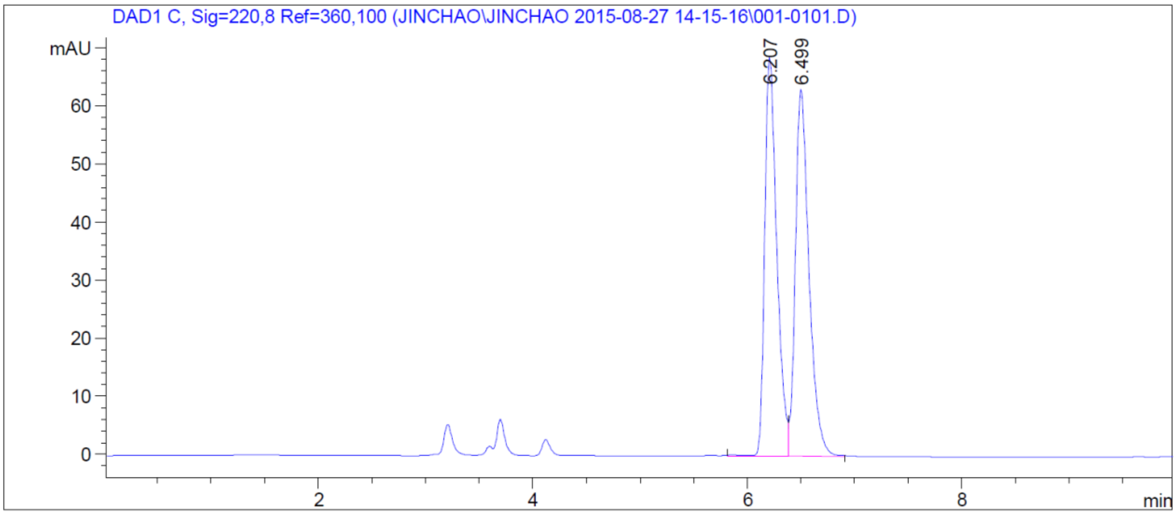

Peak #	RetTime [min]	Type	Width [min]	Area [mAU*s]	Height [mAU]	Area %
1	6.207	BV	0.1192	537.12067	68.69326	48.9867
2	6.499	VB	0.1334	559.34070	63.08997	51.0133

Enantiomerically enriched (85:15 er)

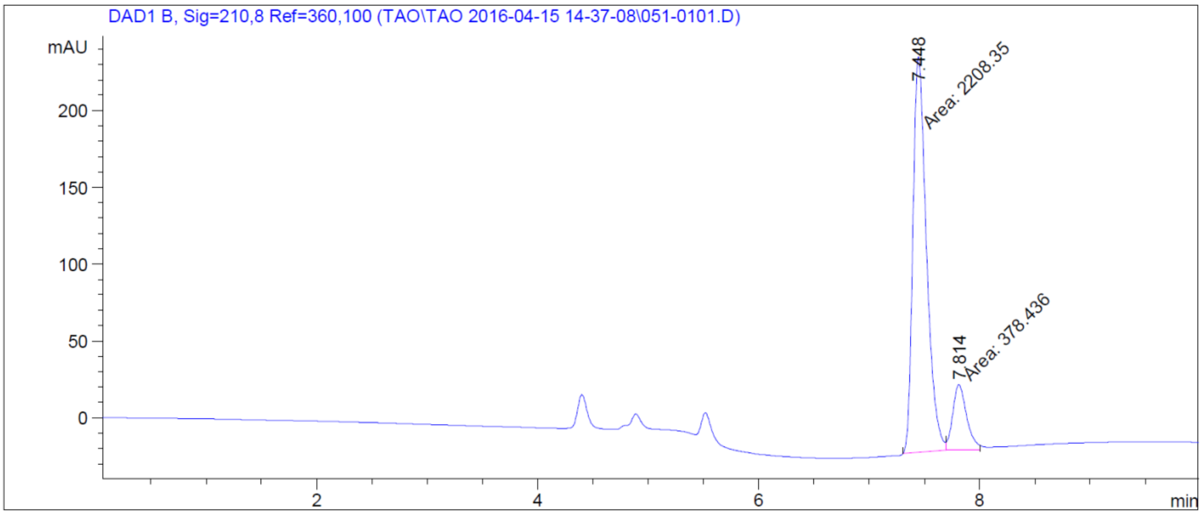

Peak #	RetTime [min]	Type	Width [min]	Area [mAU*s]	Height [mAU]	Area %
1	7.448	MM	0.1426	2208.35254	258.15961	85.3704
2	7.814	MM	0.1476	378.43585	42.73730	14.6296

Benzyl (*S*)-3-(propylthio)butanoate **4d**

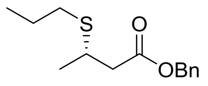

(Chiralpak AD-H, hexane/isopropanol = 99/1, 1 mL/min)

Racemic

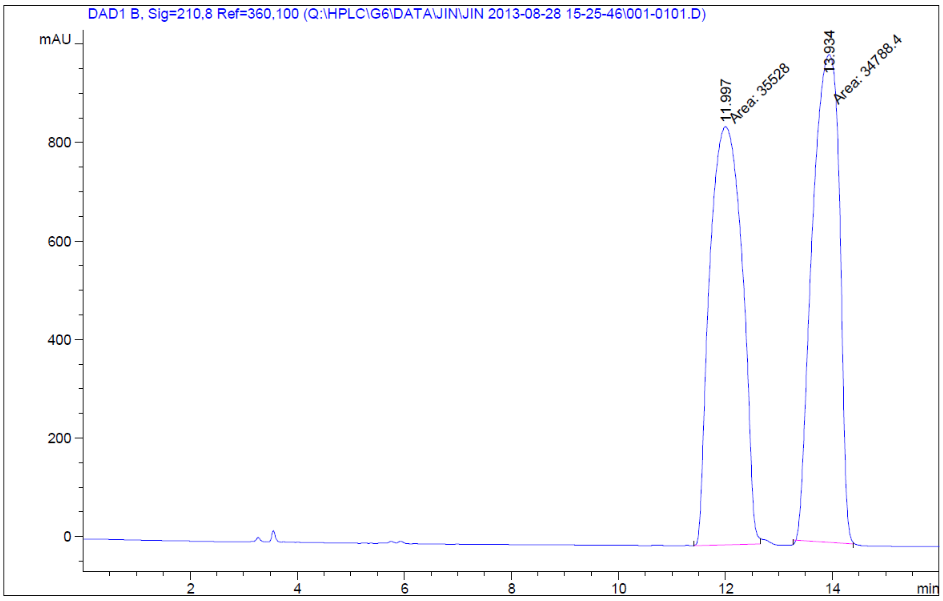

Peak #	RetTime [min]	Type	Width [min]	Area [mAU*s]	Height [mAU]	Area %
1	11.997	MM	0.6978	3.55280e4	848.51453	50.5259
2	13.934	MM	0.5857	3.47884e4	990.01239	49.4741

Enantiomerically enriched (81:19 er)

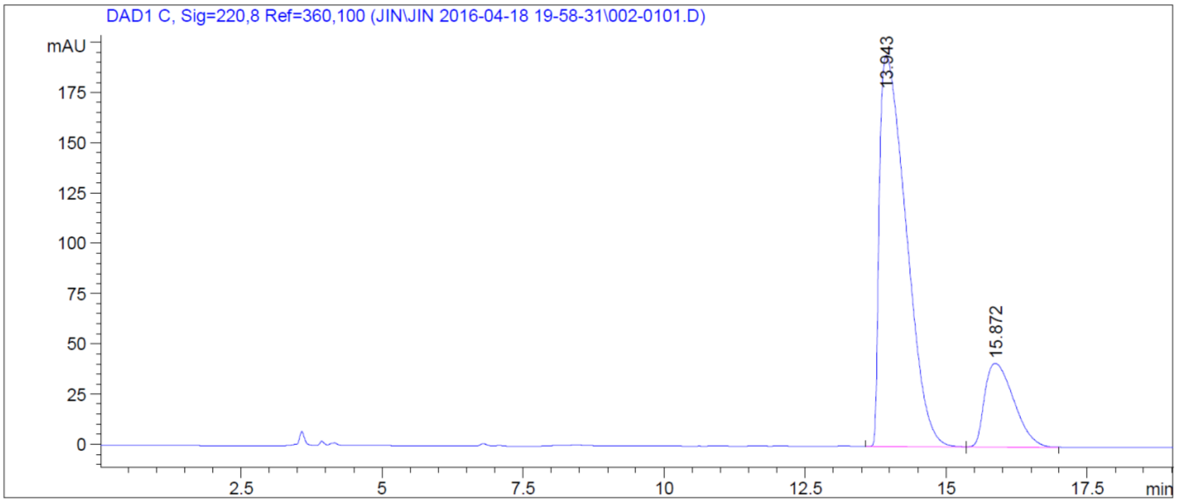

Peak #	RetTime [min]	Type	Width [min]	Area [mAU*s]	Height [mAU]	Area %
1	13.943	BB	0.5321	6380.69287	194.81694	81.0120
2	15.872	BB	0.5787	1495.53528	41.66831	18.9880

tert-Butyl (*S*)-3-(propylthio)butanoate **4e**

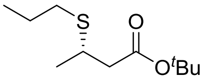

(Chiralpak IA, hexane/isopropanol = 100/0, 1 mL/min)

Racemic

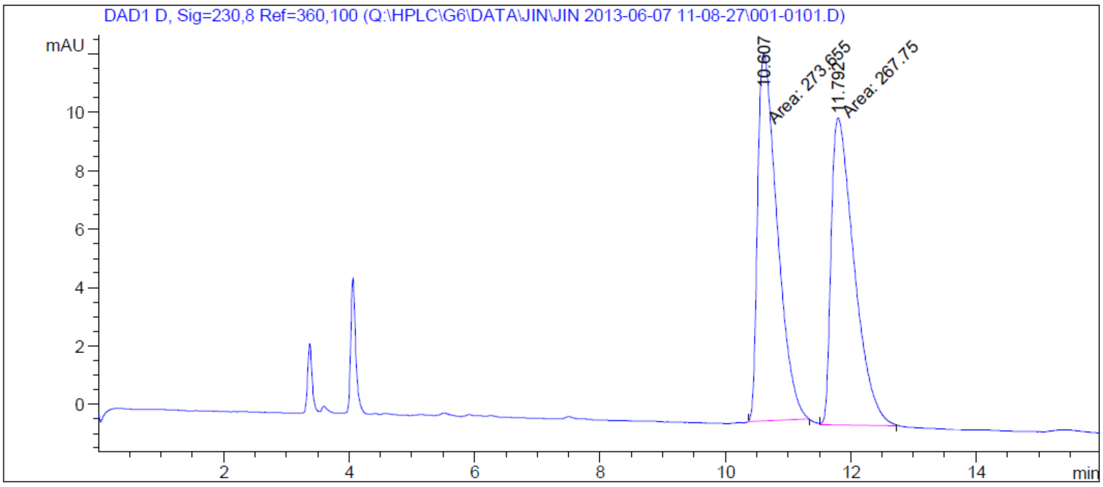

Signal 4: DAD1 D, Sig=230,8 Ref=360,100

Peak #	RetTime [min]	Type	Width [min]	Area [mAU*s]	Height [mAU]	Area %
1	10.607	MM	0.3626	273.65527	12.57780	50.5453
2	11.792	MM	0.4243	267.75049	10.51638	49.4547

Enantiomerically enriched (97:3 er)

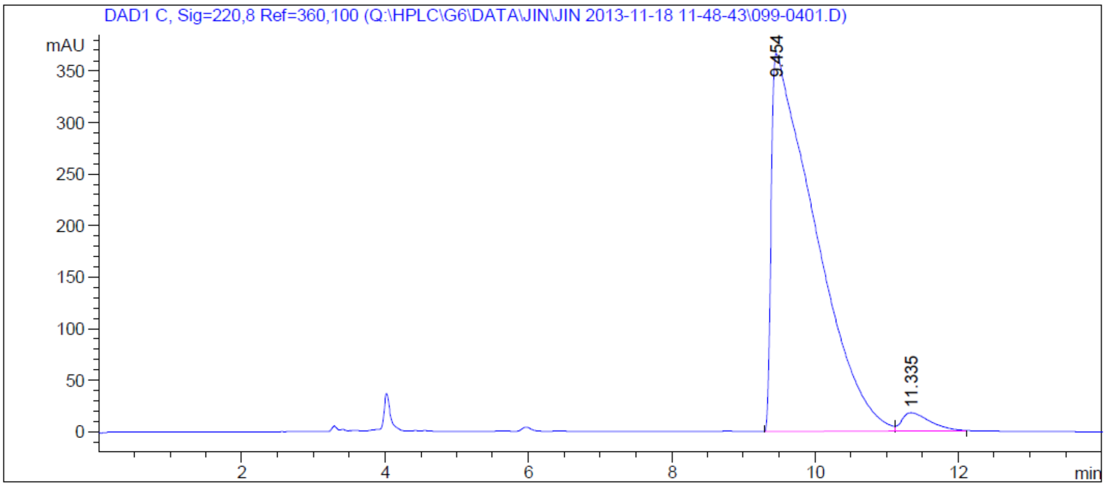

Signal 3: DAD1 C, Sig=220,8 Ref=360,100

Peak #	RetTime [min]	Type	Width [min]	Area [mAU*s]	Height [mAU]	Area %
1	9.454	BV	0.5481	1.55955e4	366.78867	96.9281
2	11.335	VB	0.4091	494.25604	17.84553	3.0719

tert-Butyl (*S*)-3-(pentylthio)butanoate **4f**

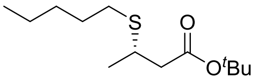

(Chiralpak IA, hexane/isopropanol = 100/0, 1 mL/min)

Racemic

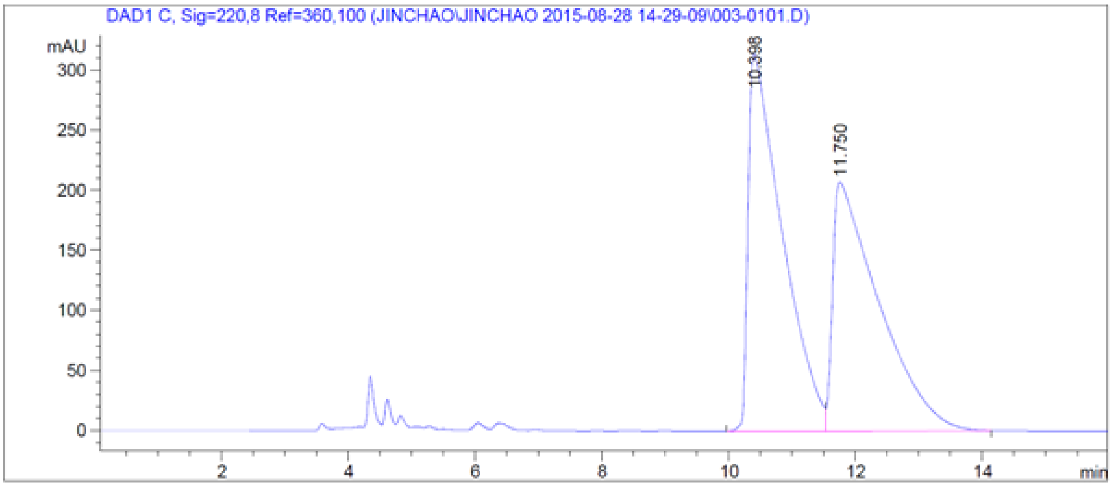

Signal 3: DAD1 C, Sig=220,8 Ref=360,100

Peak #	RetTime [min]	Type	Width [min]	Area [mAU*s]	Height [mAU]	Area %
1	10.398	BV	0.5348	1.17456e4	313.73355	52.7505
2	11.750	VB	0.7025	1.05207e4	207.14561	47.2495

Enantiomerically enriched (95:5 er)

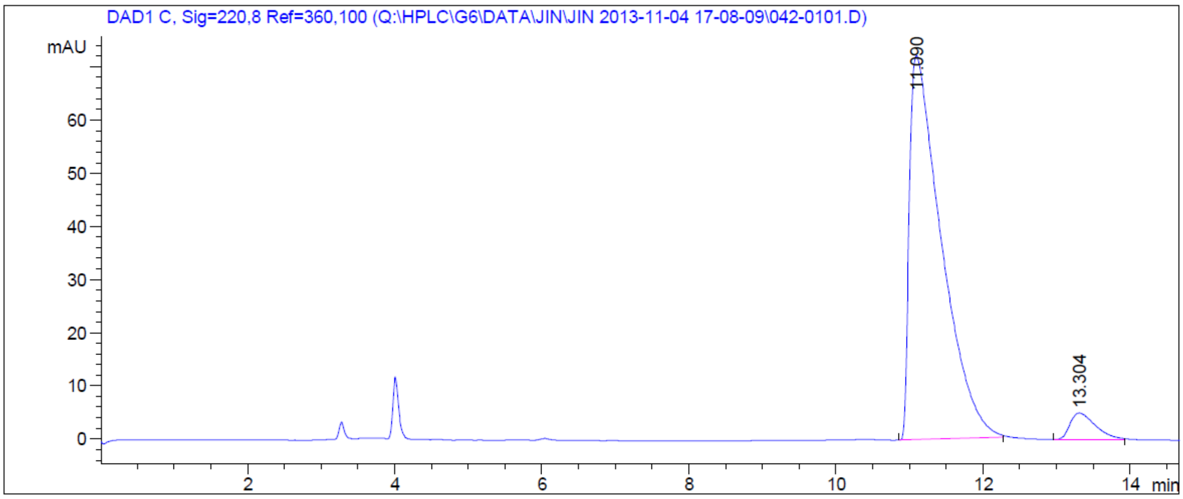

Signal 3: DAD1 C, Sig=220,8 Ref=360,100

Peak #	RetTime [min]	Type	Width [min]	Area [mAU*s]	Height [mAU]	Area %
1	11.090	BB	0.4384	2180.99365	72.20281	94.8412
2	13.304	BB	0.3585	118.63420	4.96983	5.1588

tert-Butyl (*S*)-3-(isopropylthio)butanoate **4g**

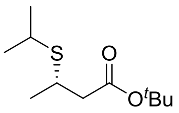

(Supelco β-dex^{TM} 325)

Racemic

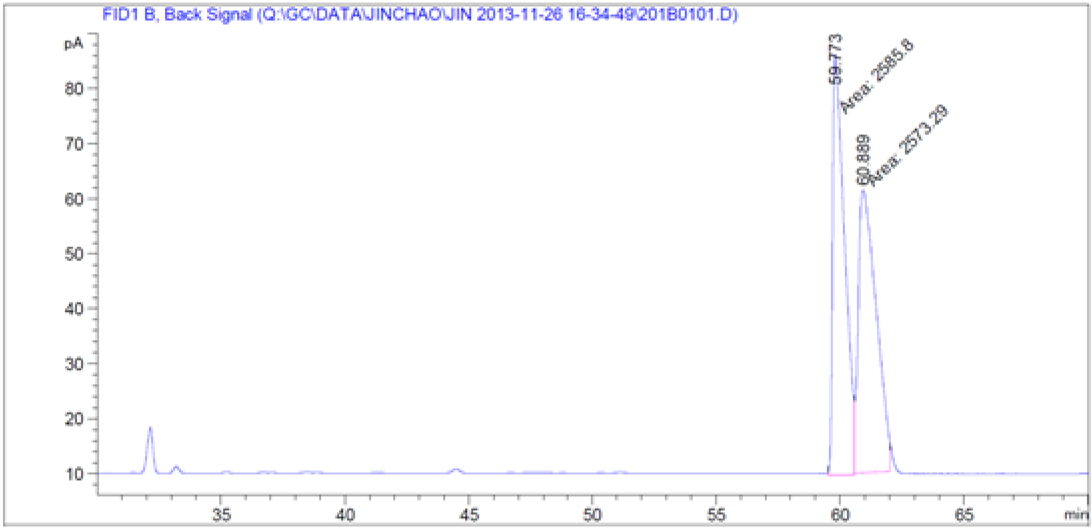

Signal 1: FID1 B, Back Signal

Peak #	RetTime [min]	Type	Width [min]	Area [pA*s]	Height [pA]	Area %
1	59.773	MM	0.5644	2585.79736	76.35378	50.12123
2	60.889	MM	0.8346	2573.28882	51.38985	49.87877

Enantiomerically enriched (96:4 er)

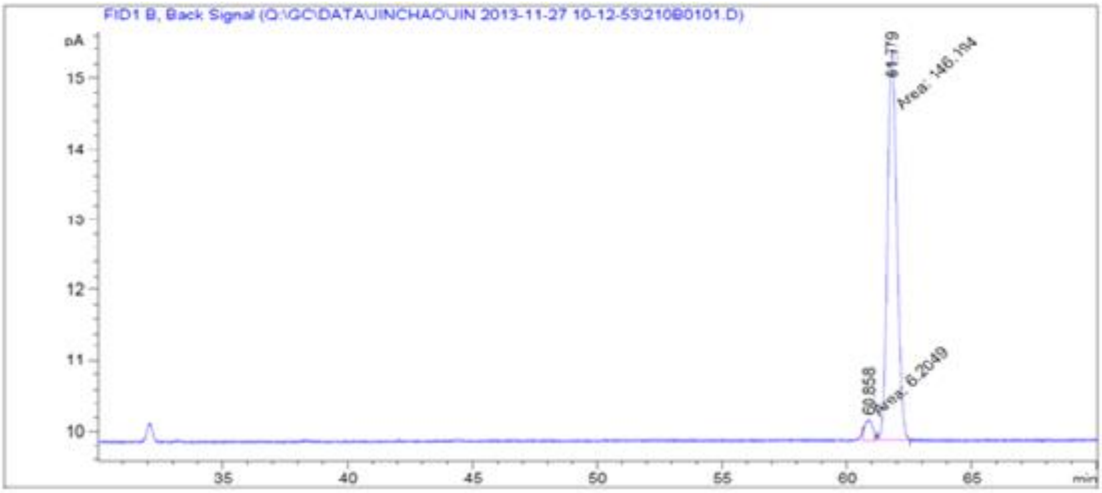

Signal 1: FID1 B, Back Signal

Peak #	RetTime [min]	Type	Width [min]	Area [pA*s]	Height [pA]	Area %
1	60.858	MM	0.4049	6.79030	2.79475e-1	4.43855
2	61.779	MM	0.4430	146.19440	5.50011	95.56145

tert-Butyl (*S*)-3-(isopentylthio)butanoate **4h**

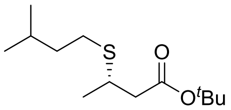

(Chiralpak IA, hexane/isopropanol = 100/0, 1 mL/min)

Racemic

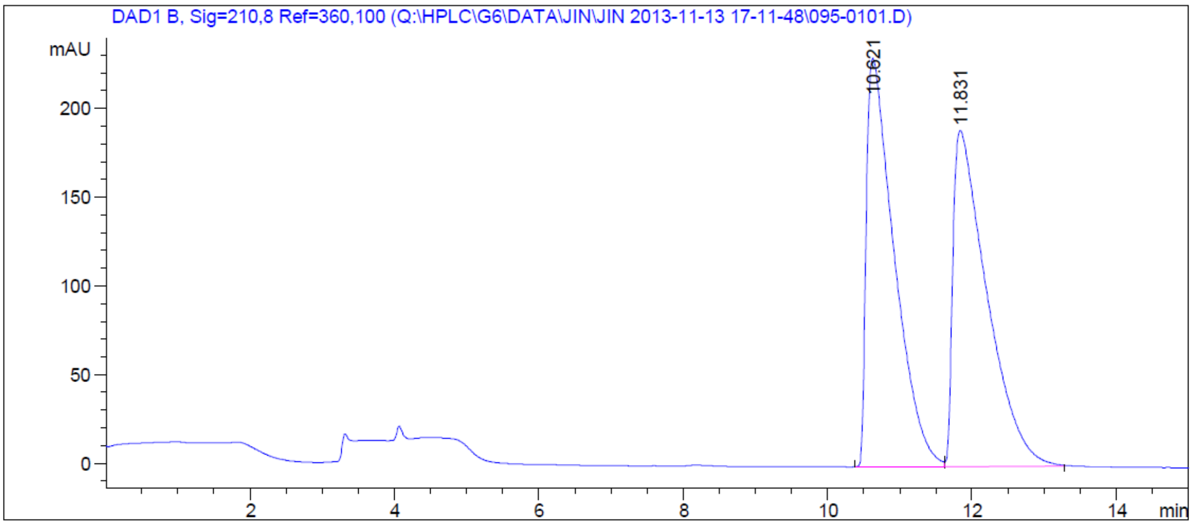

Signal 2: DAD1 B, Sig=210,8 Ref=360,100

Peak #	RetTime [min]	Type	Width [min]	Area [mAU*s]	Height [mAU]	Area %
1	10.621	BV	0.3913	6184.95654	230.33221	49.9945
2	11.831	VB	0.4728	6186.31348	189.54134	50.0055

Enantiomerically enriched (95:5 er)

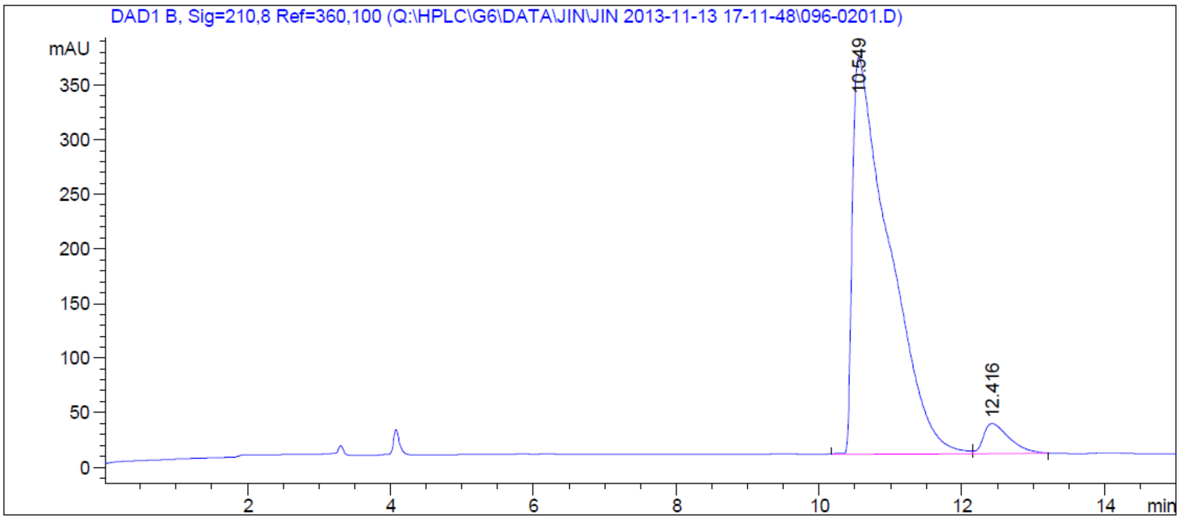

Signal 2: DAD1 B, Sig=210,8 Ref=360,100

Peak #	RetTime [min]	Type	Width [min]	Area [mAU*s]	Height [mAU]	Area %
1	10.549	BB	0.4910	1.29721e4	362.90451	94.9286
2	12.416	BB	0.3782	693.01221	27.47880	5.0714

tert-Butyl (*S*)-3-(decylthio)butanoate **4i**

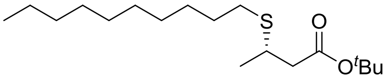

(Supelco β-dex™ 325)

Racemic

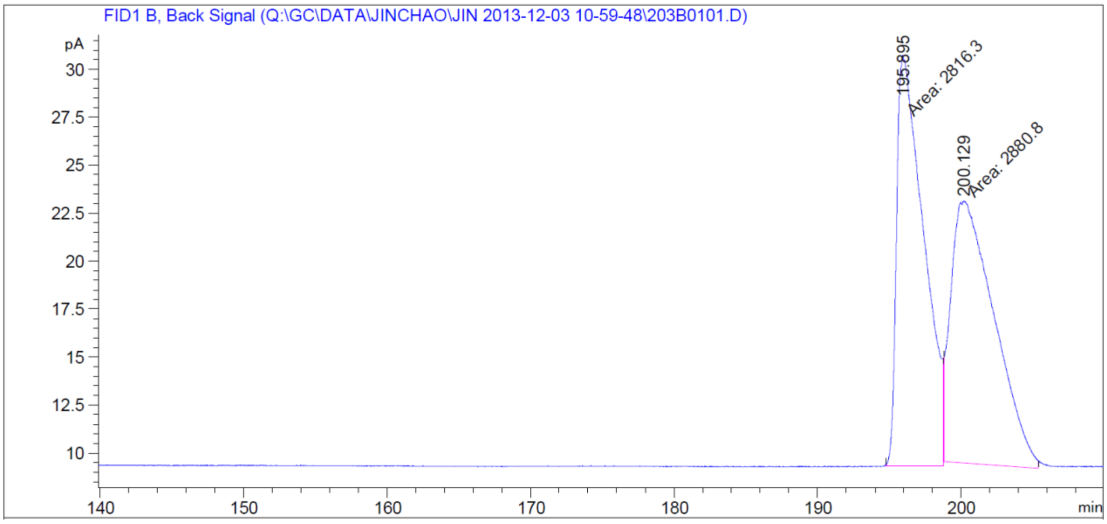

Peak #	RetTime [min]	Type	Width [min]	Area [pA*s]	Height [pA]	Area %
1	195.895	MM	2.1914	2816.29932	21.41913	49.43394
2	200.129	MM	3.5177	2880.79785	13.64887	50.56606

Enantiomerically enriched (95:5)

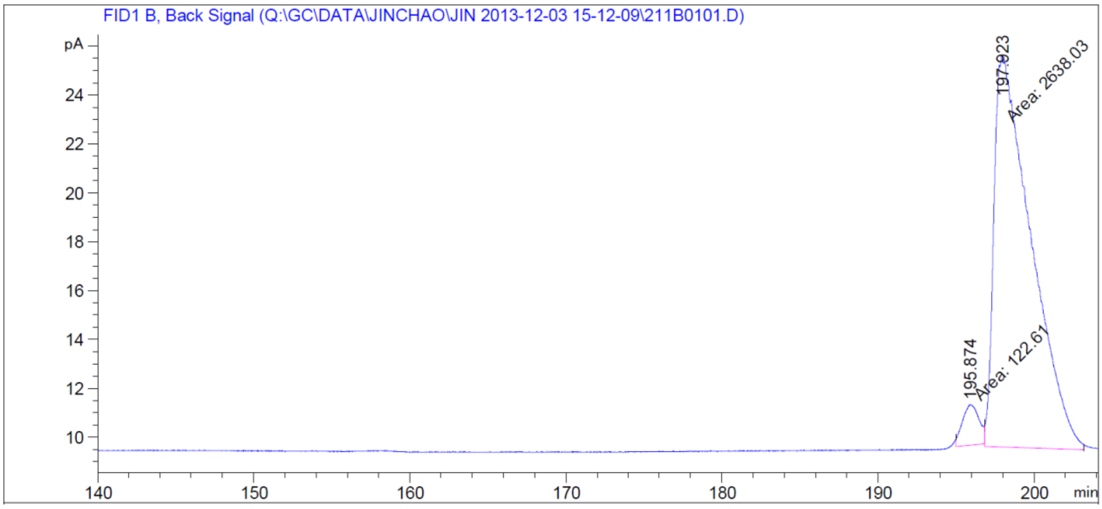

Signal 1: FID1 B, Back Signal

Peak #	RetTime [min]	Type	Width [min]	Area [pA*s]	Height [pA]	Area %
1	195.874	MM	1.2012	130.79546	1.81475	4.69734
2	197.923	MM	2.7481	2653.66333	16.09390	95.30266

tert-Butyl (*S*)-3-(cyclohexylthio)butanoate **4j**

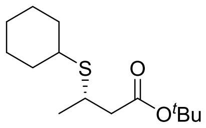

(Chiralpak IA, hexane/isopropanol = 100/0, 0.5 mL/min)

Racemic

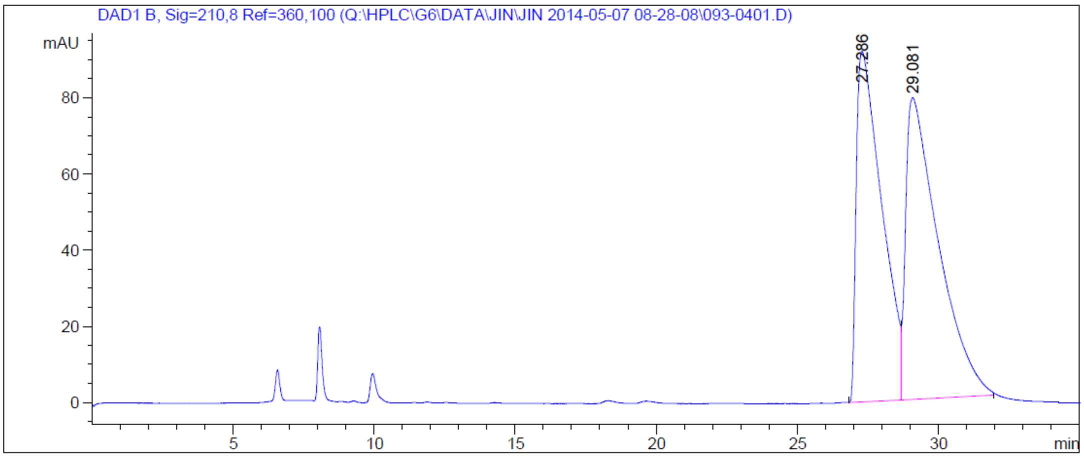

Signal 2: DAD1 B, Sig=210,8 Ref=360,100

Peak #	RetTime [min]	Type	Width [min]	Area [mAU*s]	Height [mAU]	Area %
1	27.286	BV	0.8855	5740.49951	92.05278	47.2667
2	29.081	VB	1.0967	6404.40234	79.09560	52.7333
Totals :				1.21449e4	171.14838	

Enantiomerically enriched (95:5 er)

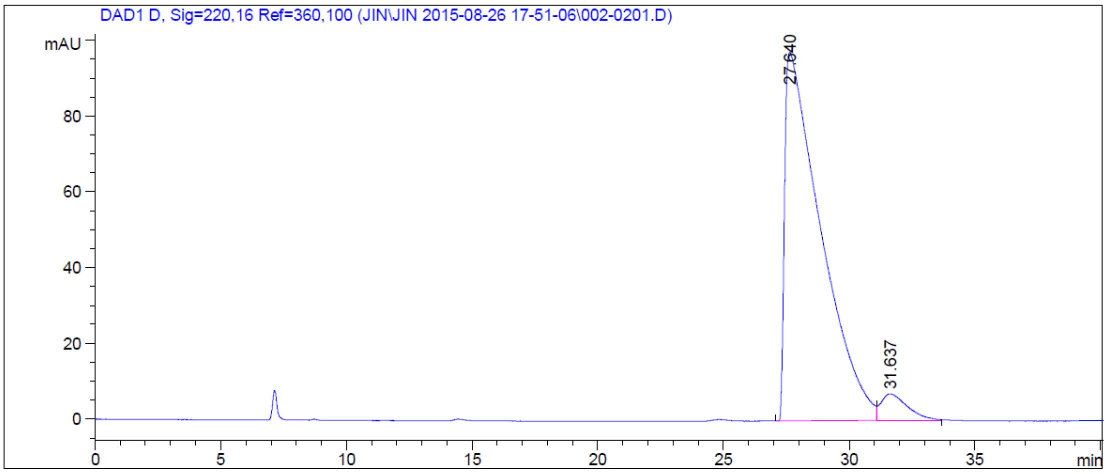

Signal 4: DAD1 D, Sig=220,16 Ref=360,100

Peak #	RetTime [min]	Type	Width [min]	Area [mAU*s]	Height [mAU]	Area %
1	27.640	BV	1.3216	9691.12891	97.32992	94.9290
2	31.637	VB	0.9968	517.68457	7.01612	5.0710

tert-Butyl (*S*)-3-(cyclopentylthio)butanoate **4k**

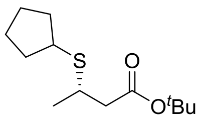

(Chiralpak IA, hexane/isopropanol = 100/0, 1.0 mL/min)

Racemic

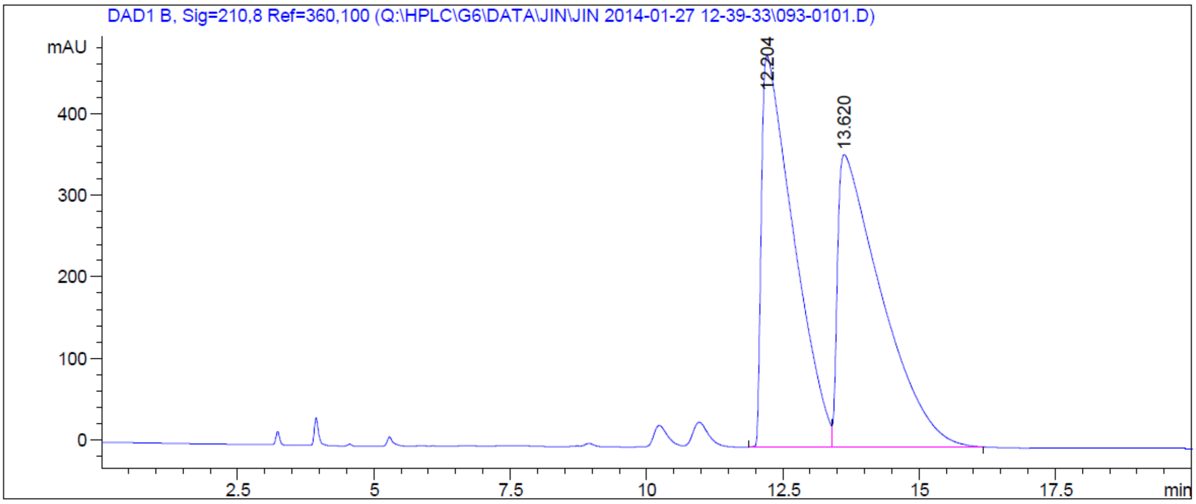

Signal 2: DAD1 B, Sig=210,8 Ref=360,100

Peak #	RetTime [min]	Type	Width [min]	Area [mAU*s]	Height [mAU]	Area %
1	12.204	BV	0.5959	1.96327e4	479.27637	49.3124
2	13.620	VB	0.7928	2.01802e4	358.46552	50.6876

Enantiomerically enriched (96:4 er)

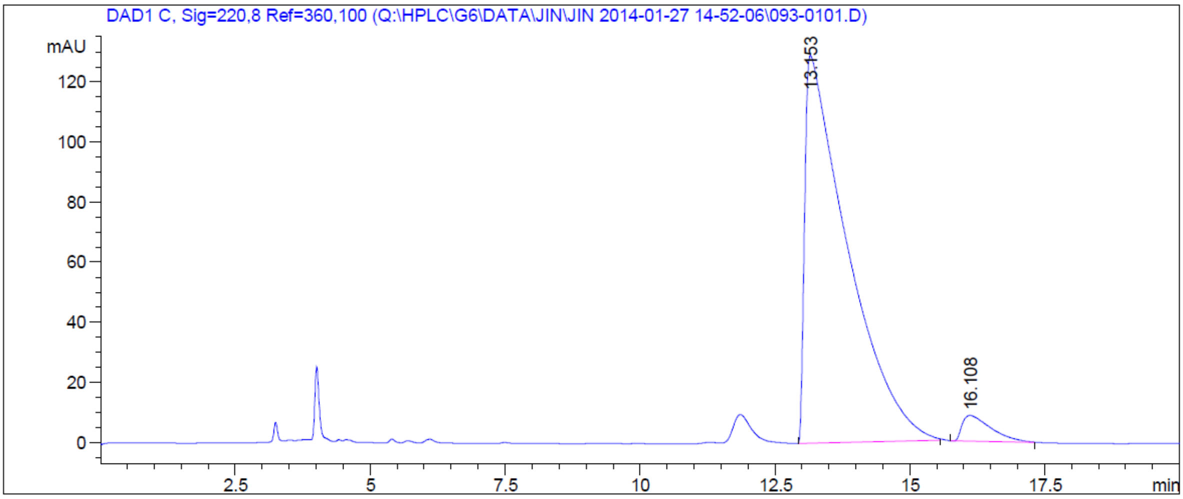

Signal 3: DAD1 C, Sig=220,8 Ref=360,100

Peak #	RetTime [min]	Type	Width [min]	Area [mAU*s]	Height [mAU]	Area %
1	13.153	BB	0.7463	7085.36377	129.10587	95.6882
2	16.108	BB	0.5462	319.27481	8.53992	4.3118

tert-Butyl (*S*)-3-(phenethylthio)butanoate **4l**

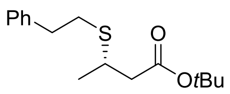

(Chiralpak IA, hexane/isopropanol = 100/0, 1.0 mL/min)

Racemic

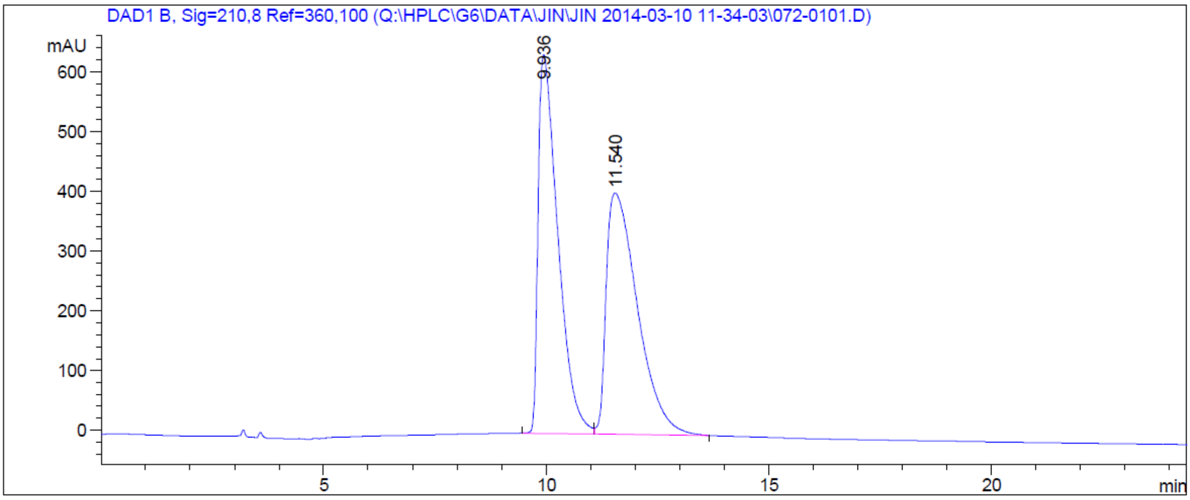

Signal 2: DAD1 B, Sig=210,8 Ref=360,100

Peak #	RetTime [min]	Type	Width [min]	Area [mAU*s]	Height [mAU]	Area %
1	9.936	BV	0.4662	1.95530e4	633.33875	49.7235
2	11.540	VB	0.7613	1.97704e4	403.75366	50.2765

Enantiomerically enriched (94:6 er)

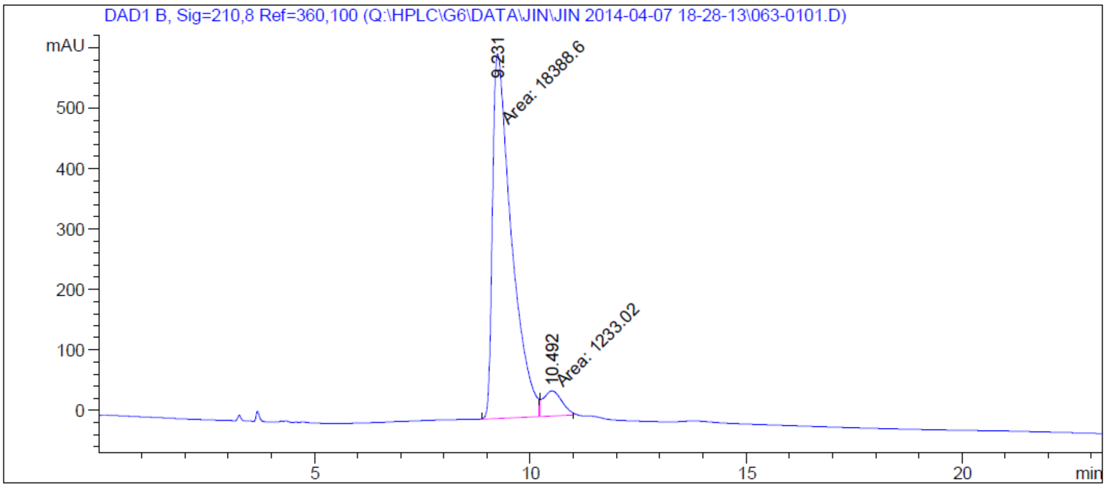

Signal 2: DAD1 B, Sig=210,8 Ref=360,100

Peak #	RetTime [min]	Type	Width [min]	Area [mAU*s]	Height [mAU]	Area %
1	9.231	MM	0.5083	1.83886e4	602.98889	93.7160
2	10.492	MM	0.4930	1233.02429	41.68432	6.2840

tert-Butyl (*S*)-3-((4-methoxybenzyl)thio)butanoate **4m**

(Chiralpak AS-H, hexane/isopropanol = 99/1, 1.0 mL/min)

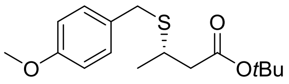

Racemic

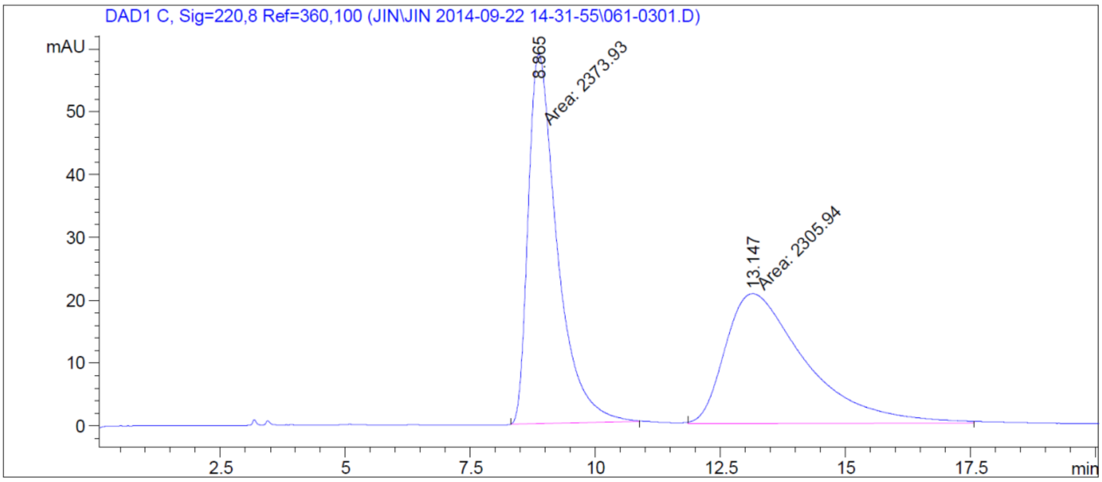

Signal 3: DAD1 C, Sig=220,8 Ref=360,100

Peak #	RetTime [min]	Type	Width [min]	Area [mAU*s]	Height [mAU]	Area %
1	8.865	MM	0.6728	2373.92944	58.80980	50.7265
2	13.147	MM	1.8599	2305.93555	20.66365	49.2735

Enantiomerically enriched (95:5 er)

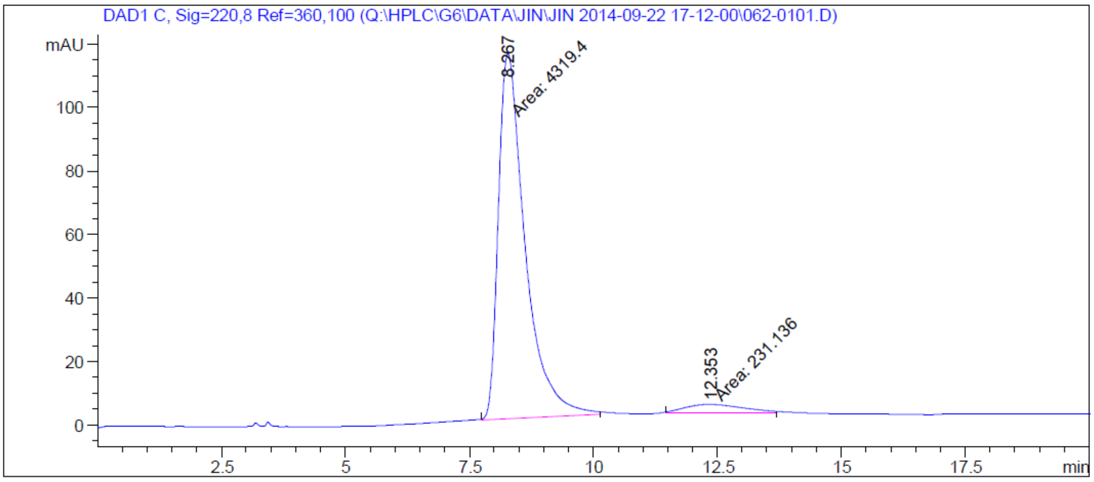

Signal 3: DAD1 C, Sig=220,8 Ref=360,100

Peak #	RetTime [min]	Type	Width [min]	Area [mAU*s]	Height [mAU]	Area %
1	8.267	MM	0.6258	4319.39697	115.02805	94.9207
2	12.353	MM	1.3925	231.13589	2.76634	5.0793

tert-Butyl (*R*)-3-((4-methoxybenzyl)thio)-3-phenylpropanoate **4n**

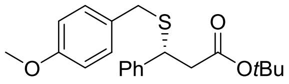

(Chiralpak AS-H, hexane/isopropanol = 100/0, 0.5 mL/min)

Racemic

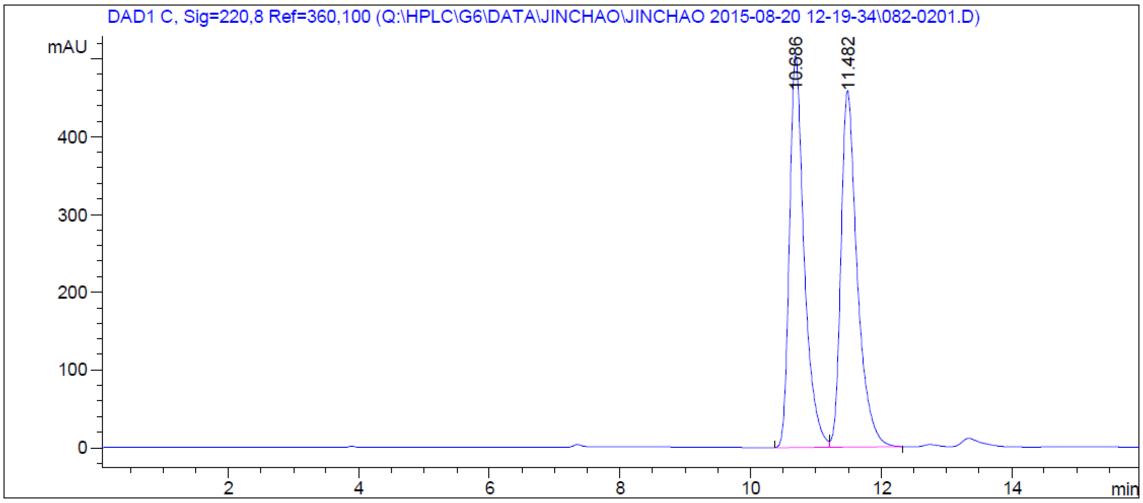

Signal 3: DAD1 C, Sig=220,8 Ref=360,100

Peak #	RetTime [min]	Type	Width [min]	Area [mAU*s]	Height [mAU]	Area %
1	10.686	BV	0.2346	7883.91064	503.62131	49.8419
2	11.482	VB	0.2579	7933.92822	458.48947	50.1581

Enantiomerically enriched (88:12 er)

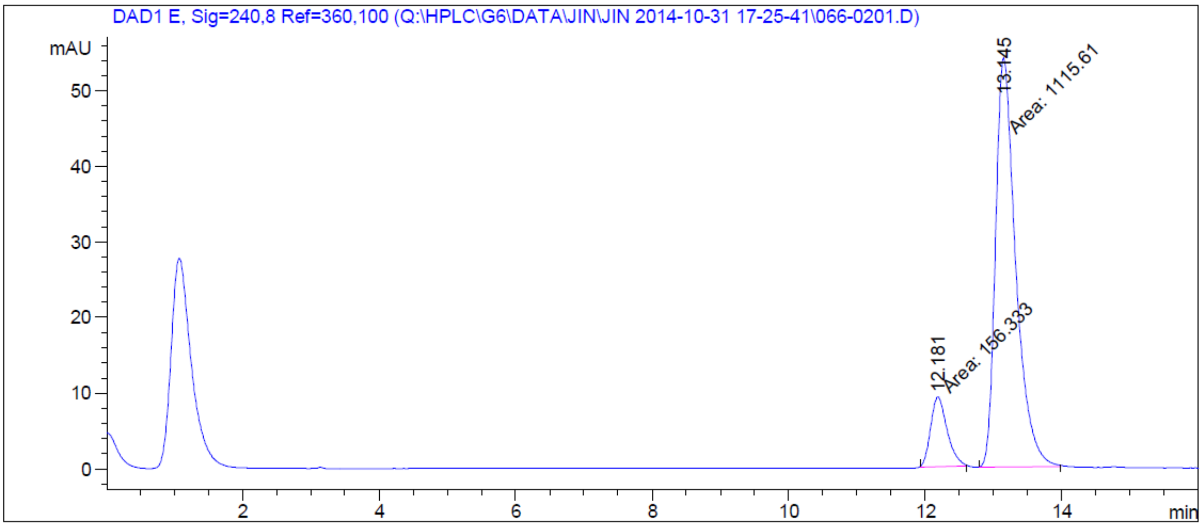

Signal 5: DAD1 E, Sig=240,8 Ref=360,100

Peak #	RetTime [min]	Type	Width [min]	Area [mAU*s]	Height [mAU]	Area %
1	12.181	MM	0.2820	156.33324	9.23939	12.2909
2	13.145	MM	0.3432	1115.60718	54.18087	87.7091

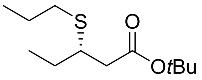

tert-Butyl (*S*)-3-(propylthio)pentanoate **4o**

(Chiralpak IA, hexane/isopropanol = 100/0, 1.0 mL/min)

Racemic

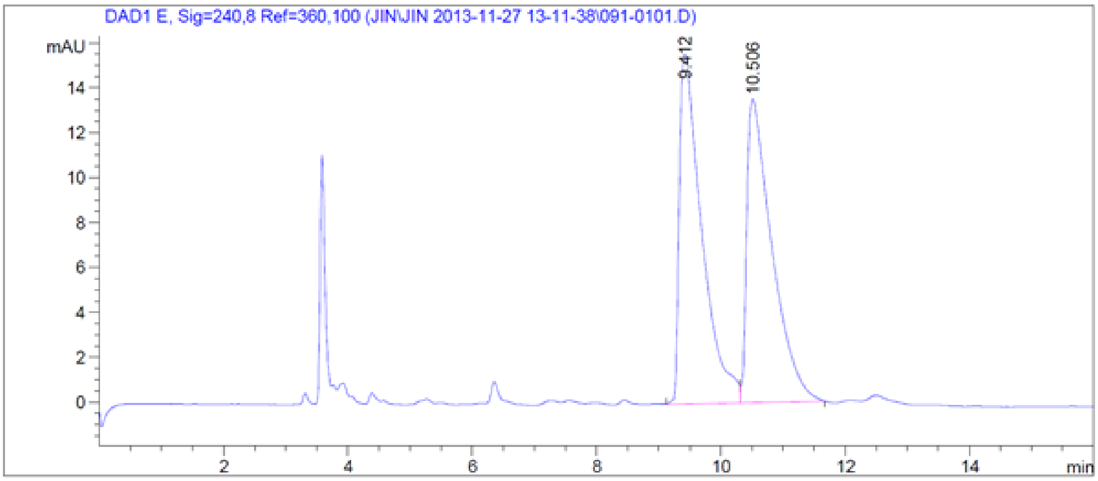

Peak #	RetTime [min]	Type	Width [min]	Area [mAU*s]	Height [mAU]	Area %
1	9.412	BV	0.3649	390.92239	15.56862	50.6792
2	10.506	VB	0.4005	380.44342	13.51196	49.3208

Enantiomerically enriched (93:7 er)

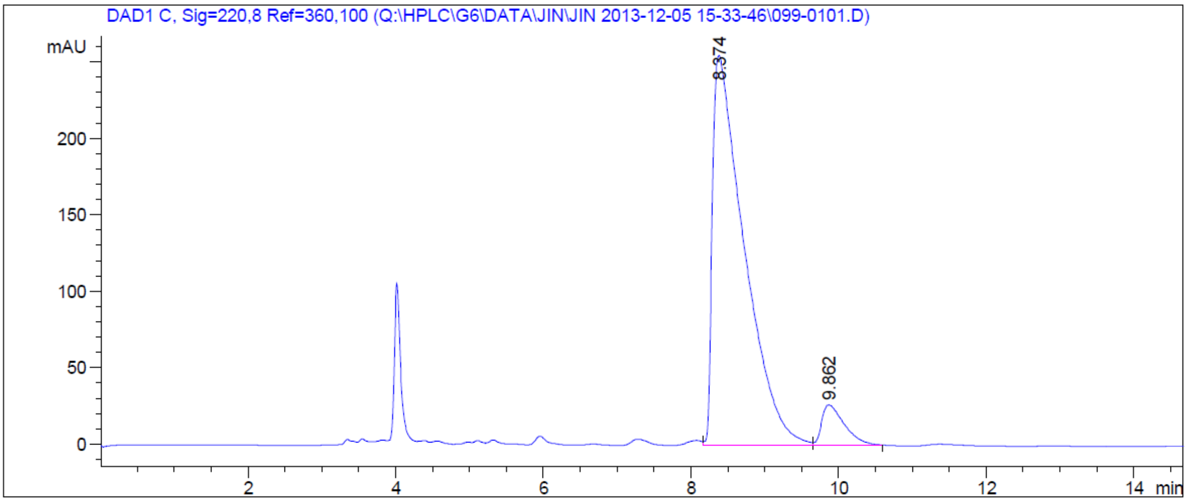

Signal 3: DAD1 C, Sig=220,8 Ref=360,100

Peak #	RetTime [min]	Type	Width [min]	Area [mAU*s]	Height [mAU]	Area %
1	8.374	VV	0.4239	7591.90527	254.59702	92.9248
2	9.862	VB	0.3276	578.03796	26.59521	7.0752

tert-Butyl (*S*)-3-(propylthio)decanoate **4p**

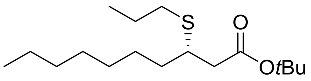

(Chiralpak IA, hexane/isopropanol = 100/0, 1.0 mL/min)

Racemic

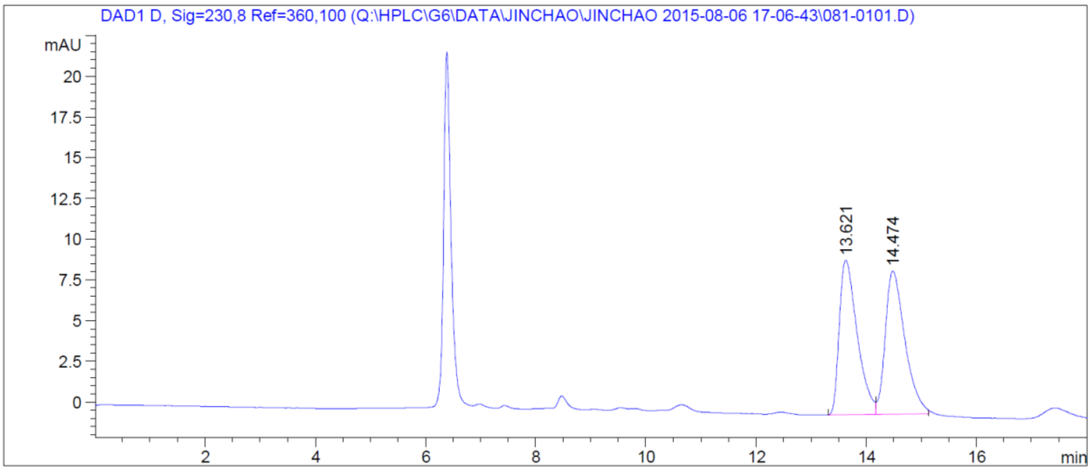

Signal 4: DAD1 D, Sig=230,8 Ref=360,100

Peak #	RetTime [min]	Type	Width [min]	Area [mAU*s]	Height [mAU]	Area %
1	13.621	BV	0.3411	213.85118	9.48187	49.5139
2	14.474	VB	0.3735	218.05049	8.78591	50.4861

Enantiomerically enriched (92:8 er)

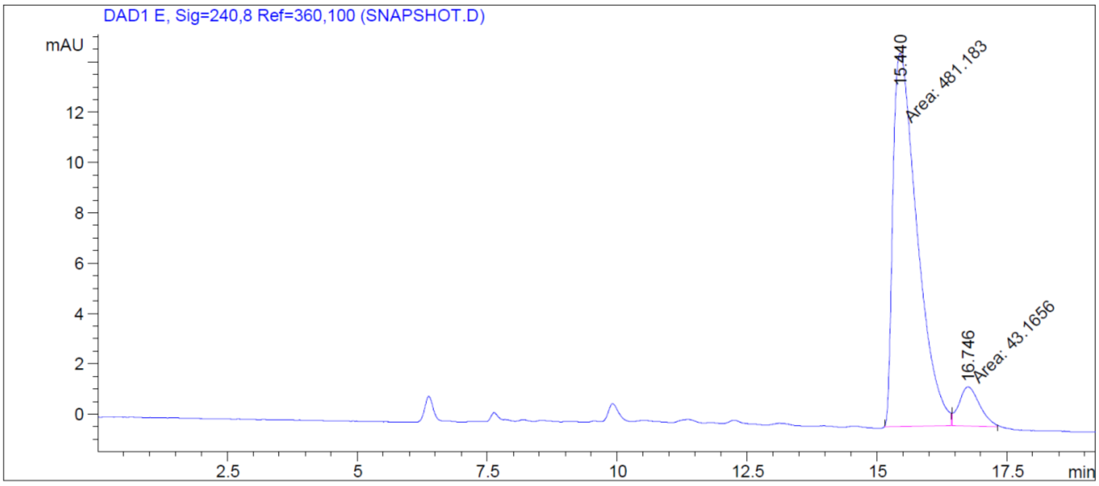

Signal 5: DAD1 E, Sig=240,8 Ref=360,100

Peak #	RetTime [min]	Type	Width [min]	Area [mAU*s]	Height [mAU]	Area %
1	15.440	MM	0.5403	481.18307	14.84291	91.7678
2	16.746	MM	0.4631	43.16562	1.55355	8.2322

tert-Butyl (*S*)-3-(propylthio)hept-6-enoate **4q**

(Chiralpak IA, hexane/isopropanol = 100/0, 1.0 mL/min)

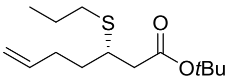

Racemic

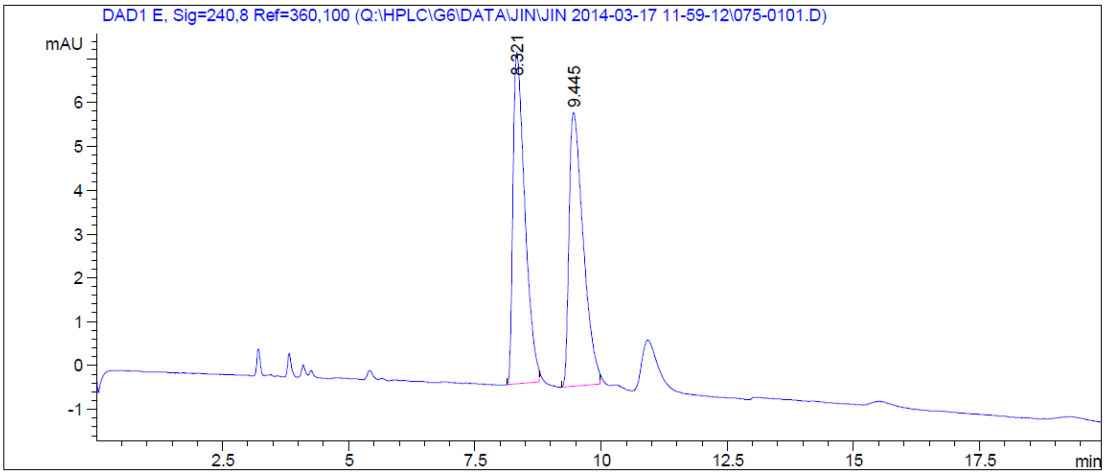

Signal 5: DAD1 E, Sig=240,8 Ref=360,100

Peak #	RetTime [min]	Type	Width [min]	Area [mAU*s]	Height [mAU]	Area %
1	8.321	BB	0.2490	123.80908	7.56425	50.3606
2	9.445	BB	0.2938	122.03627	6.24959	49.6394

Enantiomerically enriched (94:6 er)

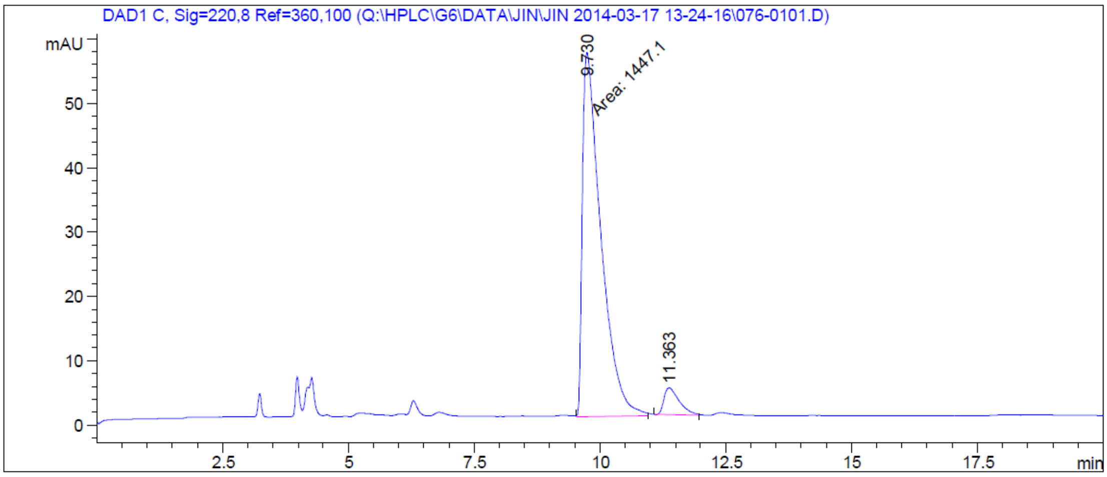

Peak #	RetTime [min]	Type	Width [min]	Area [mAU*s]	Height [mAU]	Area %
1	9.730	MM	0.4237	1438.33142	56.57480	94.2840
2	11.363	BB	0.3147	87.19946	4.15688	5.7160

tert-Butyl (*S*)-5-phenyl-3-(propylthio)pentanoate **4r**

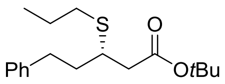

(Chiralpak IA, hexane/isopropanol = 100/0, 1.0 mL/min)

Racemic

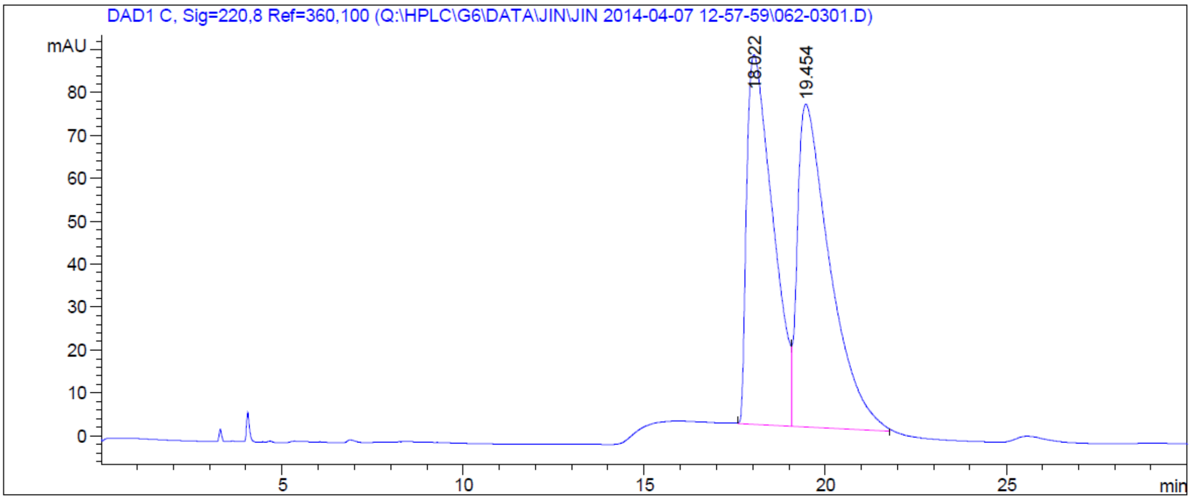

Signal 3: DAD1 C, Sig=220,8 Ref=360,100

Peak #	RetTime [min]	Type	Width [min]	Area [mAU*s]	Height [mAU]	Area %
1	18.022	BV	0.7339	4291.35791	86.03976	47.5955
2	19.454	VB	0.9075	4724.94727	75.15421	52.4045

Enantiomerically enriched (94:6 er)

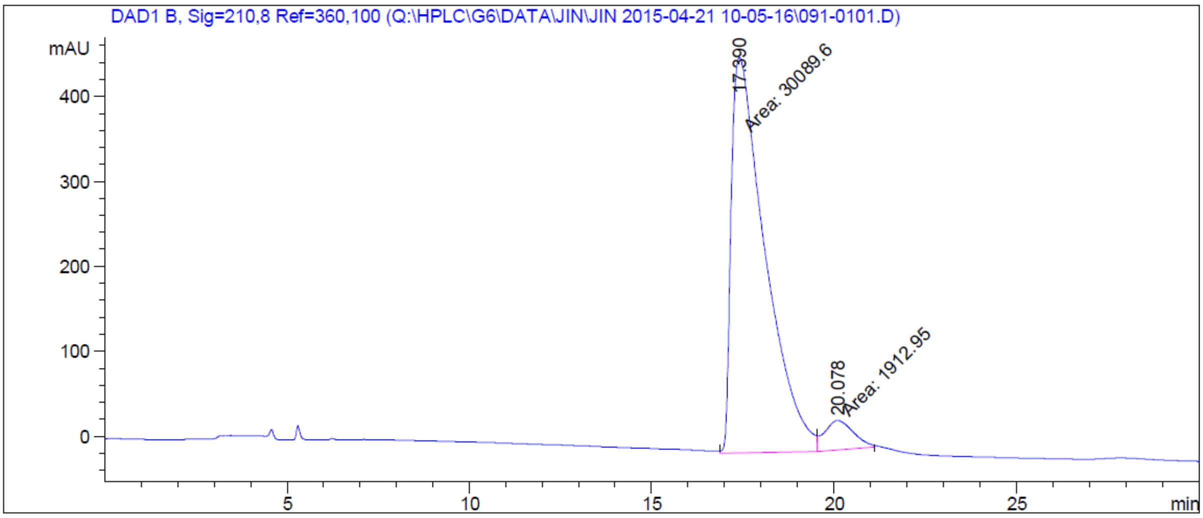

Signal 2: DAD1 B, Sig=210,8 Ref=360,100

Peak #	RetTime [min]	Type	Width [min]	Area [mAU*s]	Height [mAU]	Area %
1	17.390	MM	1.0769	3.00896e4	465.69724	94.0225
2	20.078	MM	0.9175	1912.95203	34.74918	5.9775

tert-Butyl (*S*)-5-((*tert*-butoxycarbonyl)amino)-3-(propylthio)pentanoate **4s**

(Chiralpak AD-H, hexane/isopropanol = 99/1, 1.0 mL/min)

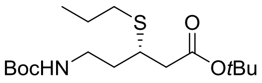

Racemic

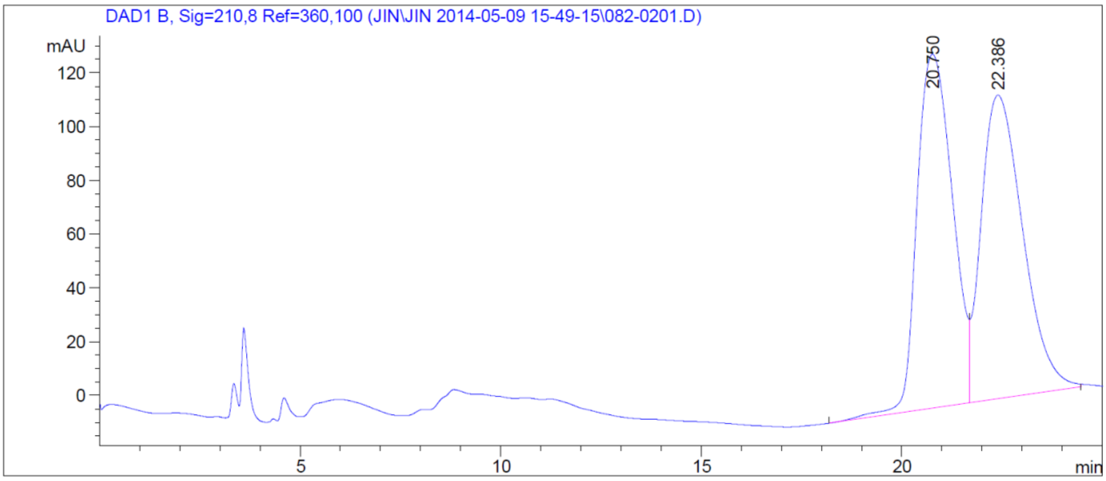

Peak #	RetTime [min]	Type	Width [min]	Area [mAU*s]	Height [mAU]	Area %
1	20.750	BV	0.9825	8089.42188	131.72157	49.8098
2	22.386	VB	1.1300	8151.19873	112.94682	50.1902

Enantiomerically enriched (94:6 er)

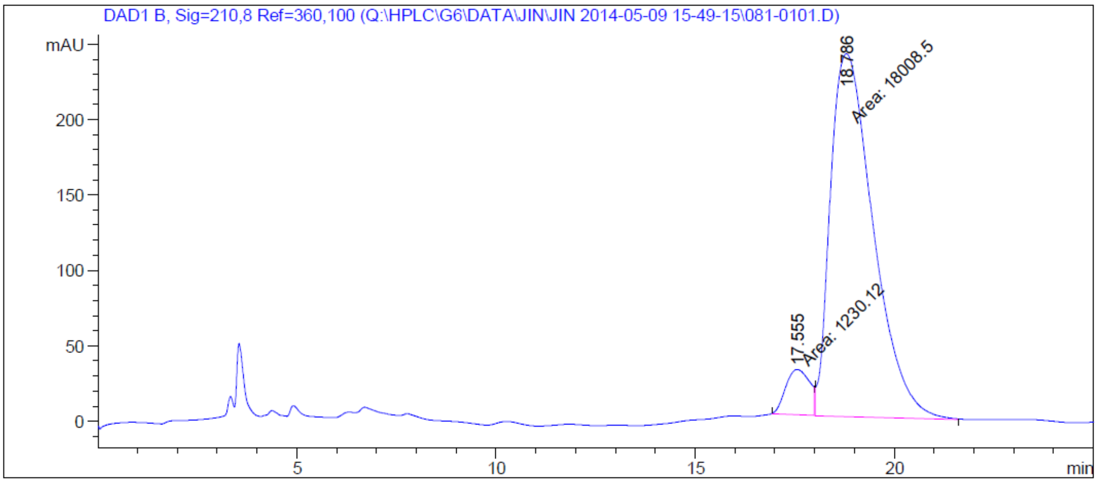

Signal 2: DAD1 B, Sig=210,8 Ref=360,100

Peak #	RetTime [min]	Type	Width [min]	Area [mAU*s]	Height [mAU]	Area %
1	17.555	MM	0.6863	1230.11829	29.87365	6.3940
2	18.786	MM	1.2488	1.80085e4	240.35182	93.6060

tert-Butyl (*S*)-5-((*tert*-butyldimethylsilyl)oxy)-3-(propylthio)pentanoate **4t**

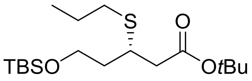

(Chiralpak IA, hexane/isopropanol = 100/0, 1.0 mL/min)
Racemic

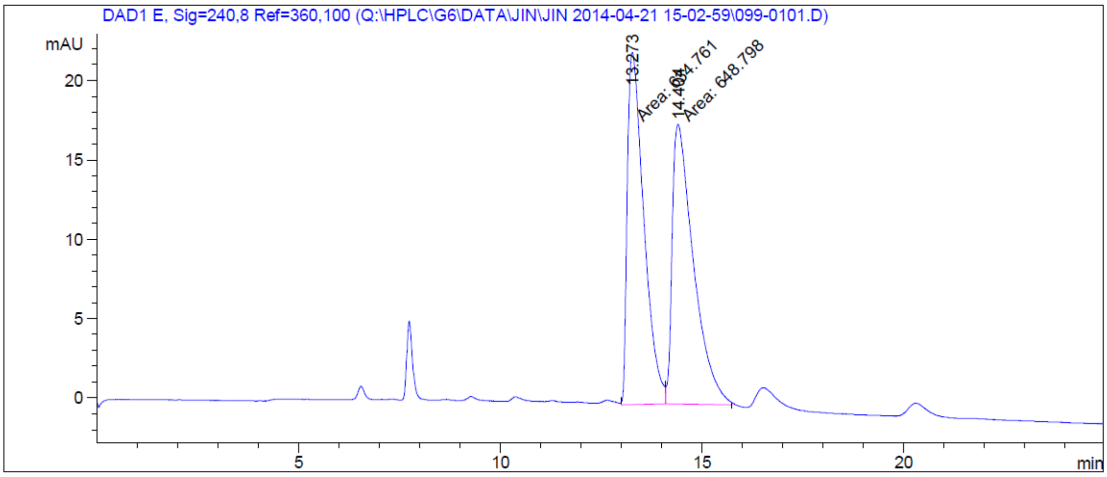

Signal 4: DAD1 D, Sig=230,8 Ref=360,100

Peak #	RetTime [min]	Type	Width [min]	Area [mAU*s]	Height [mAU]	Area %
1	13.273	BB	0.4343	1185.56799	41.62608	49.7337
2	14.405	BB	0.5257	1198.26245	32.99532	50.2663

Enantiomerically enriched (94:6 er)

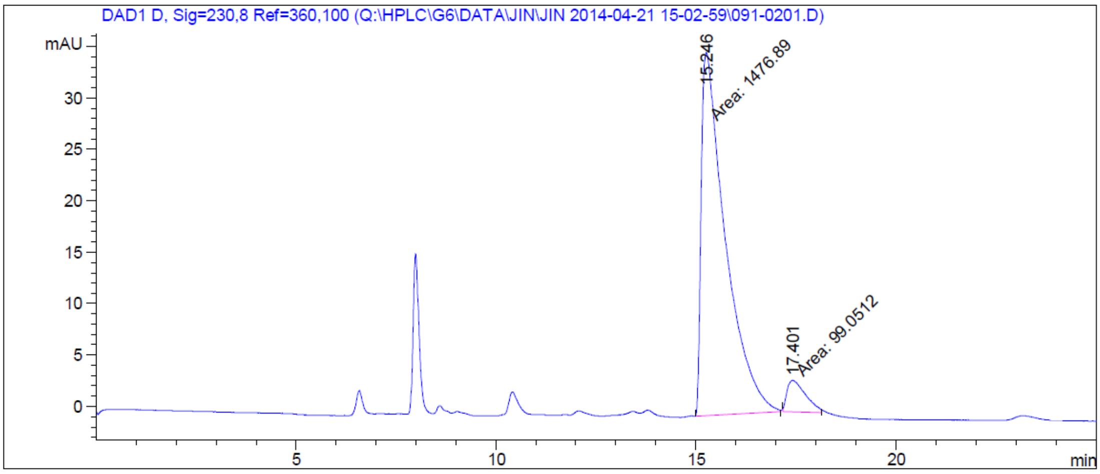

Signal 4: DAD1 D, Sig=230,8 Ref=360,100

Peak #	RetTime [min]	Type	Width [min]	Area [mAU*s]	Height [mAU]	Area %
1	15.246	MM	0.6967	1476.89294	35.33311	93.7148
2	17.401	MM	0.5397	99.05119	3.05866	6.2852

HPLC chromatogram of 4e for Section 1.6

tert-Butyl (*S*)-3-(propylthio)butanoate **4e**

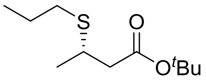

(Chiralpak IA, hexane/isopropanol = 100/0, 1 mL/min)

Enatiomerically enriched (94:6 er)

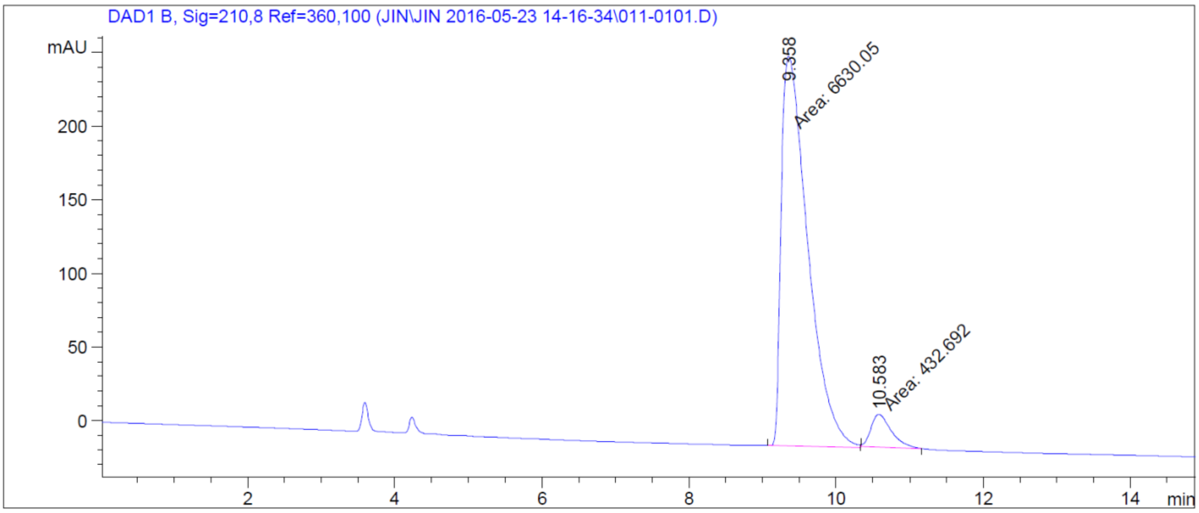

Signal 2: DAD1 B, Sig=210,8 Ref=360,100

Peak #	RetTime [min]	Type	Width [min]	Area [mAU*s]	Height [mAU]	Area %
1	9.358	MM	0.4177	6630.05322	264.54739	93.8736
2	10.583	MM	0.3235	432.69229	22.28986	6.1264

HPLC chromatograms for Section 1.7

tert-Butyl (*S*)-3-(propylsulfonyl)butanoate **5a**

(Chiralpak IA, hexane/isopropanol = 95/5, 1.0 mL/min)

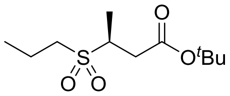

Racemic

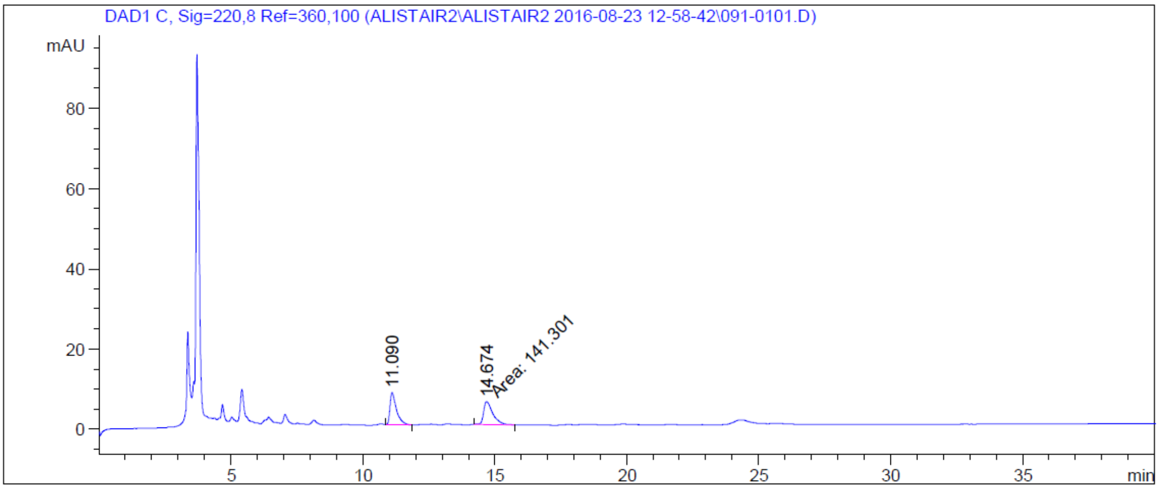

Signal 3: DAD1 C, Sig=220,8 Ref=360,100

Peak #	RetTime [min]	Type	Width [min]	Area [mAU*s]	Height [mAU]	Area %
1	11.090	VB	0.2591	141.76266	8.06321	50.0816
2	14.674	MM	0.4085	141.30054	5.76556	49.9184
Totals :				283.06320	13.82877	

Enantiomerically enriched (94:6 er)

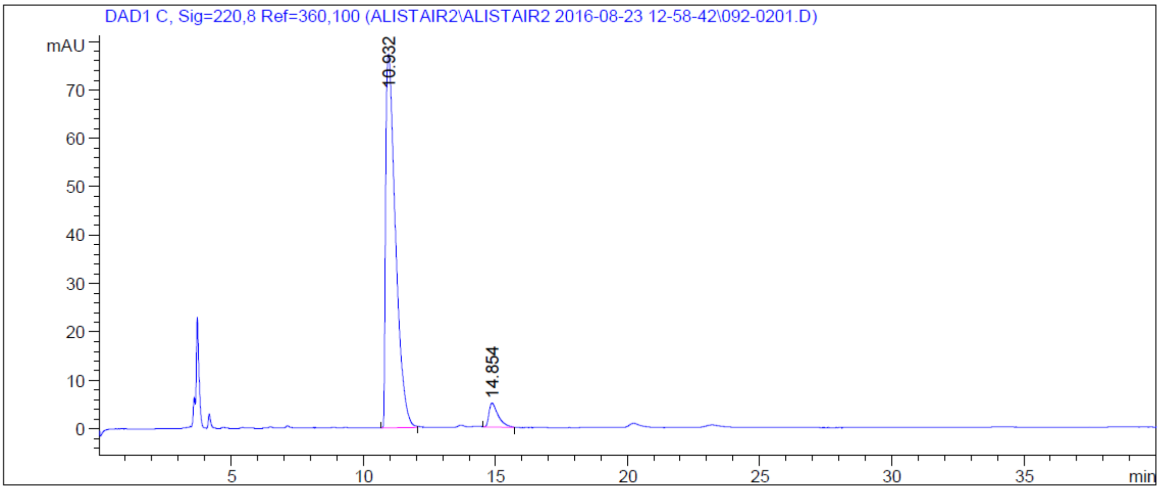

Signal 3: DAD1 C, Sig=220,8 Ref=360,100

Peak #	RetTime [min]	Type	Width [min]	Area [mAU*s]	Height [mAU]	Area %
1	10.932	BB	0.3953	1990.68152	77.08029	94.3647
2	14.854	BB	0.3596	118.88119	4.96119	5.6353
Totals :				2109.56271	82.04148	

Methyl (*S*)-3-(propylthio)butanoate **4a**

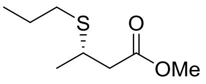

(Chiralpak IA, hexane/isopropanol = 100/0, 1.0 mL/min)

Racemic

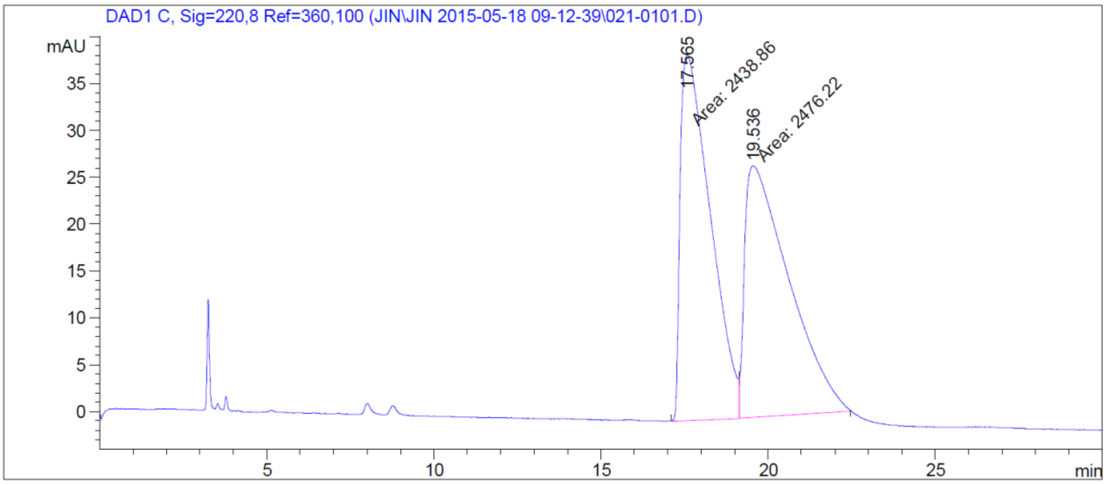

Signal 3: DAD1 C, Sig=220,8 Ref=360,100

Peak #	RetTime [min]	Type	Width [min]	Area [mAU*s]	Height [mAU]	Area %
1	17.565	MM	1.0410	2438.85913	39.04567	49.6199
2	19.536	MM	1.5400	2476.22363	26.79920	50.3801

Enantiomerically enriched (94:6 er)

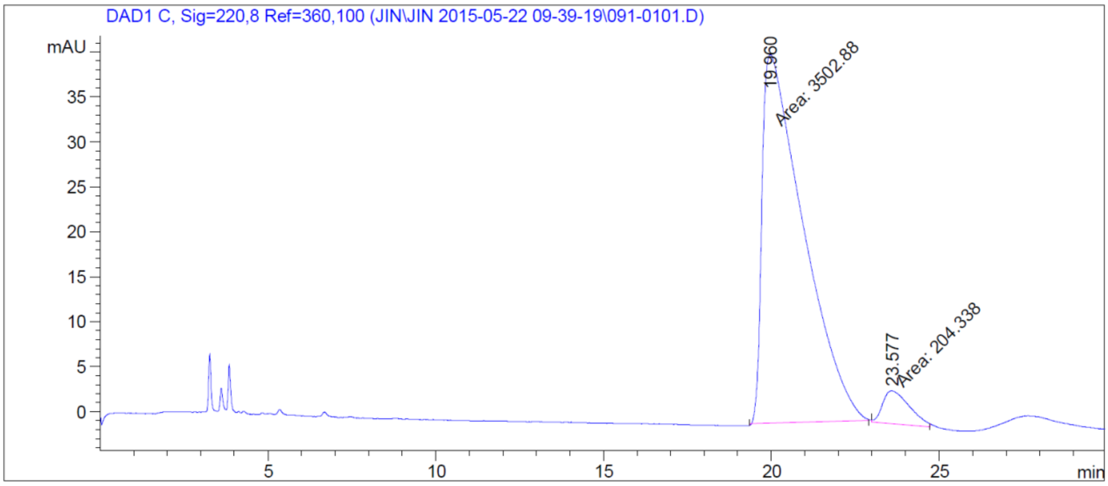

Signal 3: DAD1 C, Sig=220,8 Ref=360,100

Peak #	RetTime [min]	Type	Width [min]	Area [mAU*s]	Height [mAU]	Area %
1	19.960	MM	1.4245	3502.88037	40.98492	94.4881
2	23.577	MM	0.9312	204.33829	3.65733	5.5119

(*S*)-3-((4-Methoxybenzyl)thio)butan-1-ol **5b**

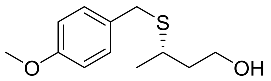

(Chiralpak AD-H, hexane/isopropanol = 97/3, 1.0 mL/min)

Racemic

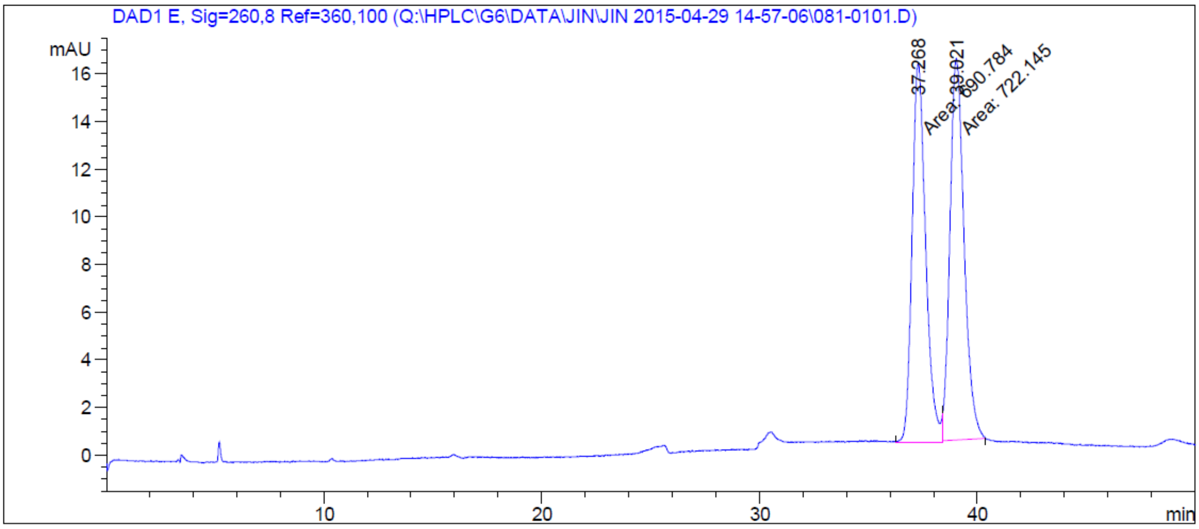

Signal 5: DAD1 E, Sig=260,8 Ref=360,100

Peak #	RetTime [min]	Type	Width [min]	Area [mAU*s]	Height [mAU]	Area %
1	37.268	MM	0.7240	690.78394	15.90182	48.8902
2	39.021	MM	0.7515	722.14496	16.01645	51.1098

Enantiomerically enriched (93:7 er)

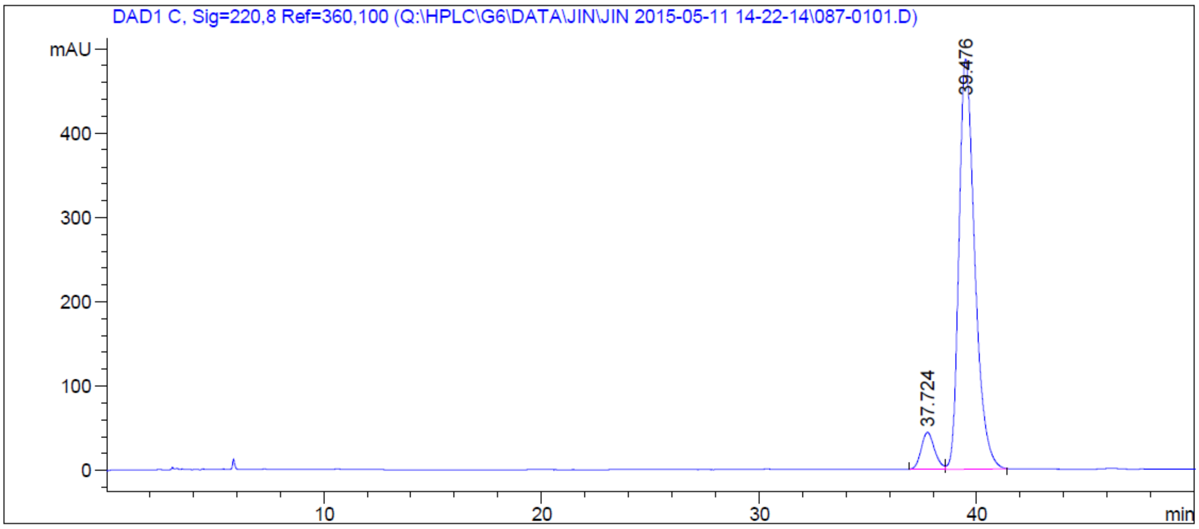

Peak #	RetTime [min]	Type	Width [min]	Area [mAU*s]	Height [mAU]	Area %
1	37.724	BV	0.6655	1886.90698	43.89383	7.1179
2	39.476	VB	0.7723	2.46224e4	486.62790	92.8821

HPLC chromatograms for Section 1.8

1,1,1,3,3,3-Hexafluoropropan-2-yl (*R*)-3-((4-methoxybenzyl)thio)-3-phenylpropanoate **12**

(Chiralpak AS-H, hexane/isopropanol = 99/1, 1.0 mL/min)

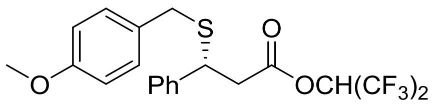

Racemic

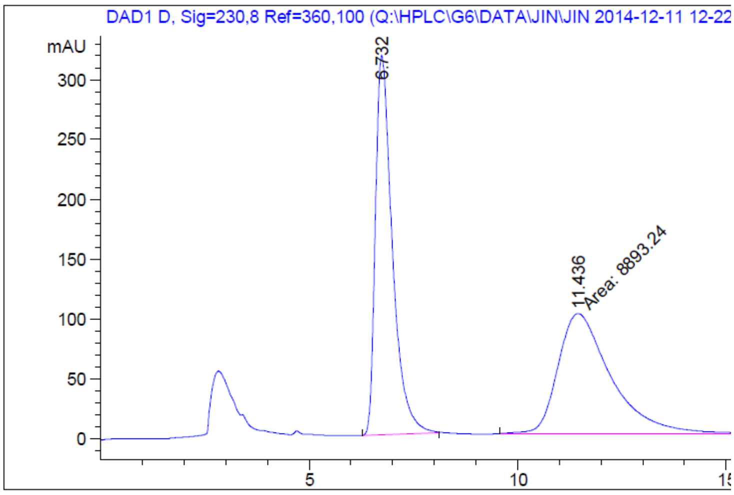

Signal 4: DAD1 D, Sig=230,8 Ref=360,100

Peak #	RetTime [min]	Type	Width [min]	Area [mAU*s]	Height [mAU]	Area %
1	6.732	BB	0.4254	8948.48828	316.98099	50.1548
2	11.436	MM	1.4749	8893.24316	100.49656	49.8452

Enantiomerically enriched (90:10 er)

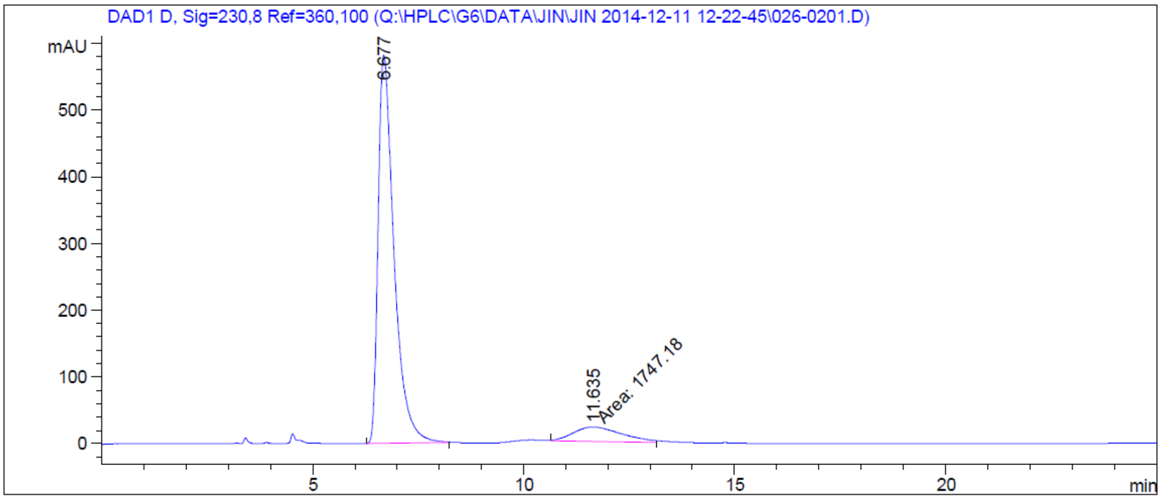

Signal 4: DAD1 D, Sig=230,8 Ref=360,100

Peak #	RetTime [min]	Type	Width [min]	Area [mAU*s]	Height [mAU]	Area %
1	6.677	BB	0.3937	1.52227e4	581.00500	89.7042
2	11.635	MM	1.3463	1747.18091	21.62953	10.2958
